# Advances in Intrauterine Contraception and Therapeutic Intrauterine Devices, 2016–2026: A Targeted Scoping Review

**DOI:** 10.3390/jcm15218338

**Published:** 2026-10-26

**Authors:** Marius Biriș, Victor Bogdan Buciu, Ioana Mihaela Cîtu, Andrei Pescaru, Răzvan Roșca, Raluca Pârvănescu, Larisa Tomescu, Denis Mihai Serban

**Affiliations:** 1 Department of Obstetrics and Gynecology, “Victor Babeș” University of Medicine and Pharmacy, 300074 Timișoara, Romania; 2 Obstetrics-Gynecology Clinic IV, Timișoara Municipal Emergency Clinical Hospital, 300074 Timișoara, Romania; 3 Doctoral School, “Victor Babeș” University of Medicine and Pharmacy, 300074 Timișoara, Romania; 4 Discipline of Methodology of Scientific Research, Department III—Functional Sciences, “Victor Babeș” University of Medicine and Pharmacy, 300074 Timișoara, Romania; 5 Department of Internal Medicine I, “Victor Babeș” University of Medicine and Pharmacy, 300074 Timișoara, Romania; 6 Obstetrics and Gynecology Clinic II, Timișoara Emergency Municipal Clinical Hospital, 300074 Timișoara, Romania; 7 Department III— Functional Sciences, Discipline of Medical Informatics and Biostatistics, “Victor Babeș” University of Medicine and Pharmacy, 300074 Timișoara, Romania

**Keywords:** intrauterine device, levonorgestrel, copper IUD, uterine perforation, IUD expulsion, postpartum insertion, emergency contraception, heavy menstrual bleeding, scoping review

## Abstract

Intrauterine contraception has changed over the past decade through longer validated duration, lower-dose hormonal devices, redesigned copper devices, new indications, and better safety surveillance. This targeted scoping review (OSF registration 10.17605/OSF.IO/3GUQF) mapped English-language primary human studies published from 1 January 2016 to 5 September 2026 in journals indexed in the Science Citation Index Expanded (SCIE). A reproducible search targeted to the major device programs, randomized trials, and large surveillance cohorts identified 766 PubMed/MEDLINE records, with no additional unique records from Scopus or Web of Science; of the 624 unique records screened, 123 reports were assessed, and 114, from 66 distinct trials, cohorts, or data sources, were included and appraised with design-specific Joanna Briggs Institute (JBI) tools. Two single-arm programs and a 7-year randomized trial supported the efficacy of 52 mg levonorgestrel intrauterine devices (LNG-IUDs) through 8 years. Lower-dose LNG-IUDs eased placement but were associated with higher ectopic pregnancy rates in four Scandinavian analyses and with less antidepressant use than the 52 mg device. Smaller or size-matched copper devices improved continuation and reduced expulsion in randomized comparisons, whereas a low-copper nitinol device had a higher 5-year pregnancy rate than expected with TCu380A. Perforation was uncommon but more frequent after postpartum insertion and during breastfeeding; immediate postpartum insertion carried the highest expulsion risk. One randomized trial and its secondary analyses supported the 52 mg LNG-IUD for emergency contraception. Therapeutic evidence was strongest for heavy menstrual bleeding and endometrial hyperplasia, whereas intrauterine ball and precious metal designs showed no consistent benefit. Progress refined rather than replaced the T-shaped IUD.

## 1. Introduction

Intrauterine devices (IUDs) are among the most effective reversible contraceptive methods, with fewer than one pregnancy per 100 users during the first year of typical use [1]. Contemporary intrauterine contraception comprises two broad categories: copper IUDs and levonorgestrel-releasing IUDs (LNG-IUDs). Because both are intrauterine devices, the term LNG-IUD is used throughout this review, consistent with current FSRH and US CDC terminology [2,3]; the term ‘levonorgestrel-releasing intrauterine system’ is retained only in cited titles. Depending on the product, currently available devices can provide approximately 3–8 years of hormonal contraception and up to 10–12 years of copper contraception, while fertility returns rapidly after removal [1,2,3]. Current WHO and CDC guidance supports use across a broad range of reproductive ages, including adolescents and nulliparous patients, and recognizes postpartum use when clinically appropriate [1,2]. This combination of high efficacy, reversibility, and minimal dependence on user adherence has made intrauterine contraception a central component of modern family planning practice.

For many years, however, the clinical concept of an IUD remained comparatively stable. The copper TCu380A and the 52 mg LNG-IUD became reference devices, most commonly built around a conventional T-shaped frame, and clinical counseling focused primarily on contraceptive effectiveness, duration, and expected bleeding patterns [4,5]; large-scale surveillance published shortly before the period of this review had shown a lower contraceptive failure rate with LNG-IUDs than with copper IUDs in routine practice [6]. Copper devices offered hormone-free long-term contraception but could increase menstrual bleeding and dysmenorrhea, whereas LNG-IUDs typically reduced bleeding over time and could induce amenorrhea [1,4]. Rare complications such as uterine perforation, expulsion, and pelvic infection were recognized, but early estimates were often derived from trials or cohorts that were too small to characterize uncommon events precisely. Similarly, although anatomical mismatch between a fixed device frame and variation in uterine cavity dimensions had long been proposed as a contributor to pain, displacement, or expulsion, device selection was not routinely individualized according to uterine geometry [4,7].

During the last decade, this relatively simple two-device paradigm has become substantially more complex. Hormonal devices have diversified into 52 mg, 19.5 mg, and 13.5 mg LNG-IUDs with different dimensions, release rates, bleeding profiles, and approved durations [2,3]. At the same time, evidence supporting extended use of 52 mg devices progressed from limited observational experience to large prospective extension programs, shifting the clinically validated contraceptive horizon from approximately 5 years to 8 years [5,8,9]. Copper device development has followed a different route, emphasizing smaller frames [10], altered copper distribution, frameless anchoring [11], three-dimensional or intrauterine ball configurations [12,13], flexible nitinol frames [14,15,16], and copper devices incorporating silver or gold [17]. These approaches reflect a broader move from maximizing contraceptive efficacy alone toward improving fit, insertion experience, bleeding, pain, expulsion, continuation, and satisfaction (Figure 1).

Clinical indications and eligible populations have expanded in parallel with device technology. Large surveillance programs such as EURAS-IUD and APEX-IUD have provided population-scale estimates for uterine perforation and expulsion and clarified the importance of postpartum timing and breastfeeding as risk modifiers [18,19]. Randomized studies have examined immediate, early, and interval postpartum placement [20,21,22], while contemporary evidence has strengthened the case for IUD use in adolescents and nulliparous patients [2,10,23]. The traditional view that the copper IUD is the only intrauterine option for emergency contraception was also challenged by a randomized noninferiority trial reporting very low pregnancy rates after placement of a 52 mg LNG-IUD for this indication [24]. At the same time, the therapeutic role of the LNG-IUD has expanded beyond routine contraception, particularly for heavy menstrual bleeding [25,26] and selected endometrial disorders such as endometrial hyperplasia and adenomyosis [27,28,29,30,31], while experimental devices have begun to explore locally delivered agents other than levonorgestrel [32].

Despite this rapid development, the literature remains fragmented. Studies are commonly organized around a single product, dose, indication, or safety endpoint, and several influential publications represent sequential follow-up or secondary analyses of the same underlying clinical programs. Consequently, the number of publications can overstate the number of independent populations studied, while highly visible technological modifications may appear more clinically important than the evidence supporting them. Previous reviews and guidelines have generally addressed individual questions such as eligibility, duration, emergency contraception, or specific therapeutic indications rather than mapping technological, pharmacological, and clinical developments together over a defined contemporary period [2,3,5]. A structured map of the major device programs and of the randomized and large surveillance evidence is therefore needed to distinguish which changes in device design and materials, hormone dose, duration of use, timing of insertion, and indication have produced measurable clinical benefit, and which remain promising but incompletely validated.

Accordingly, this targeted scoping review aimed to map and critically characterize the major device programs, randomized trials, and large surveillance cohorts on intrauterine contraception and therapeutic intrauterine devices from 2016 to 2026. We evaluated developments in LNG dose and duration, copper device design and materials, device geometry and uterine fit, contraceptive effectiveness and safety, postpartum and special-population use, emergency contraception, and therapeutic applications beyond routine contraception. A further objective was to assess the methodological maturity of the evidence and identify areas in which apparent technological innovation has, or has not, translated into clinically meaningful improvements in effectiveness, tolerability, continuation, safety, or patient experience.

## 2. Methods

### 2.1. Study Design and Methodological Framework

This study was conducted as a targeted scoping review with a reproducible literature search and design-specific critical appraisal. The review was designed as an evidence map of the major device programs and of the randomized and large surveillance evidence on clinically relevant technological, pharmacological, and clinical developments in intrauterine contraception and therapeutic intrauterine devices over the period from 1 January 2016 to 5 September 2026. The methodological approach was informed by the framework proposed by Arksey and O’Malley [33], subsequently refined by Levac et al. [34], and by the current JBI guidance for scoping reviews [35]. Reporting was guided by the PRISMA Extension for Scoping Reviews (PRISMA-ScR) [36], with the study selection process displayed using the PRISMA 2020 flow diagram structure [37].

A scoping design was selected because the review question extends across multiple device classes, materials, doses, indications, populations, insertion timings, and outcome definitions. The purpose was therefore not to estimate a single pooled treatment effect, but to map how intrauterine technology and its clinical use have evolved, identify which innovations are supported by comparative or long-term human evidence, and distinguish established advances from developments that remain exploratory or insufficiently validated. This rationale is consistent with published guidance on choosing between systematic and scoping review approaches [38]. A review protocol was developed a priori and registered with the Open Science Framework (OSF) before initiation of the literature search (DOI: 10.17605/OSF.IO/3GUQF). Supplementary Table S7 lists the elements that were fixed in the registered protocol before the search, those refined afterwards, and all deviations from the protocol, including those made in response to peer review.

### 2.2. Aim and Objectives

Primary objective. The primary objective was to map and critically characterize advances in intrauterine contraceptive devices and therapeutic intrauterine devices reported during 2016–2026, and to determine whether changes in device design, materials, active component or dose, duration of use, and timing of insertion were accompanied by clinically meaningful changes in contraceptive effectiveness, safety, tolerability, continuation, patient-reported outcomes, population of use, and therapeutic applications.

**Specific objectives.** The review question, PCC elements, information sources, and core eligibility criteria were fixed in the registered protocol before the search. The specific objectives below were refined into nine evidence domains after familiarization with the retrieved records and before data charting; this refinement changed the grouping and wording of the objectives but not the eligibility criteria (Supplementary Table S7).

1. Evolution of levonorgestrel-releasing intrauterine devices. To characterize the contemporary 52 mg, 19.5 mg, and 13.5 mg LNG-IUD platforms, including device dimensions, levonorgestrel content and release characteristics, bleeding profiles, amenorrhea, continuation, and the clinical implications of lower-dose or smaller devices.

2. Extension of duration of use. To evaluate evidence supporting use of 52 mg LNG-IUDs beyond historically established contraceptive durations, including pregnancy and ectopic pregnancy outcomes, cumulative failure, continuation, bleeding patterns, residual levonorgestrel, and safety through 7–10 years, where reported.

3. Innovation in copper intrauterine devices. To map changes in copper surface area and distribution, device dimensions, frame flexibility, insertion systems, and materials, including miniaturized copper devices, flexible or nitinol-frame systems, frameless or anchored devices, intrauterine ball designs, and copper devices containing silver or gold.

4. Device geometry, uterine fit, and patient-centered performance. To assess whether smaller, more flexible, frameless, or otherwise modified geometries are associated with differences in insertion success or device-related placement pain, expulsion, displacement or malposition, bleeding, dysmenorrhea, continuation, discontinuation, and patient satisfaction, particularly in nulliparous and adolescent users.

5. Contraceptive effectiveness and safety. To characterize pregnancy rates, the Pearl Index, or life-table estimates where available, contraceptive failure, expulsion, perforation, pelvic infection, ectopic pregnancy, serious adverse events, and device removal, with particular attention to evidence from large prospective cohorts, registries, and active-surveillance programs capable of evaluating uncommon complications.

6. Expansion of timing and eligible populations. To synthesize evidence on immediate postpartum, postplacental, intracesarean, and early-versus-interval placement, and on use in adolescents, nulliparous women, and breastfeeding or postpartum populations, with emphasis on outcomes that influence practical device selection and counseling.

7. Emergency contraception. To evaluate the emergence of the 52 mg LNG-IUD as an intrauterine emergency contraception option in comparison with the copper T380A, including short-term pregnancy risk, continuation, and subsequent one-year contraceptive outcomes.

8. Therapeutic and local drug delivery applications beyond routine contraception. To map the use of LNG-IUDs for heavy menstrual bleeding and other gynecologic indications, including comparisons with endometrial ablation and combined oral contraceptives, conservative treatment of endometrial hyperplasia and early endometrial cancer, treatment of adenomyosis and cesarean-scar niche-related bleeding, and proof-of-concept intrauterine drug delivery strategies, such as copper devices releasing ulipristal acetate or LNG-IUDs releasing indomethacin. The reported association between IUD use and a lower incidence of cervical cancer was not included in this objective because it derives from epidemiological observation rather than from a therapeutic application or device development; it is discussed as context [39].

9. Evidence maturity and unresolved gaps. To determine which apparent innovations are supported by robust comparative or long-term clinical evidence and which remain supported only by pilot, single-arm, secondary, or exploratory analyses, and to identify unresolved questions concerning direct device comparisons, individualized device selection, long-term outcomes of recently introduced copper systems, clinically meaningful benefits of alloy additions, and standardized patient-reported outcomes.

Scope. This review addressed device-related developments. Procedural interventions intended to reduce pain at placement (for example, local anesthesia, nonsteroidal anti-inflammatory drugs, misoprostol or other cervical preparation, and modified insertion techniques) were deliberately excluded because they apply across devices, have been synthesized in dedicated systematic reviews [40,41], and are addressed in current practice recommendations [42]. Placement pain was nevertheless charted as a device-related outcome whenever an included device study reported it. Established noncontraceptive uses of the 52 mg LNG-IUD that did not involve a new device or indication during the review period, namely endometrial protection during menopausal estrogen therapy and treatment of endometriosis-associated pain, were treated as context and are discussed with reference to guidelines [3,43,44] rather than mapped as primary evidence.

### 2.3. Review Question and Population–Concept–Context (PCC) Framework

The principal review question was: What technological, pharmacological, and clinical advances in intrauterine contraceptive devices and therapeutic intrauterine devices were reported between 1 January 2016 and 5 September 2026, and to what extent did these developments alter effectiveness, safety, tolerability, duration of use, device performance, patient experience, timing or population of use, and therapeutic applications?

Population. Women using, initiating, considering, or receiving an intrauterine contraceptive device or a levonorgestrel-releasing intrauterine device, including adolescents, nulliparous women, postpartum or breastfeeding women, and women receiving an LNG-IUD for a therapeutic gynecologic indication.

Concept. Innovation and evolution of intrauterine devices, encompassing device design, geometry, dimensions, uterine fit, materials, copper content and distribution, levonorgestrel dose and release, device-related insertion characteristics, duration of use, contraceptive efficacy, safety, tolerability, patient-reported outcomes, timing of insertion, special-population use, and expansion into local intrauterine drug delivery or noncontraceptive indications.

Context. Any clinical healthcare setting and any geographical region.

### 2.4. Eligibility Criteria

#### 2.4.1. Inclusion Criteria

Primary human clinical studies were eligible when they: (i) were published between 1 January 2016 and 5 September 2026; (ii) investigated an intrauterine contraceptive device, an LNG-IUD, or a clinically evaluated intrauterine drug delivery platform relevant to the review question; (iii) contributed evidence to at least one of the evidence domains defined in Section 2.2; (iv) were published as peer-reviewed full articles in English; (v) were retrievable through the information sources listed in Section 2.5; and (vi) were published in journals indexed in the Science Citation Index Expanded (SCIE). Eligible designs included randomized controlled trials, nonrandomized controlled studies, single-arm interventional trials and their extensions, prospective and retrospective cohort studies, registry and active-surveillance studies, postmarketing studies, single-group observational series of device users, other comparative observational designs, and device-content or pharmacokinetic studies nested in clinical programs. Systematic reviews and meta-analyses were used for contextualization and citation tracking only and were not entered into the principal primary-study evidence table.

Sequential publications from the same trial or cohort were retained when they reported a distinct follow-up milestone, device characteristic, safety analysis, or clinically relevant outcome. Such reports were explicitly linked to their underlying cohort so that repeated publications were not interpreted as independent patient populations.

Targeted scope. During revision, this review was reframed as a targeted map. For records identified by the targeted search (Section 2.5), the criteria above were applied together with a scope criterion defined before those records were screened: within the evidence domains, a report was eligible if it was (a) a report from a named device development or post-authorization program (phase 2–4 trials, their extensions and nested or secondary analyses, and program-specific real-world studies); (b) a randomized trial addressing a mapped domain; or (c) a large registry, claims, or active-surveillance cohort addressing device-specific safety. Smaller single- or two-center observational studies outside these categories were excluded. The 42 reports included in the original submission were retained; all but two (IDs 6 and 37, both from an independent extended-use cohort) also met this criterion. The change of scope is recorded as a deviation from the registered protocol in Supplementary Table S7.

#### 2.4.2. Exclusion Criteria

Animal studies, in vitro or purely laboratory investigations, theoretical device development reports without human clinical data, individual case reports, small case series without a substantive novel-device contribution, conference abstracts, editorials, commentaries, letters without original data, narrative reviews, study protocols without results, preprints, book chapters, theses, non-peer-reviewed material, publications without an English full text, studies published before 2016, studies unrelated to intrauterine contraception or intrauterine drug delivery, and articles published in journals not indexed in SCIE were excluded from the principal evidence synthesis. Withdrawn or retracted publications were excluded.

### 2.5. Information Sources and Search Strategy

The original search of PubMed/MEDLINE, Scopus, and Web of Science Core Collection (5 September 2026; 116 records) retrieved too few records for the review question. During revision, the corpus was therefore extended with a targeted, reproducible search designed to retrieve the major device programs and the randomized and large surveillance evidence in each mapped domain, and the review is reported as a targeted map rather than as a comprehensive one (Supplementary Table S7). The targeted search was run in PubMed/MEDLINE on 12 October 2026 with the publication window unchanged (1 January 2016 to 5 September 2026), as nine blocks: (1) LNG-IUD brand and product names; (2) LNG-IUD hormone doses combined with intrauterine terms; (3) named and novel copper devices and frameless or ball designs; (4) copper alloys, nitinol, and device shape; (5) the EURAS, APEX-IUD, and LCS12 surveillance programs; (6) extended use, release rate, and pharmacokinetics of LNG-IUDs; (7) randomized trials of IUDs for emergency contraception, heavy menstrual bleeding, endometrial hyperplasia, or adenomyosis; (8) randomized trials of postpartum, intracesarean, adolescent, or nulliparous use; and (9) cohort, registry, or randomized studies with perforation or expulsion in the title. The same blocks were run in Scopus and Web of Science Core Collection on 12 October 2026. ClinicalTrials.gov was searched on 12 October 2026 for interventional studies of intrauterine devices first posted within the window to identify ongoing or unpublished trials within the included programs. No language filter was applied.

As a check of sensitivity, the targeted PubMed/MEDLINE search retrieved all 42 reports included in the original submission. The full syntax of each block, the number of records retrieved per block and per source, and the original search are reported in Supplementary Table S3.

In the original search, syntax was adapted to the indexing structure of each database while the conceptual term set was held constant, and backward reference screening and forward citation tracking were used to identify serial or milestone publications from major trials and surveillance programs, including ACCESS IUS, the Mirena Extension Trial, EURAS-IUD, EURAS-LCS12, and APEX-IUD. For each candidate record, article title, authorship, publication year, journal, PubMed identifier when available, and DOI were cross-checked against the corresponding bibliographic or publisher record. DOI-to-title matching was verified before inclusion, and SCIE status was checked at the journal level.

### 2.6. Selection of Sources of Evidence

Records from the original search were screened independently by two reviewers (V.B.B. and M.B.) against the predefined PCC-based eligibility criteria in two stages: title/abstract screening followed by full-text eligibility assessment by two independent reviewers (V.B.B. and M.B.). The eligibility of newly identified candidate reports was confirmed against the full text. Records not published in English were screened using the English abstract, where available; potentially relevant non-English records are listed in Supplementary Table S6 so that readers can judge what the language restriction excluded. Disagreements were resolved by discussion and consensus. Because several reports represented serial follow-up or secondary analyses of the same underlying trial or cohort, the number of included publications was not treated as equivalent to the number of independent study populations.

### 2.7. Data-Charting Process and Data Items

A standardized data-charting form was used for all included reports. For the corpus, data extraction was performed independently by two reviewers and reconciled by consensus. The charted variables included first author, full article title, journal, publication year, DOI, PubMed identifier, country, single-center or multicenter status, study design, study period, sample size, patient characteristics, age and parity or nulliparity, device name and class, levonorgestrel dose, copper surface area, device dimensions, frame and material composition, comparator, timing of insertion, duration of follow-up, primary endpoint, pregnancy outcomes, Pearl Index or life-table estimates when reported, continuation, expulsion, perforation, malposition or displacement, bleeding outcomes, dysmenorrhea or pain, amenorrhea, infection, ectopic pregnancy, serious adverse events, removal or discontinuation, patient satisfaction, therapeutic indication, and the principal study conclusion.

To address nonindependence among publications, the underlying trial or cohort was recorded for every report together with an overlap indicator. Serial reports from ACCESS IUS, the Mirena Extension Trial, EURAS-IUD, EURAS-LCS12, APEX-IUD, the Kyleena Satisfaction Study, the emergency contraception trial, national registers, and other repeated cohorts were therefore treated as complementary evidence at different follow-up points or for distinct outcomes rather than as independent replication. This distinction was maintained during both descriptive counting and narrative interpretation.

### 2.8. Critical Appraisal of Individual Sources of Evidence

Although formal critical appraisal is not mandatory for scoping reviews, methodological quality was assessed to strengthen interpretation of the mapped evidence. Two reviewers (V.B.B. and M.B.) performed the appraisal independently, and disagreements were resolved by consensus. The JBI checklist for each report was chosen according to its actual design rather than its label in the source article, and Supplementary Table S1 maps every report to its tool with a one-line rationale: the checklist for randomized controlled trials was used for comparisons between randomized groups, including follow-up and secondary analyses by randomized arm; the checklist for quasi-experimental studies for nonrandomized comparisons with a concurrent control group; the checklist for cohort studies for comparative observational cohorts, registries, active-surveillance studies, and secondary analyses of trials that compared nonrandomized exposures; the checklist for case–control studies for the one case–control analysis; and the checklist for case series for single-arm interventional trials, their extensions, and single-group observational series. Device-content, pharmacokinetic, and modeling studies, for which no JBI checklist applies, were characterized descriptively. Across the 114 reports, 44 were assessed with the randomized-trial checklist, 1 with the quasi-experimental checklist, 29 with the cohort checklist, 1 with the case–control checklist, 32 with the case-series checklist, and 7 were described without scoring. Items were coded as Yes, No, Unclear, or Not Applicable. Item-level appraisal is reported for all 107 reports assessed with a checklist (Supplementary Table S1). For 10 reports added by the targeted search whose full text could not be obtained through institutional access, appraisal was based on the abstract and, where applicable, the parent-trial report, and items not reported there were coded Unclear; these reports are identified in Supplementary Table S1g.

Critical appraisal did not determine eligibility for inclusion. Instead, methodological limitations were carried forward into the evidence synthesis. Particular attention was paid to sequence generation and allocation concealment, baseline comparability, blinding where feasible, completeness of follow-up, intention-to-treat principles, outcome measurement, control of confounding, and appropriateness of statistical analysis. Where an item could not be verified from the published article, it was coded as Unclear rather than inferred. This applied particularly to the 2026 EURAS-LCS12 analysis of silver- and gold-containing devices [17], a peer-reviewed short report whose concise methods did not describe confounder handling or loss to follow-up.

### 2.9. Synthesis of Results

A formal meta-analysis was not prespecified because substantial clinical and methodological heterogeneity was expected across device classes, populations, indications, comparators, follow-up intervals, and outcome definitions. The results were synthesized using complementary descriptive, thematic, and comparative approaches. Descriptive numerical mapping summarized the publication year, study design, population, device class, follow-up duration, and major evidence domain. Thematic synthesis was organized according to the nine evidence domains defined above: LNG-IUD evolution; extended duration; copper-device innovation; device geometry, uterine fit, and patient-centered performance; effectiveness and safety; expansion of timing and populations; emergency contraception; therapeutic and local drug delivery applications; and evidence maturity and unresolved gaps. Directly comparative studies were additionally presented in structured evidence tables. Cohort overlap was explicitly considered throughout synthesis so that repeated reports from the same underlying population did not artificially increase the apparent volume or consistency of evidence.

Quantitative outcomes were reported using the measures provided by the original studies, including the Pearl Index, pregnancies per 100 woman-years, cumulative or life-table pregnancy rates, expulsion and perforation rates, continuation, discontinuation, amenorrhea, bleeding outcomes, pain or dysmenorrhea measures, and patient satisfaction. No pooled estimate was generated solely because two or more studies reported a nominally similar outcome; quantitative pooling would be considered only where populations, devices, comparators, and outcome definitions were sufficiently homogeneous.

### 2.10. Ethical Considerations

This review analyzed only published, aggregate study-level data; no participants were recruited, and no individual-level data were accessed. Ethics committee approval and informed consent were therefore not required.

## 3. Results

### 3.1. Study Selection and Evidence Profile

The targeted PubMed/MEDLINE search returned 766 records across the nine blocks (Supplementary Table S3); after removal of 140 duplicates between blocks, 626 unique records remained, two of which had no retrievable record; Scopus and Web of Science Core Collection returned the same number of records for each block (766 each), all duplicates of PubMed/MEDLINE records, and contributed no further unique records. Of the 624 records screened by title and abstract, 501 were excluded (publication type: 193, outside the mapped domains: 138, outside the targeted scope: 103, not relevant: 49, non-English: 10, retracted: seven, and withdrawn duplicate: one). The remaining 123 reports, which included all 42 reports of the original submission, were assessed for eligibility; nine were excluded, seven because the journal was not indexed in SCIE (two ESCI-indexed studies are summarized in Supplementary Table S4), and two because they were record reviews from one or two centers outside the targeted scope, leaving one hundred and fourteen included reports (Figure 2). ClinicalTrials.gov returned 282 registered studies, which were screened for ongoing or unpublished trials within the included programs (Supplementary Table S5). The one hundred and fourteen reports came from sixty-six distinct trials, cohorts, or data sources; the largest contributors were ACCESS IUS (twelve reports), the emergency contraception trial and its observational extension (seven), APEX-IUD (six), and the Kyleena Satisfaction Study (four), so participant counts were not summed across publications. Fifty-seven reports (50.0%) were published in 2022–2026. By evidence domain, thirty-one reports concerned LNG-IUD dose, duration, or pharmacology, nineteen concerned copper-device design, nineteen concerned population-level safety, twelve concerned postpartum timing or insertion technique, three concerned adolescents, seven concerned emergency contraception, and twenty-three concerned therapeutic or local drug delivery applications. Forty-four reports were assessed with the randomized-trial checklist, twenty-nine with the cohort checklist, thirty-two with the case-series checklist, one each with the quasi-experimental and case–control checklists, and seven were mechanistic or modeling studies.

### 3.2. Hormonal IUD Evolution and Extension of Duration

Hormonal IUD development followed two parallel directions: lower-dose/smaller devices and prolonged use of 52 mg devices. Liletta release declined from an estimated 19.5 µg/day initially to 9.8 µg/day at 5 years [45]; Mirena population pharmacokinetic modeling estimated approximately 7.0 µg/day at 8 years, similar to the 19.5 mg device at 5 years (7.6 µg/day) and above the 13.5 mg device at 3 years (5.5 µg/day) [46]. An integrated pharmacokinetic analysis of more than 3400 participants found systemic LNG exposure to be lowest with LNG-IUDs, rising with release rate across the 13.5, 19.5, and 52 mg devices and remaining below that of the LNG implant and oral contraceptives [47].

For the 19.5 mg LNG-IUD, phase III follow-up showed a 5-year Pearl Index of 0.29 and cumulative failure of 1.4%, with expulsion, perforation, and pelvic inflammatory disease in 3.7%, 0.2%, and 0.6% [48]; in pooled diaries, the proportion with no more than four bleeding or spotting days per month rose from 6.2% in month 1 to 54.1% at month 12 [49], and a model trained on these data correctly classified about 70% of users’ later bleeding pattern [50]. In routine practice, the multinational Kyleena Satisfaction Study (n = 1129) reported easy insertion in 92.2% of parous and 89.9% of nulliparous women and no or mild pain in 88.6% and 67.4% [51,52], with 12-month satisfaction of 86.1% and continuation of 81.4% [53], as well as similar results in its German subset [54]. The 13.5 mg device was evaluated in phase III studies in adolescents (placement 99.7%; no pregnancies in 12 months) [55], and in an Asia Pacific population (Pearl Index 0.35; 3-year failure 0.9%) [56], with placement success of 99.5% in a pooled, mostly nulliparous population [57]. In randomized comparisons with other methods, its 12-month discontinuation was lower than with the etonogestrel implant (19.6% vs. 26.8%) [58], and satisfaction was similar to that with a combined oral contraceptive (82.1% vs. 81.9%) [59]; against a copper device (Nova T Cu380, Finland, Bayer), it produced fewer bleeding days at 36 months (median 4 vs. 15/90 days), much lower PBAC scores (7.9 vs. 126), and 40% vs. 0% amenorrhea [60], with better health-related quality of life [61]. In US claims data, 12-month discontinuation was similar to that with the implant [62]. These comparisons are with non-intrauterine or non-hormonal methods and do not isolate an effect of lower LNG dose; within the included evidence, the only randomized comparison of a lower-dose device with the 52 mg device was the adolescent trial described in Section 3.4 [23,63].

A lower hormone dose did not confer a uniform safety gradient, and dose was associated in opposite directions with different outcomes. Four analyses from Denmark and Sweden found higher ectopic pregnancy rates with the 13.5 mg device: in the Danish nationwide cohort (963,964 women), rates were 7.7, 7.1 and 15.7 per 10,000 person-years for the 52, 19.5, and 13.5 mg devices (adjusted rate ratio 2.3 for 13.5 vs. 52 mg) [64]; in the Swedish prescribed drug register (1,663,242 women), rates were 2.76 versus 0.30 per 1000 woman-years for the 13.5 and 52 mg devices [65]; and two Stockholm hospital cohorts with overlapping catchments estimated ectopic Pearl Indices of 0.19 versus 0.006 [66], and 0.136, 0.037, and 0.009 for the 13.5, 19.5 and 52 mg devices [67]. Conversely, in a Danish cohort of 46,565 nulliparous first-time users, antidepressant initiation was lower with the 19.5 mg (rate ratio of 0.77) and 13.5 mg (0.85) devices than with the 52 mg device [68], and a French matched cohort reported slightly more antidepressant use with the 52 mg device than with the 19.5 mg device [69]. All of these analyses were observational.

Within the ACCESS IUS program for the 52 mg Liletta device, secondary analyses characterized outcomes beyond efficacy: amenorrhea in 16.9% of women not previously using an LNG-IUD at 12 months [70], and in 19% and 37% of all users at 1 and 3 years [71]; expulsion in 3.8% over 6 years, more often in parous women with only vaginal deliveries (7.2%) and in women with obesity [72]; pelvic infection in 0.5% over 2 years with same-day testing for sexually transmitted infection and placement [73]; conception within 12 months of removal in 86.1% of women attempting pregnancy [74]; and hormone-related side effects leading to discontinuation in 5.5% [75]. Because all derive from a single single-arm cohort, they describe one population rather than independent confirmation.

The clearest longitudinal advance was the extension of 52 mg LNG-IUD contraception (Table 1). The WHO randomized trial in parous women provided a head-to-head comparison with TCu380A through 7 years [76]. A selected Campinas cohort of 776 women who continued the same Mirena beyond 5 years reported no pregnancies over 967 woman-years through 7 years and 1485 woman-years through 15 years [77], although later-year numbers were small, and a prospective US cohort of 496 women continuing a 52 mg device beyond 5 years reported low failure rates in years 6 and 7 [78]. Two prospective programs then extended efficacy data for the two 52 mg products to 8 years: for Liletta, the ACCESS IUS phase 3 trial, with serial reports at 5, 6, and 8 years [8,79,80] and follow-up of 339 users beyond 8 years [81]; and for Mirena, the Mirena Extension Trial, whose estimates for years 6–8 apply only to women who had completed 5 years of use [9]. Residual LNG content after more than 8 years of use was quantified in removed devices [82]. Because the 8-year evidence comes from two single-arm programs with substantial attrition after year 5 (Supplementary Table S1c), it supports efficacy through 8 years without the certainty of a randomized comparison.

### 3.3. Copper-Device Innovation, Geometry, and Uterine Fit

Copper-device innovation was more heterogeneous. The original SCu380A intrauterine ball pilot (n = 51) had 27% expulsion and 16% symptom-related removal at 12 months [13], whereas later Ballerine MIDI observational cohorts reported expulsion of 5.3% in Switzerland [83] and 3.4% in Israel [84], with pregnancy rates of 1.4% and 0.57%, respectively. In the larger EURAS-LCS12 shape analysis (26,381 users) [12], however, adjusted expulsion hazards versus Tatum-T were 0.8 for Nova-T, 1.3 for frameless devices, 1.6 for Multiload, and 3.6 for intrauterine balls, suggesting that unconventional geometry was not inherently advantageous; as an observational surveillance analysis, it remains open to residual confounding, for example, by inserter experience. A secondary EURAS-IUD analysis of 5762 users younger than 30 years similarly associated lower copper surface area (<300 mm^2^), horseshoe frames, intermediate widths, and flexible arms with higher 1-year continuation and fewer unwanted effects [85].

Anchoring showed a context-specific signal postpartum. Intracesarean Gyn-CS reduced 3-month expulsion compared with TCu380A (Figure 3; *p* = 0.039) [11], although the trial was underpowered; two Gyn-CS series reported one expulsion among 100 users [86] and none among 21 users with an added serosal fixation suture [87], and a 14-hospital Chinese cohort of the anchored GyneFix postpartum device placed at cesarean delivery reported 12-month expulsion of 7.6% and continuation of 86.6% [88]. Among framed devices, size reduction had the strongest randomized support. The NTCu380-Mini had higher 12-month continuation and lower expulsion and bleeding/pain-related removal than TCu380A in nulliparous users (Figure 3) [10], and at 3 years, bleeding/pain discontinuation (14.5% vs. 26.8%) and expulsion (10.0% vs. 15.1%) remained lower [89]; a secondary analysis of the same trial found that concurrent menstrual cup use was associated with expulsion with the NTCu380-Mini (adjusted OR 3.13) but not with TCu380A [90]. Pregnancy rates were not reported in the 12-month interim report, which deferred efficacy outcomes to the final analysis [10], nor in the 3-year secondary analysis [89]; the primary efficacy report of this trial appeared in a journal outside SCIE and is summarized in Supplementary Table S4. In a 600-woman Indian trial, a TCu380Ag device offered in three sizes matched to uterocervical length had higher 5-year continuation and fewer expulsions than the single-size TCu380A (Figure 3), with no pregnancies in either group [91].

The Cu 175 mm^2^ flexible nitinol-frame device was first compared with a copper T380S in a participant-blinded phase 2 trial in 300 parous women, with less insertion pain, higher 12-month continuation, fewer expulsions, and fewer removals for pain or bleeding (Figure 3) [14]; a separate phase 2 study reported a 36-month Pearl Index of 0.46 and expulsion of 1.8% [92]. In the single-arm phase 3 program, placement success was 98.8% among 1620 participants, with a 3-year pregnancy rate of 2.47% [15]; by 5 years, the cumulative Pearl Index was 1.02, life-table pregnancy was 4.1%, and expulsion was 4.7%, with bleeding/pain-related discontinuation falling from 8.7% in year 1 to 1.9% in year 5 [16]. This 5-year pregnancy rate is higher than that observed with TCu380A in comparative trials (for example, 2.45 per 100 over 7 years in the WHO trial [76]), indicating that the lower copper load may trade some contraceptive efficacy for tolerability, although such cross-trial comparisons are approximate. Because the only randomized comparison was small, used T380S rather than TCu380A, and was not powered for pregnancy, neither superiority nor noninferiority to TCu380A can be inferred. In EURAS-LCS12 (28,280 users) [17], unintended-pregnancy and PID incidence were similar for copper-only, copper–silver, and copper–gold devices, while product technical complaints were highest with copper–gold devices (1.20/100 woman-years vs. 0.09 for copper-only).

All current technologies are better visualized in Table 2.

### 3.4. Safety, Postpartum Timing, and Special Populations

Large surveillance cohorts quantified rare harms more precisely than conventional trials (Figure 4). EURAS-IUD compared the 52 mg LNG-IUD (Mirena) with copper IUDs of various designs [18], and APEX-IUD included both LNG-IUDs and copper IUDs, analyzed as device classes rather than as individual products [19,93]; both found perforation to be uncommon. In EURAS-IUD, complete perforation occurred in 4.5 versus 0.6 per 1000 insertions in lactating and non-lactating women [94]. In APEX-IUD (n = 326,658), perforation risk was lowest for nonpostpartum insertions (Figure 4) and highest for insertion 4 days to ≤6 weeks postpartum (aHR: 6.71). Breastfeeding increased risk (aHR: 1.37) [19,95]. In the device-type analysis (n = 322,898), LNG-IUDs had a slightly higher 5-year perforation incidence than copper IUDs (absolute difference: 0.08 percentage points; Figure 4), whereas expulsion was 4.52% versus 4.82% [93]. APEX-IUD also quantified expulsion: risk was highest after insertion 0–3 days postpartum (5-year cumulative incidence: 10.73%; aHR: 5.34 vs. nonpostpartum) and lower in breastfeeding women (aHR: 0.71) [96]; among nonpostpartum users, a recent diagnosis of heavy menstrual bleeding was the strongest risk factor (aHR: 2.84), and higher body mass index, age of 24 years or younger, and parity of four or more were also associated with expulsion [97,98].

Population-based cohorts also addressed systemic safety questions relevant to hormonal devices. For breast cancer, an Israeli health maintenance organization cohort of 13,354 users aged 40–50 years found a non-significant excess of invasive breast cancer at 5 years (1.06% vs. 0.93%; *p* = 0.051), concentrated in women aged 40–45 years [99], and a Swedish nationwide cohort of 2,095,130 girls and women found a hazard ratio of 1.13 (95% CI: 1.09–1.18) for the 52 mg LNG-IUD, corresponding to about eight additional cases per 100,000 person-years and lower than for desogestrel-containing methods [100]. For mood, a French propensity-matched cohort found slightly more anxiolytic initiation with the 52 mg LNG-IUD than with copper IUDs (HR: 1.08) and no difference in ectopic pregnancy, pelvic infection, or perforation [101], and the dose comparisons in Section 3.2 found more antidepressant use with the 52 mg device than with lower-dose devices [68,69]. A French national case–control study found no excess risk of intracranial meningioma with the 52 mg (OR: 0.94, 95% CI: 0.86–1.04) or 13.5 mg (OR: 1.39, 0.70–2.77) devices [102]. These observational associations are susceptible to residual confounding, and absolute differences were small.

Postpartum trials showed a consistent access–retention trade-off. Immediate LNG-IUD placement was noninferior to delayed placement at 4–8 weeks for breastfeeding outcomes (79% vs. 84% breastfeeding at 8 weeks) but produced substantially more expulsions (19% vs. 2%) [20]; 17 of the 24 women with an expulsion (71%) had the device reinserted, whereas 24 women assigned to delayed placement did not return for insertion, and IUD use at 6 months by group was not reported; in a substudy, milk LNG concentrations were low in both groups and milk fat content was unaffected [103], and intracesarean LNG-IUD placement did not alter breastfeeding at 6–24 weeks [104]. After vaginal delivery, a Swedish trial of LNG-IUD placement within 48 h was stopped early when 44.2% of devices had been expelled, although after reinsertion, 75% of women were still using the method at 1 year (vs. 83.7% with placement at 6–8 weeks) [105]. Postplacental TCu380A versus LNG-IUD expulsion was 36.7% versus 20.0% at 90 days [21] and 39.4% versus 22.2% at 1 year, with 1-year continuation of 54.4% versus 73.1% [106]; vaginal delivery was a stronger predictor than device type. Later timing mattered less: placement at 3 rather than 6 weeks did not change IUD use at 3 months (73.0% vs. 75.3%) [107], and in the early-versus-interval trial, complete expulsion at 6 months was 2.0% after placement at 2–4 weeks and 0% after placement at 6–8 weeks, meeting the prespecified noninferiority margin, whereas partial expulsion (9.4% vs. 7.6%) did not meet the noninferiority criterion [22].

The insertion technique was tested in several copper device trials. A dedicated postpartum inserter did not reduce complete expulsion compared with forceps (7.9% vs. 5.4%) and produced more partial expulsions [108]; in a secondary analysis, expulsion was 3.2% after postplacental insertion and 7.5% after later immediate-postpartum insertion (*p* = 0.176) [109]. Manual and ring-forceps postplacental insertion had similar complete expulsion (16.7% vs. 25%; *p* = 0.168), with less discomfort using forceps [110]. At cesarean delivery, an introducer-withdrawal technique reduced 6-month displacement (11.9% vs. 20.1%) and non-visible strings (3.6% vs. 9.6%) and increased continuation (88.1% vs. 79.9%) compared with manual placement [111], whereas changing the device shape (Cu375 vs. CuT380A) did not change 6-week expulsion (5.3% vs. 6.7%) [112]. Several of these trials analyzed only participants who completed follow-up or did not perform blinded outcome assessment (Supplementary Table S1a).

Adolescents and nulliparous users generally showed high continuation with device-specific differences rather than a population-level contraindication. In 318 adolescents randomized to TCu380A, the 52 mg LNG-IUD or the 19.5 mg LNG-IUD [23], 1-year continuation was higher and expulsion lower with both LNG-IUDs than with TCu380A (Figure 3). Dysmenorrhea was reported by 67.9%, 33.3%, and 36.0%, respectively, and satisfaction ranged from 80.7% with TCu380A to 97.8% with the 52 mg LNG-IUD; in the same trial, placement pain was slightly higher, and placement was less often easy with the 52 mg device than with the copper and 19.5 mg devices [63]. In a single-arm phase III study of 304 adolescents, the 13.5 mg device was placed successfully in 99.7%, with no pregnancies and 83.9% satisfaction at 12 months [55].

### 3.5. Emergency Contraception

The 52 mg LNG-IUD emerged as a possible new intrauterine emergency contraception option, supported by a single randomized trial. In this noninferiority trial [24], 1-month pregnancy occurred in 1/317 LNG-IUD users (0.3%) and 0/321 TCu380A users; the absolute difference of 0.3 percentage points (95% CI: −0.9 to 1.8) met the prespecified noninferiority margin of two percentage points, but the upper confidence limit lay close to that margin, and pregnancy after emergency placement is rare. At 1 year in the same cohort [113], pregnancy remained similar (2.8% vs. 3.0%; RR: 0.9, 95% CI: 0.4–2.2), and continuation was 62.4% versus 55.8%. Further analyses of this trial population addressed practical questions: no pregnancies occurred among the 286 participants who had intercourse within 7 days of placement [114], or among those reporting multiple episodes or episodes 6–14 days earlier of unprotected intercourse [115]; pooled with three observational protocols, one pregnancy occurred among 187 women given an LNG-IUD 6–14 days after intercourse (0.5%) [116]; against modeled conception probabilities, the LNG-IUD prevented 93.2–95.7% expected pregnancies and the copper IUD prevented 100% [117]; switching to the other device by 6 months occurred in 2.1% versus 5.5% [118]. Because these analyses draw on one trial population, they refine rather than replicate its results; a larger registered study is ongoing (Supplementary Table S5).

### 3.6. Therapeutic and Local Drug Delivery Applications

Heavy menstrual bleeding (HMB) was the dominant therapeutic indication (Table 3). In the ACCESS IUS HMB subgroup (n = 150), most women no longer reported HMB within six cycles [119], and a dedicated phase III trial (n = 105) showed near-complete reduction of objectively measured blood loss by cycle 3, with no material difference by obesity or parity [25]. In a small, randomized trial, the LNG-IUD reduced menstrual blood loss more than norethisterone or tranexamic acid [120], and a second 52 mg product (Levosert) was noninferior to Mirena for 6-month blood loss reduction [121].

Comparative effectiveness depended on the comparator and the time horizon. In the ECLIPSE primary-care trial (n = 571), condition-specific quality of life improved similarly with the LNG-IUD and usual medical treatment at 5 years (*p* = 0.09), with surgery-free survival of 80% and 77% [122]; at 10 years, 28% of 206 respondents were using an LNG-IUD, and surgery did not differ between the original groups [123]. The LNG-IUD and combined oral contraceptives both improved HMB in a pragmatic trial, with no significant intention-to-treat difference at 6 or 12 months [124]. In MIRA, both the LNG-IUD and endometrial ablation reduced bleeding, but the LNG-IUD did not meet the prespecified noninferiority criterion for PBAC at 24 months and surgical reintervention was more frequent with an LNG-IUD-first strategy [26]; this difference persisted at a mean 7.4-year follow-up, whereas hysterectomy did not differ significantly (11.8% vs. 18.1%) [125], and a parallel cohort of women who declined randomization showed the same pattern [126].

For endometrial hyperplasia without atypia, the LNG-IUD and vaginal micronized progesterone achieved similar 3-month regression [30], and after successful treatment with either the LNG-IUD or oral medroxyprogesterone acetate, relapse within 24 months of stopping therapy was common and did not differ between regimens [127]. For atypical endometrial hyperplasia, one trial (n = 148) found higher 12-month complete regression with the LNG-IUD than with megestrol acetate (*p* = 0.026), with earlier regression and less weight gain [27], whereas two smaller phase II trials found no significant difference (16-week complete response: 35.0% vs. 19.2% [31]; 9-month regression: 88.9% overall [128]). In women with obesity and atypical hyperplasia or stage 1, grade 1 endometrial adenocarcinoma treated with an LNG-IUD, with or without metformin or weight loss, the pathological complete response at 6 months was 82% for hyperplasia and lower for adenocarcinoma [129]; adding an LNG-IUD to megestrol acetate for early endometrial cancer did not improve 16-week complete response rates (25.0% vs. 19.2%) and increased vaginal bleeding [130]. For niche-related postmenstrual spotting after cesarean delivery, the LNG-IUD was not superior to hysteroscopic niche resection at 6 months [131] but was superior at 3 years [132]. The adenomyosis comparison found improvement with both the LNG-IUD and dienogest, but dienogest produced lower pain scores and better control of uterine-volume growth [28]; because allocation used odd/even visit dates and missing observations were excluded, comparative inference was limited.

A 29-participant Cu-UPA proof-of-concept study [32] demonstrated dose-related reductions in bleeding-only days across the 5, 20, and 40 µg/day UPA doses. Ovulation persisted in most observed cycles, while progesterone-receptor-modulator-associated endometrial changes occurred in 10%, 10%, and 44% at removal. No serious adverse events occurred; this remained pharmacodynamic proof-of-concept rather than contraceptive efficacy evidence.

A second combined-delivery concept added an indomethacin reservoir to the 13.5 mg LNG-IUD. In a dose-finding phase II trial (n = 174), the low-dose combination reduced bleeding and spotting days in the first 90 days relative to the unmodified device [133], and pharmacokinetic sampling showed that the two drugs were released at independent rates, with low systemic indomethacin exposure and an unchanged LNG profile [134]. Both combined-delivery concepts remain at the proof-of-concept stage.

### 3.7. Methodological Maturity and Evidence Gaps

JBI appraisal of the 107 reports appraised at item level identified methodological strengths alongside limitations typical of device trials (Supplementary Table S1). Among the forty-four randomized reports, thirty clearly used true randomization, twenty-six clearly concealed allocation, twelve had blinded participants, none blinded those delivering treatment, and nine blinded outcome assessors; thirty-two had adequate follow-up, and fifteen clearly analyzed participants in the groups to which they were randomized (Table S1a). Among the 29 cohort reports, strategies to deal with confounding were stated in 19, and follow-up was complete or adequately addressed in 18 (Table S1b). Among the 32 single-arm or single-group reports appraised with the case-series checklist, all had clear inclusion criteria and clearly reported outcomes, but none clearly reported consecutive inclusion and 16 clearly reported complete inclusion of eligible participants; by design, none had a concurrent comparator (Table S1c). The single nonrandomized controlled comparison used predictable allocation by visit date and excluded missing observations (Table S1d), the case–control study matched controls appropriately but relied on dispensing records for exposure (Table S1f), and the seven mechanistic or modeling reports were characterized descriptively (Table S1e).

Maturity was judged descriptively from study design, the number of independent programs, sample size, and the appraisal findings above; it is not a formal certainty rating. Evidence was most mature where it rested on randomized comparisons or very large cohorts with favorable appraisal: absolute perforation and expulsion risk and their postpartum, breastfeeding, and heavy menstrual bleeding modifiers (two independent surveillance programs with confounding addressed; Table S1b) [18,19,93,96,97], HMB treatment (several randomized and single-arm trials; Table S1a,c) [25,26,119,120,121,122,124,125], and selected randomized comparisons of copper devices (Table S1a) [10,89,91]. The efficacy of 52 mg LNG-IUDs through 8 years rests on two independent single-arm programs with appropriate outcome measurement but substantial attrition (Table S1c) [8,9], consistent with a 7-year randomized comparison and a prospective extended-use cohort [76,78]. Dose-related associations with ectopic pregnancy and antidepressant use rest on observational analyses from a small number of national data sources [64,65,67,68,69]. Emergency use of the 52 mg LNG-IUD rests on a single randomized trial and its secondary analyses (Table S1a) [24,113,114,117]. Evidence remained least mature for years 9–10 of LNG-IUD use, measurement-guided individualized device selection, silver/gold alloys, long-term comparative outcomes for the Cu 175 mm^2^ nitinol device, early endometrial cancer, and non-LNG intrauterine drug delivery. Ongoing registered trials (Supplementary Table S5) may help close some of these gaps, including further follow-up of the nitinol-frame low-dose copper device, a larger emergency contraception study of the 52 mg LNG-IUD, and a comparative study of a spherical copper device.

### 3.8. Overall Evidence Map

The 2016–2026 evidence therefore mapped progress along several measurable trajectories rather than identifying a single successor to the conventional T-shaped IUD: longer validated use of 52 mg LNG-IUDs; smaller hormonal and copper devices with different bleeding, pain, continuation, and safety profiles; better population-scale quantification of local and systemic safety; broader postpartum, adolescent, and emergency contraception use; and expansion of the intrauterine route into HMB, endometrial disease, niche-related bleeding, and experimental local drug delivery. The flexible nitinol-frame low-dose copper device comes closest to a new device concept, because it changes both frame material and copper load, but it remains T-shaped, and its phase 3 evidence is single-arm. The most reproducible advances were supported by randomized head-to-head studies or large surveillance cohorts; several visually or materially novel concepts did not demonstrate equivalent clinical benefit.

## 4. Discussion

### 4.1. Principal Findings

Between 2016 and 2026, intrauterine contraception advanced mainly by refining the conventional T-shaped IUD. The major changes were extension of 52 mg LNG-IUD use, diversification into smaller and lower-dose hormonal devices, renewed development of smaller and more flexible copper devices, better population-level estimates of perforation and expulsion, broader use in adolescents, nulliparous, and postpartum patients, randomized evidence for the 52 mg LNG-IUD as emergency contraception, better characterization of systemic safety signals, and expansion of LNG-IUDs as local therapeutic devices [4,5,8,9,10,19,23,24,25,27,68,76,89,93,96,100]. Compared with the older literature, the question has shifted from whether IUDs are effective to which device, dose, timing, and indication best fit the individual patient [3,23,135].

This review also shows that technological novelty does not necessarily translate into clinical benefit. Reproducible gains were seen with extended 52 mg LNG-IUD duration and with smaller or size-matched copper devices in selected populations, whereas spherical copper designs and the addition of silver or gold did not show similarly consistent advantages [8,9,10,12,13,17,83,84,89,91]. Because contraceptive failure is already uncommon with modern IUDs [1,76], continuation, bleeding, pain, expulsion, and satisfaction increasingly determine real-world value [10,23].

### 4.2. Hormonal Systems and Extended Duration

Hormonal IUD development during this decade was evolutionary. The 52 mg LNG-IUD was already an established, highly effective contraceptive and treatment for heavy menstrual bleeding, but the evidence base expanded through lower-dose 19.5 mg and 13.5 mg devices and stronger long-term data for the 52 mg platform [4,5,8,9,45,46,48,52,60,76,77,82]. Pharmacological studies showed a gradual decline in LNG release rather than abrupt exhaustion, with systemic exposure lowest for the lowest-dose device [45,46,47]; release estimated at 8 years remained comparable with that of the 19.5 mg device at the end of 5 years and above that of the 13.5 mg device at the end of 3 years [46]. This provides biological support for prolonged efficacy but cannot establish contraceptive duration by itself.

Lower-dose devices mainly broaden choice through smaller dimensions and different bleeding profiles rather than proven superiority. Five-year data established high efficacy for the 19.5 mg LNG-IUD, and real-world placement was generally easy and well tolerated, including in younger and nulliparous users [48,52,53]. The randomized 13.5 mg trial used a copper comparator [60], so its bleeding advantages reflect the hormonal mechanism and cannot be attributed to the lower dose; direct randomized comparison with the 52 mg device was limited to adolescents [23,63]. The dose also involves trade-offs in safety. Four Danish and Swedish analyses found higher ectopic pregnancy rates with the 13.5 mg device than with the 52 mg device [64,65,66,67], and a larger French registry study, excluded here only because of journal indexing, pointed in the same direction (Supplementary Table S4); for the 19.5 mg device, the rate was similar to that of the 52 mg device in Danish data but was higher in a Stockholm cohort [64,67]. Conversely, antidepressant use was lower with both lower-dose devices [68,69]. Absolute risks were low, and causality cannot be inferred, but dose reduction should be presented to patients as a trade-off rather than as intrinsically safer.

Extended duration is one of the clearest changes in practice. The earlier literature supported 7 years only cautiously [5]. The WHO randomized study provided head-to-head data through 7 years [76], and a Campinas cohort observed no pregnancies among long-term continuers, although selection and small numbers at later durations limit inference [77]; a prospective US cohort likewise reported low failure rates in years 6 and 7 [78]. Two prospective programs, one for each 52 mg product, then supported efficacy through 8 years [8,9]. Both were single-arm and lost a substantial proportion of participants after year 5, and the Mirena Extension Trial estimates apply only to women who had completed 5 years of use (Table 1); the evidence is therefore consistent and independent but not randomized. Current FSRH guidance supports 8-year contraceptive use of 52 mg LNG-IUDs inserted before the age of 45 years [3]. Evidence for years 9–10 remains exploratory [81]; residual LNG beyond 8 years supports plausibility but is not equivalent to efficacy evidence [82]. Contraceptive duration must also remain distinct from therapeutic duration, including treatment for heavy menstrual bleeding and endometrial protection during menopausal estrogen therapy [3,43].

### 4.3. Copper Innovation, Geometry, and Uterine Fit

Copper-device development was more heterogeneous. The most convincing modern signal concerns device size and mechanical compatibility rather than copper quantity [10,89,91]. In nulliparous users, the smaller NTCu380-Mini improved 12-month continuation and reduced both bleeding/pain removal and expulsion compared with TCu380A [10]; at 3 years, its advantages in bleeding/pain-related discontinuation and expulsion persisted, with higher satisfaction despite convergence of overall discontinuation [89]. A three-size TCu380Ag device matched to uterocervical length also improved 5-year continuation and reduced expulsion in a single trial [91]. These results are consistent with earlier anatomical work associating uterine cavity dimensions with expulsion or displacement [7], but they do not yet validate routine ultrasound-guided device matching; a true ‘uterine fit’ strategy would require prospective cavity measurement and randomized comparison of algorithm-guided versus standard device selection. The menstrual cup interaction observed with the smaller Nova-T-framed NTCu380-Mini [90] shows that a smaller frame can also change how a device responds to external traction, which belongs in counseling.

The Cu 175 mm^2^ flexible nitinol-frame device had high placement success and low expulsion at 3 and 5 years, but its 5-year pregnancy rate was higher than would be expected with TCu380A [15,16,76]. Less copper may improve tolerability, but apparently at the cost of lower contraceptive efficacy, and this trade-off should be stated plainly in counseling. Its flexible shape-memory frame is a plausible explanation for good retention across different cavity sizes, and a small phase 2 trial against T380S found better continuation and less pain- or bleeding-related removal [14]; however, the phase 3 program was single-arm, so historical TCu380A comparisons can establish neither superiority nor equivalence. The device is better described as a low-copper alternative for users who prioritize tolerability than as a replacement for TCu380A.

Other innovations were less consistent. The original SCu380A intrauterine ball pilot had high early expulsion and symptom-related removal [13], whereas later Ballerine observational studies reported better continuation and lower expulsion [83,84]. However, the large EURAS-LCS12 analysis found the highest adjusted expulsion hazard for intrauterine balls, whereas Nova-T and Tatum-T frames performed better [12]. Frameless anchoring may be more context-specific: in the small intracesarean randomized trial, Gyn-CS reduced 3-month expulsion compared with TCu380A [11], and anchored devices had low expulsion in cesarean series and cohorts [86,87,88]; however, large routine-practice data did not show a general frameless advantage [12]. Finally, silver- and gold-containing copper IUDs did not reduce unintended pregnancy or pelvic inflammatory disease compared with copper-only devices [17]. Across the decade, geometry, size, and flexibility therefore produced stronger clinical signals than precious metal alloying or novelty of shape alone.

### 4.4. Population Expansion, Postpartum Timing, Safety, and Emergency Contraception

The evidence also consolidated a move away from parity-based caution. Contemporary WHO and CDC guidance generally permits IUD use in adolescents and nulliparous patients [2,135]. In the randomized adolescent study, 1-year continuation was lower with TCu380A than with either LNG-IUD; copper users had more bleeding/pain and expulsion, whereas hormonal users had shorter bleeding, less dysmenorrhea, and greater satisfaction [23], and the 13.5 mg device was well accepted by adolescents in a single-arm study [55]. Together with the NTCu380-Mini trial [10,89], these findings support counseling based on patient priorities (hormone avoidance, bleeding tolerance, duration, and device acceptability) rather than parity alone [2,3].

Postpartum evidence highlights a trade-off between access and expulsion. Immediate postpartum LNG-IUD insertion did not compromise breastfeeding but increased expulsion [20,103,104], and placement within 48 h of vaginal birth produced expulsion rates high enough to stop a Swedish trial early, although most women resumed the method after reinsertion [105]. Placement at 2–4 weeks postpartum was noninferior to placement at 6–8 weeks for complete expulsion, although partial expulsion was slightly more frequent and did not meet the noninferiority criterion [22]. Early postpartum insertion may therefore retain much of the access advantage while avoiding the highest immediate-postpartum expulsion risk. In the immediate-versus-delayed trial, most women with an expulsion had the device reinserted, whereas almost one in five women assigned to delayed placement never returned for insertion [20], which illustrates the access advantage of immediate placement. Anchored intracesarean devices and standardized insertion techniques may further reduce expulsion or displacement in selected settings [11,111], whereas a dedicated inserter, manual rather than forceps insertion, or a modified copper shape made little difference [108,110,112]. Postpartum performance therefore depends on insertion timing, route of delivery, technique, retention mechanism, and uterine involution more than on copper versus LNG composition [19,21,96,106].

For rare complications, the major advance was precision in risk estimation. EURAS-IUD and APEX-IUD, each following very large populations, showed perforation to be uncommon and identified postpartum timing and breastfeeding as important modifiers [18,19,94,95], and APEX-IUD added heavy menstrual bleeding, higher body mass index, young age, and high parity as risk factors for expulsion [97,98]. LNG-IUDs had a higher adjusted perforation hazard than copper IUDs, but the absolute difference was small, while expulsion was slightly less common with LNG devices [93]. Counseling should therefore present absolute as well as relative risk. The ectopic pregnancy signal with the 13.5 mg LNG-IUD has now been observed in four Scandinavian analyses [64,65,66,67]; absolute incidence remained low, but it is a reasonable consideration for women with risk factors for ectopic pregnancy.

Systemic safety signals were addressed by population-based cohorts rather than device trials. For breast cancer, a Swedish nationwide cohort found a small relative increase with the 52 mg LNG-IUD (HR 1.13), lower than for desogestrel-containing methods [100], and an Israeli cohort of women aged 40–50 years found a borderline excess of invasive tumors [99]; these findings are consistent with Danish nationwide analyses of hormonal contraception that the targeted search did not retrieve [136,137], and FSRH guidance was updated in 2023 to reflect this evidence [3]. For mood, the associations with anxiolytic and antidepressant use [68,69,101] extend an earlier Danish cohort study of hormonal contraception [138], and the lower risk with lower-dose devices suggests a dose-related component; all are observational and susceptible to confounding by indication. No excess meningioma risk was found with LNG-IUDs, in contrast to several systemic progestogens [102]. These outcomes should be included in standardized safety reporting for new hormonal devices.

Emergency contraception represents a potential expansion of the LNG-IUD evidence base, but it currently rests on a single trial. In the 2021 randomized noninferiority trial, the 52 mg LNG-IUD met the noninferiority criterion for pregnancy at 1 month, with one pregnancy in the LNG group and none in the copper group [24]; because pregnancy after emergency IUD placement is rare, the confidence interval was wide relative to the event rate, and its upper limit lay close to the noninferiority margin. One-year follow-up showed similar pregnancy and continuation outcomes, with more bleeding/cramping-related discontinuation among copper users [113], and secondary analyses found no pregnancies with intercourse soon after placement and high prevention of expected pregnancies [114,115,117]; because these analyses use the same participants, they do not replicate the primary result. Professional guidance is not yet aligned: the Society of Family Planning includes the 52 mg LNG-IUD as an emergency contraception option [139], whereas the 2024 CDC Selected Practice Recommendations continue to list only the copper IUD as the intrauterine emergency method [42]. The LNG-IUD is therefore a reasonable option where guidance supports it, but a second trial would strengthen confidence in this indication.

### 4.5. Expansion Beyond Contraception

The intrauterine route increasingly functions as a therapeutic platform. For heavy menstrual bleeding, a dedicated 52 mg LNG-IUD trial showed near-complete reduction of objectively measured blood loss across BMI and parity groups [25], consistent with NICE guidance recommending the LNG-IUD as first treatment in appropriate patients, including those with suspected or diagnosed adenomyosis [140]. The MIRA program showed that both the LNG-IUD and radiofrequency ablation substantially reduce bleeding, but reintervention was more common with an LNG-IUD-first strategy and remained so at long-term follow-up, although hysterectomy rates did not differ significantly [26,125]. In primary care, the ECLIPSE trial found that the early advantage of the LNG-IUD over usual medical treatment was not sustained at 5 years and that surgery rates remained low over 10 years [122,123]. This supports the LNG-IUD as a reversible, uterus-preserving first strategy while acknowledging that some patients will later require surgery.

A small pragmatic trial comparing the 52 mg LNG-IUD with combined oral contraceptives found no clear intention-to-treat advantage on the Menorrhagia Multi-Attribute Scale at 6 or 12 months, although per-protocol analysis at 6 months favored the IUD [124]. This illustrates that pragmatic effectiveness depends on uptake, adherence, and crossover, as well as biological efficacy. In atypical endometrial hyperplasia, one randomized trial reported higher and faster complete regression with the LNG-IUD than with oral megestrol acetate [27], whereas two smaller phase II trials did not show a significant difference [31,128]; for hyperplasia without atypia, relapse after stopping treatment was common regardless of regimen [127], which supports continued surveillance and, where appropriate, prolonged progestogen therapy. These findings are consistent with ACOG guidance that conservative management requires progestin therapy and close surveillance after exclusion of concurrent carcinoma [29]. In early endometrial cancer, the evidence remains exploratory [129,130].

Evidence for adenomyosis is less robust. The LNG-IUD and dienogest both improved pain, with greater sustained pain reduction and control of uterine volume growth in the dienogest group [28], but allocation was by odd/even visit date rather than true randomization, and missing data were handled suboptimally. The study therefore supports therapeutic activity rather than definitive comparative superiority. Treatment of niche-related postmenstrual spotting is a newer indication, supported by one single-center trial with 3-year follow-up [131,132]. At an earlier developmental stage, a copper IUD releasing ulipristal acetate produced dose-related reductions in bleeding-only days in a 29-participant proof-of-concept trial, but short follow-up and endometrial changes at the highest dose preclude clinical extrapolation [32], and an LNG-IUD with an indomethacin reservoir reduced early bleeding and spotting without altering LNG release [133,134]. Their importance is conceptual: future intrauterine devices may deliver agents beyond LNG, expanding the IUD from a contraceptive device to a site-specific drug delivery platform.

Two established noncontraceptive uses of the 52 mg LNG-IUD were not mapped because they did not involve a new device or indication during the review period. The LNG-IUD provides endometrial protection during menopausal estrogen therapy, and FSRH guidance addresses its duration of use for this purpose [3,43]; it is also a recognized treatment option for endometriosis-associated pain [44]. Separately, observational studies have associated IUD use with a lower incidence of cervical cancer [39]; because this association derives from epidemiological data rather than a therapeutic application, it was treated as context rather than as part of Objective 8.

### 4.6. Evidence Maturity and Future Directions

The evidence can be grouped by maturity (Section 3.7). The strongest advances are supported by randomized comparisons or very large independent cohorts: modern perforation and expulsion estimates and treatment of heavy menstrual bleeding [19,25,26,93,96,122,125]. Eight-year use of 52 mg LNG-IUDs is supported by two independent single-arm programs and a 7-year randomized comparison [8,9,76]. Smaller copper devices, alternative postpartum timing, and 52 mg LNG-IUD emergency contraception have persuasive but narrower evidence, each resting on one or a few trials [10,22,24,89,91,113], and dose-related ectopic and mood associations are consistent across national data sources but are observational [64,65,68]. Years 9–10 of LNG-IUD use, Cu 175 mm^2^ nitinol devices, broad frameless use, alloy additions, intrauterine ball designs, cavity-measurement-guided selection, early endometrial cancer applications, and non-LNG local drug delivery remain developmental [11,12,13,15,16,17,32,81,82,83,84,129,133]. Future trials should prioritize direct long-term comparisons, patient-relevant outcomes, and prospective tests of uterine fit algorithms rather than technological novelty alone.

Outcome standardization is equally important. Studies used different pregnancy metrics, expulsion definitions, bleeding instruments, and approaches to pain, satisfaction, and continuation, limiting quantitative synthesis. A core IUD outcome set should include pregnancy, complete/partial expulsion, malposition, perforation, bleeding, dysmenorrhea, insertion pain, continuation, reasons for discontinuation, and satisfaction.

### 4.7. Limitations

Several limitations should be considered when interpreting this review. By design, this is a scoping review rather than a systematic review with meta-analysis: the objective was to map how intrauterine contraceptive technology has evolved and to characterize the breadth and maturity of the supporting evidence, not to generate a single pooled effect estimate [33,34,35,38]. This non-pooled approach was chosen because the included studies range from a 21-participant device series to a nationwide register cohort of more than two million women, and treating such disparate sources as statistically exchangeable would have produced a misleadingly tidy summary. Table 1, Table 2 and Table 3 and the thematic synthesis therefore report each study’s own published findings, and the wide variation in study size, design, and methodological rigor documented in the design-specific JBI appraisal (Supplementary Table S1) is itself a substantive finding of this review. Consistent with this approach, no formal sensitivity analysis excluding reports with major appraisal concerns was performed.

This review is a targeted map. The search was designed to retrieve the major device programs, randomized trials, and large surveillance cohorts rather than all of the IUD literature, and smaller single- or two-center observational studies outside these categories (103 records at title/abstract screening) were excluded by design, so signals from such studies, for example, on specific populations or insertion practices, may be under-represented. The scope criterion was introduced during revision, after the original corpus was known, and is declared as a protocol deviation (Supplementary Table S7); the original forty-two reports were retained, two of which would not have met the criterion. Scopus and Web of Science Core Collection added no unique records to the PubMed/MEDLINE results, which retrieved all 42 originally included reports; Embase, CENTRAL, the gray literature, and conference proceedings were not searched. The search was restricted to peer-reviewed articles in English and to journals indexed in SCIE: non-English records (Supplementary Table S6) could not be included, and the SCIE rule excluded seven otherwise eligible reports for bibliographic rather than methodological reasons, two of which are summarized in Supplementary Table S4. Finally, procedural interventions to reduce insertion pain, a prominent recent topic, were outside the scope of this device-focused review and have been synthesized elsewhere [40,41].

Several publications represent repeated follow-up of the same underlying trial or cohort (ACCESS IUS, APEX-IUD, EURAS-IUD, the emergency contraception trial, the Kyleena Satisfaction Study, ECLIPSE, MIRA, NTCu380-Mini, EURAS-LCS12, the Cu 175 mm^2^ program, and national registers in Denmark, Sweden and France); although cohort overlap was charted explicitly and participant counts were not summed across such publications, repeated reporting from successful programs can still overstate the apparent size of the independent evidence base relative to less-studied devices. Primary studies frequently had an unavoidable lack of blinding given visibly different devices, single-arm or secondary study designs, attrition during long-term follow-up, small sample sizes, or short follow-up; the 2026 EURAS-LCS12 alloy analysis was a short report with limited methodological detail, and the included adenomyosis trial used an allocation procedure based on odd or even visit dates rather than a concealed random sequence. Several modern device development programs were manufacturer-sponsored or involved investigators with industry relationships, which warrants caution when interpreting outcomes that depend on subjective assessment; independent replication remains desirable. Finally, design-specific JBI critical appraisal identifies study-level methodological strengths and weaknesses but is not a formal certainty-of-evidence framework such as GRADE.

### 4.8. Overall Interpretation

Overall, the most meaningful progress from 2016 to 2026 was not the appearance of a single ‘next-generation’ IUD, but a shift toward longer-lasting, more diverse, better characterized, and more patient-centered intrauterine care. Hormonal systems gained validated duration and dose options; copper development increasingly focused on size and mechanical compatibility; rare risks became quantifiable at population scale; postpartum, adolescent, and nulliparous use became more firmly supported; the 52 mg LNG-IUD gained randomized evidence for emergency contraception; and local intrauterine therapy expanded in heavy menstrual bleeding and endometrial disease [8,9,10,19,23,24,25,27]. The decade also cautions against equating novelty with benefit: smaller copper devices produced measurable advantages, whereas spherical geometry and precious metal alloying did not consistently do so, and promising nitinol or drug delivery platforms still require direct long-term validation [12,13,16,17,32,89]. The next major advance is therefore likely to be a device that demonstrates superior patient-relevant outcomes (reliable contraception, acceptable bleeding, less pain, secure retention, simple insertion, and sustained satisfaction) rather than one that is simply newer.

## 5. Conclusions

Between 2016 and 2026, intrauterine contraception advanced primarily through refinement rather than replacement of the conventional T-shaped device; the flexible nitinol-frame low-dose copper device comes closest to a new design, but it remains T-shaped, its phase 3 evidence is single-arm, and it appears less effective than TCu380A. The strongest evidence supports extension of 52 mg LNG-IUD contraception to 8 years, broader use in adolescents and postpartum patients, improved characterization of rare local and systemic safety events, and expansion of intrauterine therapy for heavy menstrual bleeding and selected endometrial disease; the 52 mg LNG-IUD has also emerged as an emergency contraception option, although on the basis of a single randomized trial. Lower-dose LNG-IUDs involve trade-offs, with easier placement and less antidepressant use but higher ectopic pregnancy rates. Among copper devices, smaller or size-matched designs showed the clearest patient-centered benefits, whereas spherical geometry and silver/gold alloying did not demonstrate consistent clinical superiority. Evidence beyond 8 years of LNG-IUD use, measurement-guided device matching, long-term comparative outcomes for new nitinol-frame copper devices, early endometrial cancer, and non-LNG local drug delivery platforms remains insufficient. Future innovation should therefore be judged by direct, long-term improvements in effectiveness, retention, bleeding, pain, safety, and patient satisfaction rather than by design novelty alone.

## Figures and Tables

**Figure 1:**
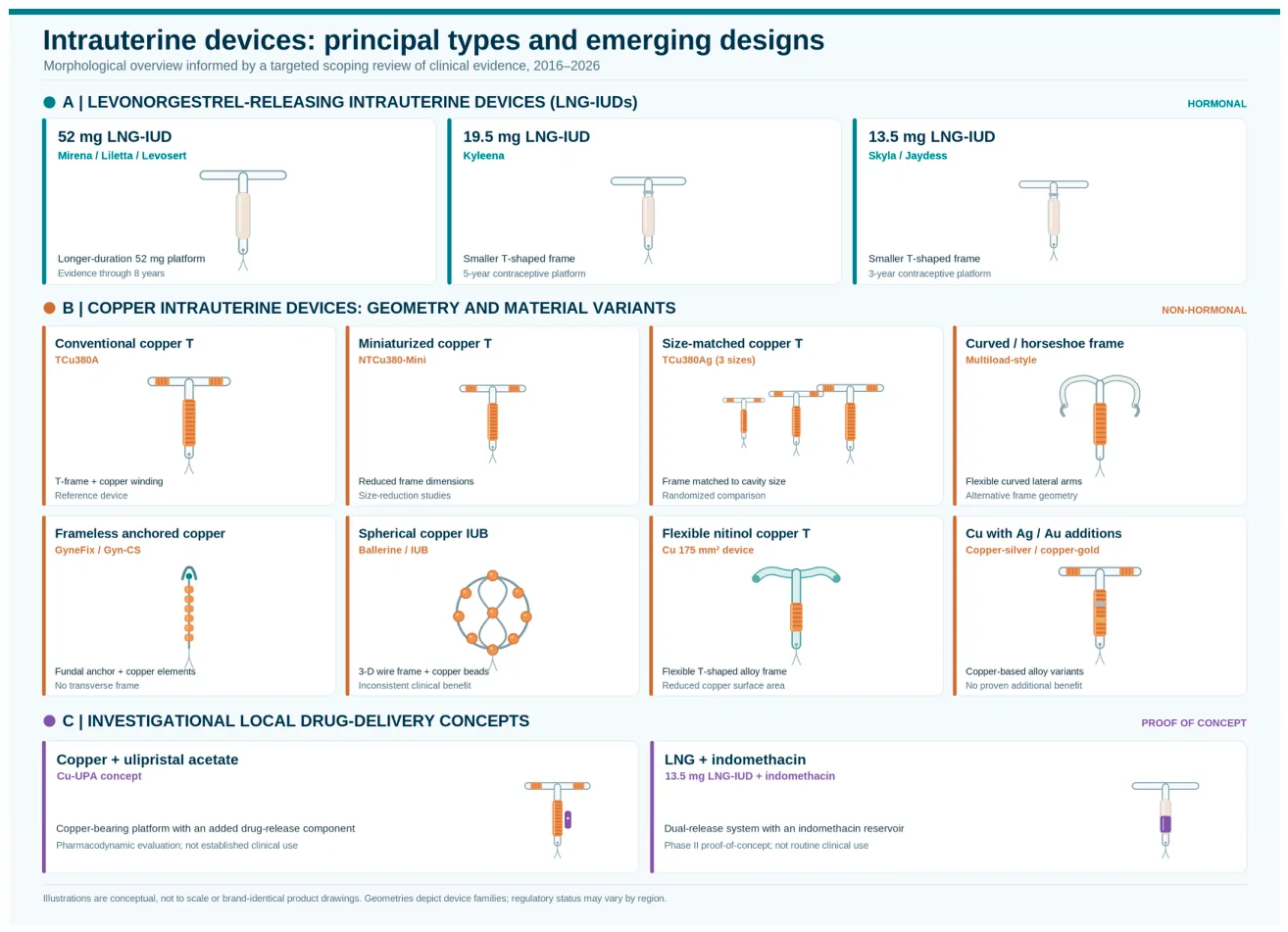
Principal types of intrauterine devices and emerging designs covered by this review. (**A**) Levonorgestrel-releasing intrauterine devices (52, 19.5, and 13.5 mg). (**B**) Copper intrauterine devices: conventional, miniaturized, and size-matched T-frames, curved, frameless anchored, spherical, flexible nitinol-frame, and silver- or gold-containing designs. (**C**) Investigational local drug delivery concepts (copper with ulipristal acetate; LNG with indomethacin). Illustrations are conceptual and not to scale.

**Figure 2:**
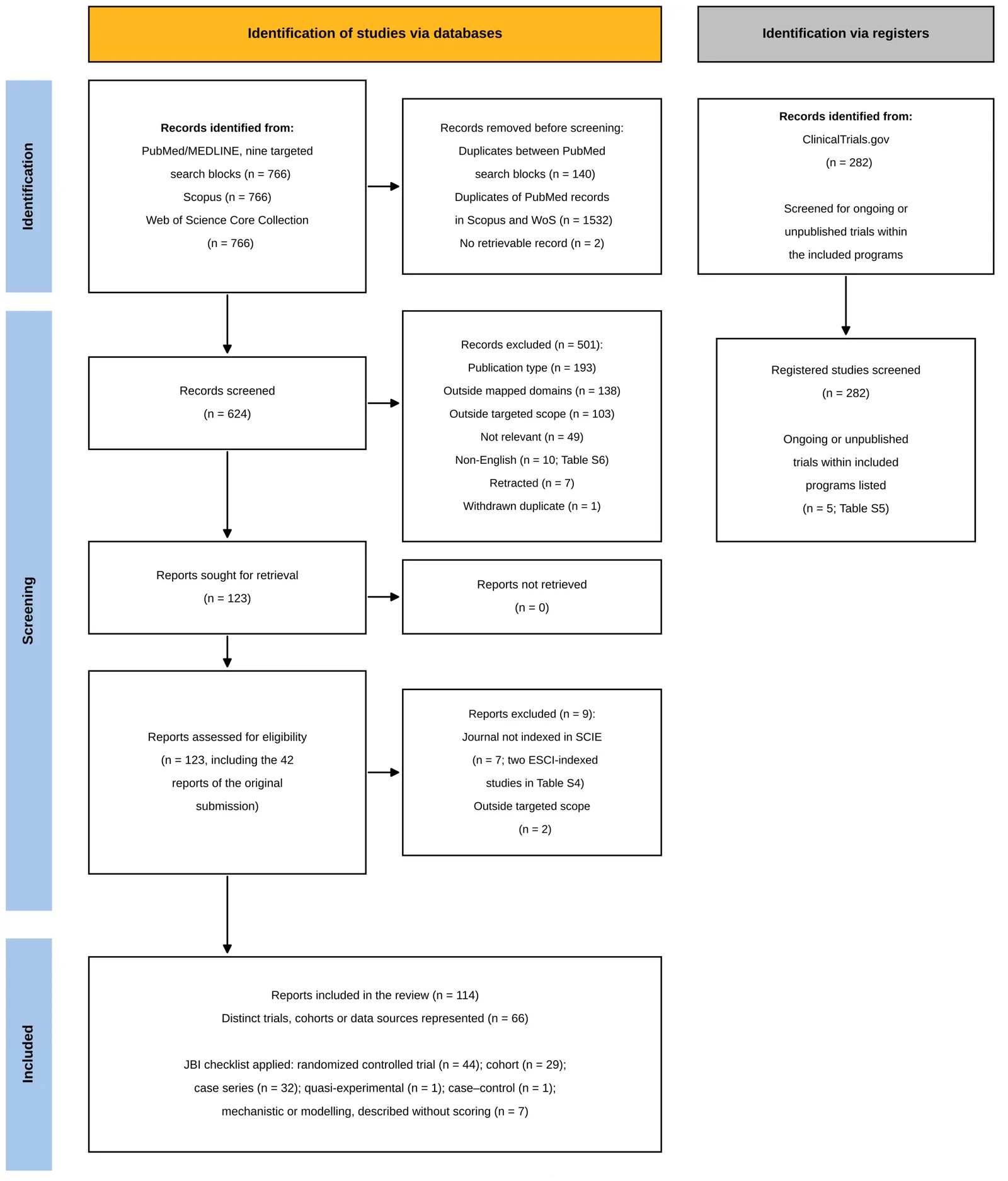
PRISMA 2020 flow diagram of the targeted search and study selection process, showing records per source and reasons for exclusion at each stage.

**Figure 3:**
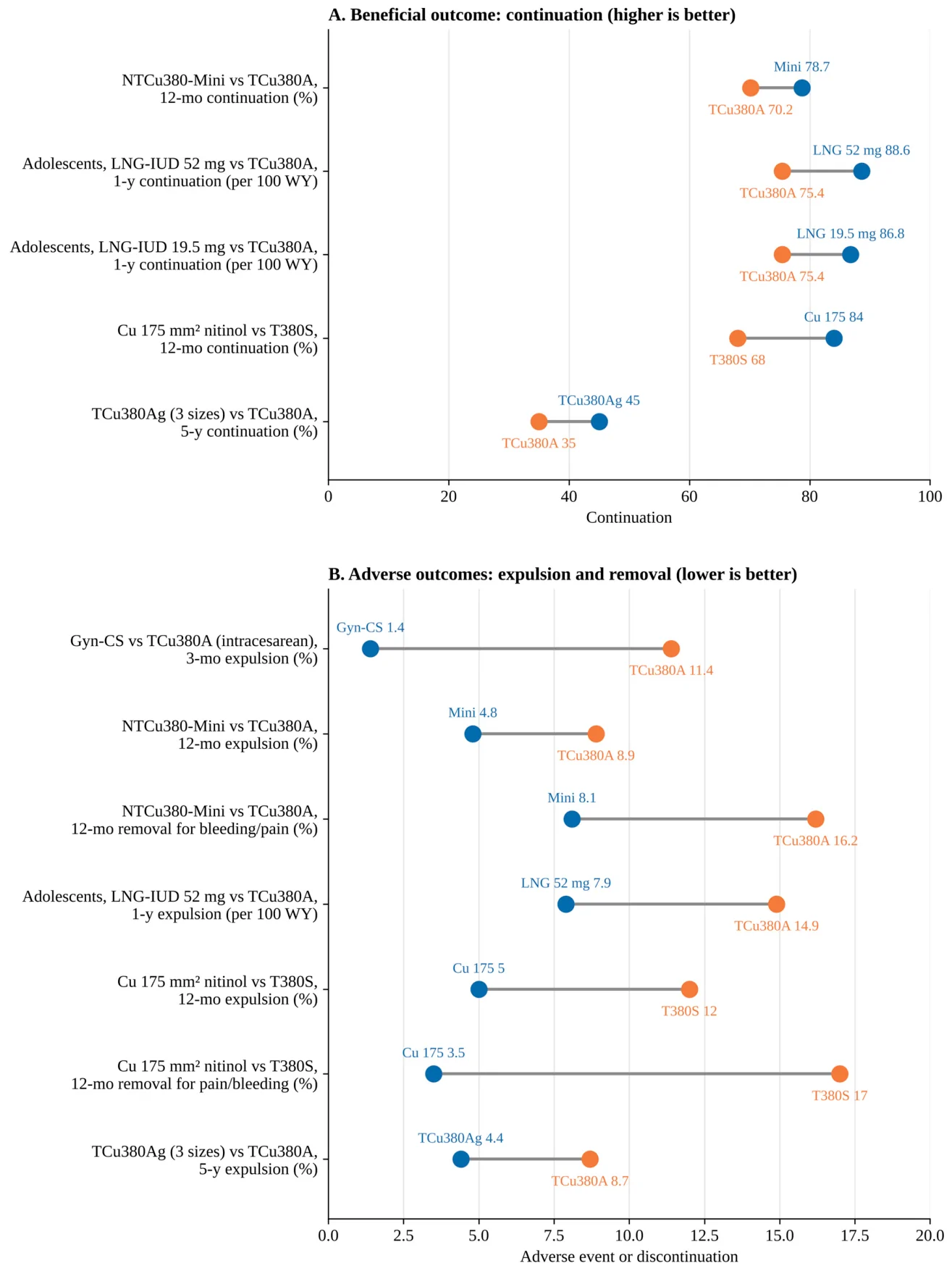
Selected randomized comparisons of device performance, shown in separate panels for beneficial and adverse outcomes. (**A**) Continuation (higher is better). (**B**) Expulsion and removal for bleeding or pain (lower is better). Values are as published: percentages for NTCu380-Mini [10], Gyn-CS [11], Cu 175 mm^2^ versus T380S [14], and TCu380Ag versus TCu380A [91]; rates per 100 woman-years (WY) at 1 year for the adolescent trial [23].

**Figure 4:**
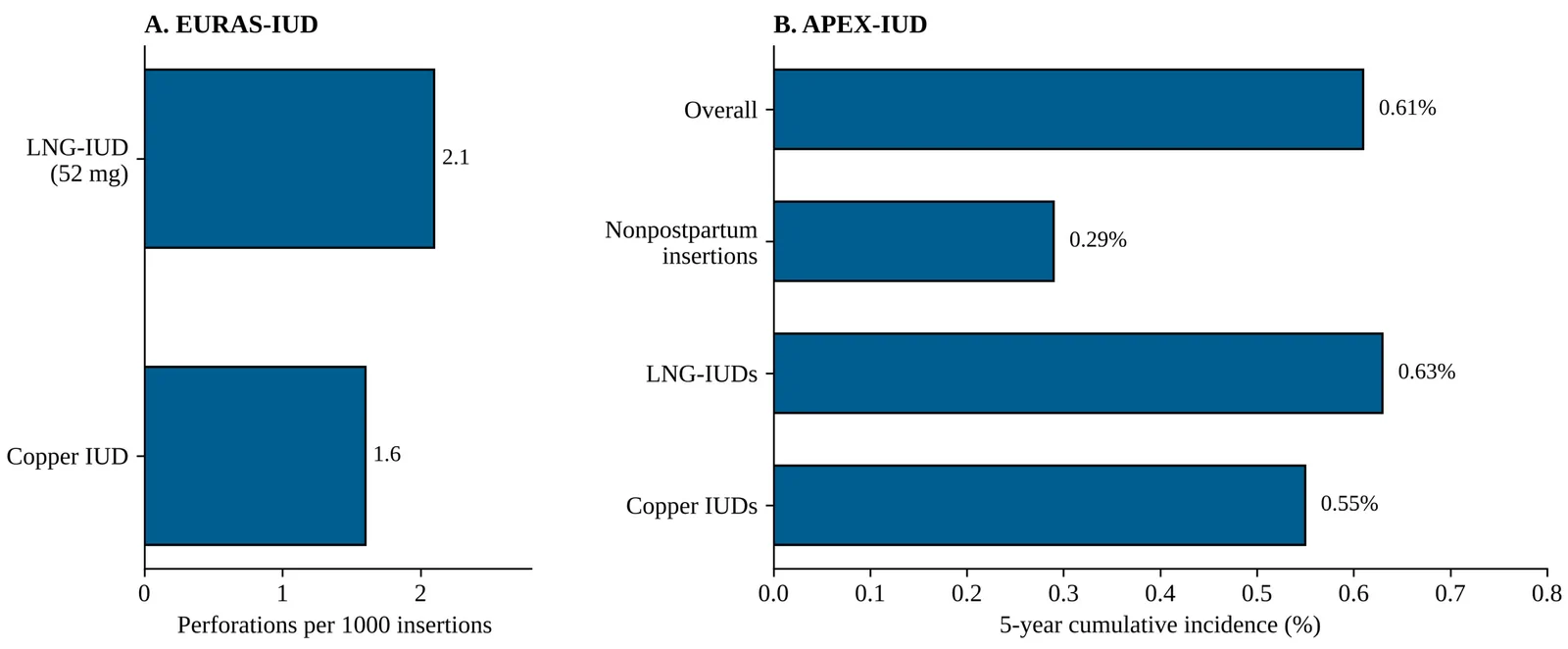
Uterine perforation in two large surveillance programs, shown on separate axes because the measures differ. (**A**) EURAS-IUD: perforations per 1000 insertions during up to 5 years of follow-up [18]. (**B**) APEX-IUD: 5-year cumulative incidence (%), overall and for nonpostpartum insertions [19] and by device type [93]. Relative-risk estimates were larger than the corresponding absolute differences.

**Table 1:** Evidence supporting extended use of 52 mg LNG-IUDs. Measures differ in denominator and time origin and are not directly comparable: WHO and ACCESS IUS rates are cumulative from insertion, whereas Mirena Extension Trial and EPIC figures cover only the years after year 5, in women who had already completed 5 years of use. Serial ACCESS IUS reports describe one cohort.

Program	Follow-Up Window	Measure (Denominator, Time Origin)	Key Finding	Design/Interpretation
WHO RCT [76]	Years 0–7	Cumulative pregnancies per 100 women, from insertion	0.53 (LNG-IUD) vs. 2.45 (TCu380A)	Randomized head-to-head comparison
ACCESS IUS, Liletta [8,79,80]	Years 0–5, 0–6, and 0–8	Cumulative life-table pregnancy (%), from insertion	0.92% (5 y); 0.87% (6 y); 1.32% (8 y); none in year 8	Single-arm phase 3; serial reports of one cohort
Mirena Extension Trial [9]	Years 6–8 only	Pearl Index and cumulative failure in years 6–8, among women who completed 5 years	Pearl Index: 0.28; cumulative failure: 0.68%	Single-arm extension; conditional on 5-year continuation
EPIC cohort [78]	Years 6–7 only	Failures per 100 woman-years in years 6 and 7, among women continuing beyond 5 years	0.25 (year 6); 0.43 (year 7)	Prospective single-group cohort; conditional on 5-year continuation
ACCESS IUS beyond 8 years [81]	Years 9–10	Number of pregnancies	None; 83 users reached year 9, and 77 reached year 10	Exploratory; small numbers
Residual LNG [82]	Beyond 8 years	Residual LNG per removed device (mg)	Median ≈ 11.2 mg at 10 years	Mechanistic; not efficacy evidence

**Table 2:** Principal copper-device innovations and the maturity of their clinical evidence.

Technology	Design Concept	Main Quantitative Signal	Evidence Interpretation
IUB/ball designs [12,13,83,84]	Spherical/3D geometry	Pilot expulsion 27%; later observational 3.4–5.3%; EURAS-LCS12 IUB aHR 3.6 vs. Tatum-T	Inconsistent; no clear superiority
Anchored frameless, postpartum [11,86,87,88]	Fundal anchoring at cesarean delivery	RCT: lower 3 mo expulsion than TCu380A (Figure 3); series 0–1%; GyneFix cohort 7.6% at 12 months	Promising postpartum signal; one small RCT
NTCu380-Mini [10,89,90]	Smaller Nova-T copper frame	Higher continuation; lower bleeding/pain removal and expulsion; expulsion with menstrual cup use	Best randomized size reduction evidence; pregnancy outcomes outside SCIE (Table S4)
Size-matched TCu380Ag [91]	Three sizes matched to uterocervical length	Higher 5 y continuation and fewer expulsions than TCu380A (Figure 3); no pregnancies	Single randomized trial
Cu 175 mm^2^ nitinol [14,15,16,92]	Flexible nitinol frame, reduced copper	Phase 2 RCT vs. T380S: better continuation (Figure 3); phase 3: 5 y pregnancy 4.1%, expulsion 4.7%	Better tolerability, lower efficacy than expected with TCu380A; phase 3 single-arm
Copper load and frame [85]	Surface area, frame shape, arm flexibility	Higher 1 y continuation with <300 mm^2^, horseshoe, and flexible-arm devices (users < 30 y)	Observational; hypothesis-generating
Ag/Au alloys [17]	Copper–silver/gold	Pregnancy/PID similar; more technical complaints with Au	No demonstrated clinical benefit

**Table 3:** Condensed evidence for therapeutic and local intrauterine drug delivery applications.

Clinical Use	Intervention/Comparator	Key Quantitative Result	Interpretation
HMB, ACCESS subgroup [119]	52 mg LNG-IUD	No HMB in 74.7% (cycle 1) and 92.1% (cycle 6)	Self-reported subgroup of a contraceptive trial
Objective HMB trial [25]	52 mg LNG-IUD	Median blood loss reduction of 93.3% by cycle 3	Single-arm treatment trial
HMB vs. oral agents [120]	LNG-IUD vs. norethisterone vs. tranexamic acid	6 mo blood loss reduction of 85.8% vs. 53.1% vs. 60.8%	Small RCT (62 analyzed)
Second 52 mg product [121]	Levosert vs. Mirena	6 mo blood loss change of −130 vs. −127 mL; noninferior	Supports interchangeable 52 mg products
ECLIPSE [122,123]	LNG-IUD vs. usual medical treatment	MMAS difference of 3.9 (NS) at 5 y; surgery in 29% by 10 y	Early advantage not sustained; low surgery rates
COC comparison [124]	LNG-IUD vs. COC	No ITT difference at 6 or 12 mo	Small pragmatic RCT
MIRA [26,125,126]	LNG-IUD vs. ablation	Reintervention of 27% vs. 10% at 24 mo; 35.3% vs. 19.1% in the long term; preference cohort: 35% vs. 14%	More reintervention with LNG first
Non-atypical hyperplasia [30,127]	LNG-IUD vs. vaginal progesterone or oral MPA	Regression of 95.8% vs. 90.8%; relapse of 41% within 24 mo after stopping	Similar efficacy; surveillance needed
Atypical hyperplasia [27,31,128]	LNG-IUD vs. megestrol	12 mo regression of 91.9% vs. 77.0% in one RCT; NS in two phase II RCTs	Favorable but inconsistent
Early endometrial cancer [129,130]	LNG-IUD ± metformin/weight loss; megestrol ± LNG-IUD	6 mo pCR of 43% (adenocarcinoma); no gain from adding LNG-IUD to megestrol	Exploratory
Cesarean niche [131,132]	LNG-IUD vs. hysteroscopic resection	≥50% spotting reduction: 78.4% vs. 73.1% (6 mo); 98.9% vs. 51.7% (36 mo)	Single-center RCT; superior in the long term
Adenomyosis [28]	LNG-IUD vs. dienogest	Both improved pain; 8/48 LNG-IUD expulsions	Pseudo-random allocation
Cu-UPA [32]	Copper IUD releasing UPA at 5/20/40 µg/d	Bleeding-only days: −16.7%, −40.5%, and −77%	Proof-of-concept
Indomethacin–LNG [133,134]	13.5 mg LNG-IUD with indomethacin	Bleeding/spotting days: −32% (low dose, first 90 days)	Proof-of-concept

## Data Availability

All data supporting the findings of this scoping review are derived from the published sources cited in the manuscript. The study-level evidence map, design-specific critical appraisal, and full reproducible search strategies are provided in the Supplementary Materials. No new participant-level dataset was generated.

## Supplementary Materials

The following supporting information can be downloaded at: https://www.mdpi.com/article/10.3390/jcm15218338/s1. The following supporting information accompanies the manuscript: Table S1: Design-to-tool mapping and JBI critical appraisal of the included reports [141,142,143,144]; Table S2: Characteristics and evidence map of the included publications; Table S3: Full reproducible electronic search strategies, search dates, and records retrieved per source; Table S4: Studies excluded under the SCIE eligibility rule; Table S5: Ongoing trials identified during the search; Table S6: Non-English, unretrievable, and withdrawn records; Table S7: Protocol elements fixed before the search, post hoc refinements, and deviations; PRISMA-ScR Checklist: completed reporting checklist. The PRISMA flow diagram is presented in the main manuscript.

## References

[1] World Health Organization (2026). Intrauterine Devices (IUDs). https://www.who.int/news-room/fact-sheets/detail/intrauterine-devices.

[2] Nguyen A.T., Curtis K.M., Tepper N.K., Kortsmit K., Brittain A.W., Snyder E.M., Cohen M.A., Zapata L.B., Whiteman M.K. (2024). U.S. Medical Eligibility Criteria for Contraceptive Use, 2024. MMWR Recomm. Rep..

[3] Faculty of Sexual & Reproductive Healthcare (2023). FSRH Clinical Guideline: Intrauterine Contraception (March 2023, Amended).

[4] ESHRE Capri Workshop Group (2008). Intrauterine devices and intrauterine systems. Hum. Reprod. Update.

[5] Wu J.P., Pickle S. (2014). Extended use of the intrauterine device: A literature review and recommendations for clinical practice. Contraception.

[6] Heinemann K., Reed S., Moehner S., Minh T.D. (2015). Comparative contraceptive effectiveness of levonorgestrel-releasing and copper intrauterine devices: The European Active Surveillance Study for Intrauterine Devices. Contraception.

[7] Liang H., Li L., Yuan W., Zou Y., Gao E., Duffy J., Wu S. (2014). Dimensions of the endometrial cavity and intrauterine device expulsion or removal for displacement: A nested case-control study. BJOG.

[8] Creinin M.D., Jensen J.T. (2023). Extending use of levonorgestrel 52 mg intrauterine device to 8 years. Am. J. Obstet. Gynecol..

[9] Jensen J.T., Lukkari-Lax E., Schulze A., Wahdan Y., Serrani M., Kroll R. (2022). Contraceptive efficacy and safety of the 52-mg levonorgestrel intrauterine system for up to 8 years: Findings from the Mirena Extension Trial. Am. J. Obstet. Gynecol..

[10] Hubacher D., Schreiber C.A., Turok D.K., Jensen J.T., Creinin M.D., Nanda K., White K.O., Dayananda I., Teal S.B., Chen P.-L. (2022). Continuation rates of two different-sized copper intrauterine devices among nulliparous women: Interim 12-month results of a single-blind, randomised, multicentre trial. eClinicalMedicine.

[11] Unal C., Eser A., Tozkir E., Wildemeersch D. (2018). Comparison of expulsions following intracesarean placement of an innovative frameless copper-releasing IUD (Gyn-CS) versus the TCu380A: A randomized trial. Contraception.

[12] Boehnke T., Bauerfeind A., Eggebrecht L., Cellier C., Lange J.A., Heinemann K., Madden T. (2024). Does the shape of the copper intrauterine device play a role in expulsion? Results from the ongoing European Active Surveillance Study on LCS12. Contraception.

[13] Wiebe E., Trussell J. (2016). Discontinuation rates and acceptability during 1 year of using the intrauterine ball (the SCu380A). Contraception.

[14] Reeves M.F., Katz B.H., Canela J.M., Hathaway M.J., Tal M.G. (2017). A randomized comparison of a novel nitinol-frame low-dose-copper intrauterine contraceptive and a copper T380S intrauterine contraceptive. Contraception.

[15] Creinin M.D., Gawron L.M., Roe A.H., Blumenthal P.D., Boraas C.M., Hou M.Y., McNicholas C., Schreifels M.J., Peters K., Culwell K. (2025). Three-year efficacy, safety, and tolerability outcomes from a phase 3 study of a low-dose copper intrauterine device. Contraception.

[16] Gawron L.M., Roe A.H., Nelson A.L., Bernard C., Castaño P.M., Cwiak C., Schreifels M.J.B., Peters K., Culwell K.R., Gray E.B. (2026). Five-Year Phase 3 Efficacy and Safety Outcomes With a Low-Dose Copper Intrauterine Device. Obstet. Gynecol..

[17] Boehnke T., Eggebrecht L., Rizzo M., Klinghardt M., Heinemann K. (2026). Polished but pointless? Assessing the potential of gold and silver-alloy IUDs versus copper-only devices in a large multinational cohort (EURAS-LCS12). Eur. J. Contracept. Reprod. Health Care.

[18] Barnett C., Moehner S., Minh T.D., Heinemann K. (2017). Perforation risk and intra-uterine devices: Results of the EURAS-IUD 5-year extension study. Eur. J. Contracept. Reprod. Health Care.

[19] Anthony M.S., Reed S.D., Armstrong M.A., Getahun D., Gatz J.L., Saltus C.W., Zhou X., Schoendorf J., Postlethwaite D.A., Raine-Bennett T. (2021). Design of the Association of Uterine Perforation and Expulsion of Intrauterine Device study: A multisite retrospective cohort study. Am. J. Obstet. Gynecol..

[20] Turok D.K., Leeman L., Sanders J.N., Thaxton L., Eggebroten J.L., Yonke N., Bullock H., Singh R., Gawron L.M., Espey E. (2017). Immediate postpartum levonorgestrel intrauterine device insertion and breast-feeding outcomes: A noninferiority randomized controlled trial. Am. J. Obstet. Gynecol..

[21] Laporte M., Marangoni M., Surita F., Juliato C.T., Miadaira M., Bahamondes L. (2020). Postplacental placement of intrauterine devices: A randomized clinical trial. Contraception.

[22] Averbach S., Kully G., Hinz E., Dey A., Berkley H., Hildebrand M., Vaida F., Haider S., Hofler L.G. (2023). Early vs interval postpartum intrauterine device placement: A randomized clinical trial. JAMA.

[23] Anjos F.C.Q.S., Marcelino A.C., Espejo-Arce X., Pereira P.d.C., Barbosa P.F., Juliato C.T., Bahamondes L. (2024). Clinical Assessment of 3 Intrauterine Devices in Adolescent Girls: A Randomized Clinical Trial. J. Pediatr. Adolesc. Gynecol..

[24] Turok D.K., Gero A., Simmons R.G., Kaiser J.E., Stoddard G.J., Sexsmith C.D., Gawron L.M., Sanders J.N. (2021). Levonorgestrel vs. copper intrauterine devices for emergency contraception. N. Engl. J. Med..

[25] Creinin M.D., Barnhart K.T., Gawron L.M., Eisenberg D., Mabey R.G., Jensen J.T. (2023). Heavy Menstrual Bleeding Treatment With a Levonorgestrel 52-mg Intrauterine Device. Obstet. Gynecol..

[26] Beelen P., van den Brink M.J., Herman M.C., Geomini P.M., Dekker J.H., Duijnhoven R.G., Mak N., van Meurs H.S., Coppus S.F., van der Steeg J.W. (2021). Levonorgestrel-releasing intrauterine system versus endometrial ablation for heavy menstrual bleeding. Am. J. Obstet. Gynecol..

[27] Alnemr A.A., Harb O.A., Atia H. (2024). The efficacy of the levonorgestrel intrauterine system versus oral megestrol acetate in treating atypical endometrial hyperplasia: A superior randomized controlled trial. J. Gynecol. Oncol..

[28] Guo W., Lin Y., Hu S., Shen Y. (2023). Compare the Efficacy of Dienogest and the Levonorgestrel Intrauterine System in Women with Adenomyosis. Clin. Ther..

[29] American College of Obstetricians and Gynecologists (2023). Management of Endometrial Intraepithelial Neoplasia or Atypical Endometrial Hyperplasia: ACOG Clinical Consensus No. 5. Obstet. Gynecol..

[30] Gezer Ş., Köle E., Aksoy L. (2023). Vaginal micronized progesterone versus the levonorgestrel-releasing intrauterine system for treatment of non-atypical endometrial hyperplasia: A randomized controlled trial. Int. J. Gynaecol. Obstet..

[31] Xu Z., Yang B., Shan W., Liao J., Shao W., Wu P., Zhou S., Ning C., Luo X., Zhu Q. (2023). Comparison of the effect of levonorgestrel-intrauterine system with or without oral megestrol acetate on fertility-preserving treatment in patients with atypical endometrial hyperplasia: A prospective, open-label, randomized controlled phase II study. Gynecol. Oncol..

[32] Brache V., Vieira C.S., Plagianos M., Lansiaux M., Merkatz R., Sussman H., Cochon L., Tejada A.S., Kumar N., Loeven D. (2021). Pharmacodynamics and pharmacokinetics of a copper intrauterine contraceptive system releasing ulipristal acetate: A randomized proof-of-concept study. Contraception.

[33] Arksey H., O’Malley L. (2005). Scoping studies: Towards a methodological framework. Int. J. Soc. Res. Methodol..

[34] Levac D., Colquhoun H., O’Brien K.K. (2010). Scoping studies: Advancing the methodology. Implement Sci..

[35] Aromataris E., Lockwood C., Porritt K., Pilla B., Jordan Z. (2024). JBI Manual for Evidence Synthesis.

[36] Tricco A.C., Lillie E., Zarin W., O’Brien K.K., Colquhoun H., Levac D., Moher D., Peters M.D.J., Horsley T., Weeks L. (2018). PRISMA extension for scoping reviews (PRISMA-ScR): Checklist and explanation. Ann. Intern Med..

[37] Page M.J., McKenzie J.E., Bossuyt P.M., Boutron I., Hoffmann T.C., Mulrow C.D., Shamseer L., Tetzlaff J.M., Akl E.A., Brennan S.E. (2021). The PRISMA 2020 statement: An updated guideline for reporting systematic reviews. BMJ.

[38] Munn Z., Peters M.D.J., Stern C., Tufanaru C., McArthur A., Aromataris E. (2018). Systematic review or scoping review? Guidance for authors when choosing between a systematic or scoping review approach. BMC Med. Res. Methodol..

[39] Cortessis V.K., Barrett M., Brown Wade N., Enebish T., Perrigo J.L., Tobin J., Zhong C., Zink J., Isiaka V., Muderspach L.I. (2017). Intrauterine device use and cervical cancer risk: A systematic review and meta-analysis. Obstet. Gynecol..

[40] Lopez L.M., Bernholc A., Zeng Y., Allen R.H., Bartz D., A O’Brien P., Hubacher D. (2015). Interventions for pain with intrauterine device insertion. Cochrane Database Syst. Rev..

[41] Gemzell-Danielsson K., Jensen J.T., Monteiro I., Peers T., Rodriguez M., Sardo A.D.S., Bahamondes L. (2019). Interventions for the prevention of pain associated with the placement of intrauterine contraceptives: An updated review. Acta Obstet. Gynecol. Scand..

[42] Curtis K.M., Nguyen A.T., Tepper N.K., Zapata L.B., Snyder E.M., Hatfield-Timajchy K., Kortsmit K., Cohen M.A., Whiteman M.K. (2024). U.S. Selected Practice Recommendations for Contraceptive Use, 2024. MMWR Recomm. Rep..

[43] Depypere H., Inki P. (2015). The levonorgestrel-releasing intrauterine system for endometrial protection during estrogen replacement therapy: A clinical review. Climacteric.

[44] Becker C.M., Bokor A., Heikinheimo O., Horne A., Jansen F., Kiesel L., King K., Kvaskoff M., Nap A., Petersen K. (2022). ESHRE guideline: Endometriosis. Hum. Reprod. Open.

[45] Creinin M.D., Jansen R., Starr R.M., Gobburu J., Gopalakrishnan M., Olariu A. (2016). Levonorgestrel release rates over 5 years with the Liletta 52-mg intrauterine system. Contraception.

[46] Jensen J.T., Reinecke I., Post T.M., Lukkari-Lax E., Hofmann B.M. (2023). Extended use of levonorgestrel-releasing intrauterine system (LNG-IUS) 52 mg: A population pharmacokinetic approach to estimate in vivo levonorgestrel release rates and systemic exposure including comparison with two other LNG-IUSs. Contraception.

[47] Hofmann B.M., Apter D., Bitzer J., Reinecke I., Serrani M., Höchel J., Merz M. (2020). Comparative pharmacokinetic analysis of levonorgestrel-releasing intrauterine systems and levonorgestrel-containing contraceptives with oral or subdermal administration route. Eur. J. Contracept. Reprod. Health Care.

[48] Nelson A.L. (2017). Levonorgestrel-releasing intrauterine system (LNG-IUS 12) for prevention of pregnancy for up to five years. Expert Rev. Clin. Pharmacol..

[49] Beckert V., Ahlers C., Frenz A.K., Gerlinger C., Bannemerschult R., Lukkari-Lax E. (2019). Bleeding patterns with the 19.5 mg LNG-IUS, with special focus on the first year of use: Implications for counselling. Eur. J. Contracept. Reprod. Health Care.

[50] Frenz A.K., Ahlers C., Beckert V., Gerlinger C., Friede T. (2021). Predicting menstrual bleeding patterns with levonorgestrel-releasing intrauterine systems. Eur. J. Contracept. Reprod. Health Care.

[51] Beckert V., Aqua K., Bechtel C., Cornago S., Kallner H.K., Schulze A., Parashar P., Waddington A., Donders G. (2020). Insertion experience of women and health care professionals in the Kyleena® Satisfaction Study. Eur. J. Contracept. Reprod. Health Care.

[52] Donders G., Kopp Kallner H., Hauck B., Bauerfeind A., Frenz A.-K., Zvolanek M., Stovall D.W. (2023). Bleeding profile satisfaction and pain and ease of placement with levonorgestrel 19.5 mg IUD: Findings from the Kyleena Satisfaction study. Eur. J. Contracept. Reprod. Health Care.

[53] Stovall D.W., Aqua K., Römer T., Donders G., Sørdal T., Hauck B., de la Llata E.S., Kallner H.K., Salomon J., Zvolanek M. (2021). Satisfaction and continuation with LNG-IUS 12: Findings from the real-world Kyleena Satisfaction study. Eur. J. Contracept. Reprod. Health Care.

[54] Römer T., Frenz A.K., Dietrich-Ott S., Fiedler A. (2024). The use of LNG-IUS-19.5 mg in daily gynecological routine practice in Germany: Data from the Kyleena™ Satisfaction Study (KYSS). Arch. Gynecol. Obstet..

[55] Gemzell-Danielsson K., Buhling K.J., Dermout S.M., Lukkari-Lax E., Montegriffo E., Apter D. (2016). A Phase III, single-arm study of LNG-IUS 8, a low-dose levonorgestrel intrauterine contraceptive system (total content 13.5mg) in postmenarcheal adolescents. Contraception.

[56] Fan G., Kang S., Ren M., Weisberg E., Lukkari-Lax E., Roth K., Shin S. (2017). A single-arm phase III study exploring the efficacy and safety of LNG-IUS 8, a low-dose levonorgestrel intrauterine contraceptive system (total content 13.5 mg), in an Asia-Pacific population. Contraception.

[57] Gemzell-Danielsson K., Apter D., Lukkari-Lax E., Roth K., Serrani M. (2017). Overcoming barriers to levonorgestrel-releasing intrauterine system placement: An evaluation of placement of LNG-IUS 8 using the modified EvoInserter® in a majority nulliparous population. Contraception.

[58] Apter D., Briggs P., Tuppurainen M., Grunert J., Lukkari-Lax E., Rybowski S., Gemzell-Danielsson K. (2016). A 12-month multicenter, randomized study comparing the levonorgestrel intrauterine system with the etonogestrel subdermal implant. Fertil. Steril..

[59] Borgatta L., Buhling K.J., Rybowski S., Roth K., Rosen K. (2016). A multicentre, open-label, randomised phase III study comparing a new levonorgestrel intrauterine contraceptive system (LNG-IUS 8) with combined oral contraception in young women of reproductive age. Eur. J. Contracept. Reprod. Health Care.

[60] Perello-Capo J., Estadella-Tarriel J., Gich-Saladich I., Bailón-Queiruga M., Llurba-Olivé E., Calaf-Alsina J. (2023). Bleeding profile and safety of a levonorgestrel 13.5 mg intrauterine device versus Nova T copper 380 mm^2^ intrauterine device: Results of a 3-year, single-center, randomized phase 4 study. Contraception.

[61] Perelló-Capó J., Estadella-Tarriel J., Gich-Saladich I., Bailón-Queiruga M., Llurba-Olivé E., Calaf-Alsina J. (2024). Health-related quality of life and satisfaction associated with 3-year use of a levonorgestrel 13.5 mg intrauterine device vs Nova T copper 380 mm^2^ intrauterine device: Results of a phase 4 randomized controlled trial. Contraception.

[62] Law A., Liao L., Lin J., Yaldo A., Lynen R. (2018). Twelve-month discontinuation rates of levonorgestrel intrauterine system 13.5 mg and subdermal etonogestrel implant in women aged 18–44: A retrospective claims database analysis. Contraception.

[63] Anjos F.C.Q.S., Marcelino A.C., Espejo-Arce X., Pereira P.d.C., Barbosa P.F., Juliato C.T., Bahamondes L. (2023). Pain and ease of insertion of three different intrauterine devices in Brazilian adolescents: A participant-blinded randomized trial. Contraception.

[64] Meaidi A., Torp-Pedersen C., Lidegaard O., Morch L.S. (2023). Ectopic pregnancy risk in users of levonorgestrel-releasing intrauterine systems with 52, 19.5, and 13.5 mg of hormone. JAMA.

[65] Kopp-Kallner H., Linder M., Cesta C.E., Segovia Chacón S., Kieler H., Graner S. (2022). Method of Hormonal Contraception and Protective Effects Against Ectopic Pregnancy. Obstet. Gynecol..

[66] Graner S., Mc Taggart J., Nordström F., Melander E., Widenberg J., Kopp Kallner H. (2019). Levonorgestrel intrauterine contraceptive systems (13.5 mg and 52 mg) and risk of ectopic pregnancy. Acta Obstet. Gynecol. Scand..

[67] Elgemark K., Graner S., McTaggart J., Löfström J.R., Sörensen D., Envall N.R., Kallner H.K. (2022). The 13.5-mg, 19.5-mg, and 52-mg Levonorgestrel-Releasing Intrauterine Systems and Risk of Ectopic Pregnancy. Obstet. Gynecol..

[68] Skovlund C.W., Møller A.L., Torp-Pedersen C., Mørch L.S., Meaidi A. (2024). Depression risk in users of different doses of levonorgestrel intrauterine systems: A nationwide prospective cohort study. Lancet Reg. Health Eur..

[69] Roland N., Baricault B., Weill A., Bouillon K., Dray-Spira R., Duranteau L., Zureik M. (2023). Association Between Doses of Levonorgestrel Intrauterine Systems and Subsequent Use of Psychotropic Drugs in France. JAMA.

[70] Darney P.D., Stuart G.S., Thomas M.A., Cwiak C., Olariu A., Creinin M.D. (2018). Amenorrhea rates and predictors during 1 year of levonorgestrel 52 mg intrauterine system use. Contraception.

[71] Schreiber C.A., Teal S.B., Blumenthal P.D., Keder L.M., Olariu A.I., Creinin M.D. (2018). Bleeding patterns for the Liletta® levonorgestrel 52 mg intrauterine system. Eur. J. Contracept. Reprod. Health Care.

[72] Gilliam M.L., Jensen J.T., Eisenberg D.L., Thomas M.A., Olariu A., Creinin M.D. (2021). Relationship of parity and prior cesarean delivery to levonorgestrel 52 mg intrauterine system expulsion over 6 years. Contraception.

[73] Turok D.K., Eisenberg D.L., Teal S.B., Keder L.M., Creinin M.D. (2016). A prospective assessment of pelvic infection risk following same-day sexually transmitted infection testing and levonorgestrel intrauterine system placement. Am. J. Obstet. Gynecol..

[74] Carr B.R., Thomas M.A., Gangestad A., Eisenberg D.L., Olariu A., Creinin M.D. (2021). Conception rates in women desiring pregnancy after levonorgestrel 52 mg intrauterine system (Liletta®) discontinuation. Contraception.

[75] Kerns J.L., Keder L.M., Cwiak C.A., Westhoff C.L., Creinin M.D. (2024). Hormone-related side effects in new users of a levonorgestrel 52-mg intrauterine device. Am. J. Obstet. Gynecol..

[76] Ti A.J., Roe A.H., Whitehouse K.C., Smith R.A., Gaffield M.E., Curtis K.M. (2020). Effectiveness and safety of extending intrauterine device duration: A systematic review. Am. J. Obstet. Gynecol..

[77] Bahamondes L., Fernandes A., Bahamondes M.V., Juliato C.T., Ali M., Monteiro I. (2018). Pregnancy outcomes associated with extended use of the 52-mg 20 microg/day levonorgestrel-releasing intrauterine system beyond 60 months: A chart review of 776 women in Brazil. Contraception.

[78] McNicholas C., Swor E., Wan L., Peipert J.F. (2017). Prolonged use of the etonogestrel implant and levonorgestrel intrauterine device: 2 years beyond Food and Drug Administration-approved duration. Am. J. Obstet. Gynecol..

[79] Teal S.B., Turok D.K., Chen B.A., Kimble T., Olariu A.I., Creinin M.D. (2019). Five-Year Contraceptive Efficacy and Safety of a Levonorgestrel 52-mg Intrauterine System. Obstet. Gynecol..

[80] Westhoff C.L., Keder L.M., Gangestad A., Teal S.B., Olariu A.I., Creinin M.D. (2020). Six-year contraceptive efficacy and continued safety of a levonorgestrel 52 mg intrauterine system. Contraception.

[81] Chen B.A., Kimble T., Harris L.H., Kerns J.L., Olariu A.I., Creinin M.D. (2023). Levonorgestrel 52-mg Intrauterine Device Efficacy and Safety After More Than 8 Years of Use. Obstet. Gynecol..

[82] Bahamondes L., Chinaglia K., Denkena I.L., Marcelino A.C., Pereira P.d.C., Juliato C.T., Mónica F.Z., Costa J.L. (2026). Quantification of residual levonorgestrel following extended use of the levonorgestrel 52 mg intrauterine device beyond 8 years. Contraception.

[83] Yaron M., Viviano M., Guillot C., Aharon A., Shkolnik K. (2019). Real-world experience with the IUB Ballerine MIDI copper IUD: An observational study in the French-speaking region of Switzerland. Eur. J. Contracept. Reprod. Health Care.

[84] Baram I., Aharon A., Klein R., Shkolnik K. (2020). Real-world experience with the IUB Ballerine MIDI copper IUD: An observational, single-centre study in Israel. Eur. J. Contracept. Reprod. Health Care.

[85] Akintomide H., Brima N., Mansour D.J., Shawe J. (2021). Copper IUD continuation, unwanted effects and cost consequences at 1 year in users aged under 30—A secondary analysis of the EURAS-IUD study. Eur. J. Contracept. Reprod. Health Care.

[86] Eser A., Unal C., Albayrak B., Wildemeersch D. (2018). Clinical experience with a novel anchored, frameless copper-releasing contraceptive device for intra-caesarean insertion. Eur. J. Contracept. Reprod. Health Care.

[87] Kutlucan H., Karabacak R.O., De Buyser S., Erdem A., Bozkurt N., Demirdağ E., Wildemeersch D. (2022). Assessment of a Novel Fixation Method of a Frameless Intrauterine Contraceptive Device Inserted during Cesarean Delivery as a Means of Preventing Displacements and Expulsions: A Prospective Observational Study. Life.

[88] Che Y., Hou G.F., Zhang H.P., Yang H., Lin S.-J., Gan T., Yang W.-H., Shi C.-H., Ren W.-J., Xu Y.-Q. (2023). Effectiveness, safety, and acceptability of postplacental insertion of GyneFix postpartum intrauterine device among women undergoing cesarean section: A multicenter prospective cohort study in China. Contraception.

[89] Hubacher D., Schreiber C.A., Turok D.K., Teal S.B., Nelson A.L., Jensen J.T., Chen M.J., Nanda K., White K.O., Dayananda I. (2026). Comparing the TCu380A and a small Nova-T copper IUD: Secondary analysis of discontinuation probabilities, bleeding outcomes and satisfaction in nulliparous participants from a 3-year randomized trial. Contraception.

[90] Brown J.E., Creinin M.D., Wu H., Hubacher D., Schreiber C.A., Kaneshiro B., Nanda K., Blithe D.L. (2024). Menstrual cup use and intrauterine device expulsion in a copper intrauterine device randomized trial. Contraception.

[91] Bhatla N., Sehgal R., Konar H., Vivekanand A., Kriplani A., Vanamail P., Purandare C.N. (2023). A 5-year comparative study of efficacy and acceptability of three different sizes of TCu380Ag and TCu380A intrauterine devices. Int. J. Gynaecol. Obstet..

[92] Turok D.K., Nelson A.L., Dart C., Schreiber C.A., Peters K., Schreifels M.J.B., Katz B.M. (2020). Efficacy, Safety, and Tolerability of a New Low-Dose Copper and Nitinol Intrauterine Device: Phase 2 Data to 36 Months. Obstet. Gynecol..

[93] Gatz J.L., Armstrong M.A., Postlethwaite D., Raine-Bennett T., Chillemi G., Alabaster A., Merchant M., Reed S.D., Ichikawa L., Getahun D. (2022). Association between intrauterine device type and risk of perforation and device expulsion: Results from the Association of Perforation and Expulsion of Intrauterine Device study. Am. J. Obstet. Gynecol..

[94] Heinemann K., Barnett C., Reed S., Möhner S., Do Minh T. (2017). IUD use among parous women and risk of uterine perforation: A secondary analysis. Contraception.

[95] Fassett M.J., Reed S.D., Rothman K.J., Pisa F.M., Schoendorf J., Wahdan Y., Peipert J.F., Gatz J., Ritchey M.E., Armstrong M.A.M. (2023). Risks of Uterine Perforation and Expulsion Associated With Intrauterine Devices. Obstet. Gynecol..

[96] Armstrong M.A., Raine-Bennett T., Reed S.D., Gatz J., Getahun D., Schoendorf J., Postlethwaite D., Fassett M.J., Peipert J.F., Saltus C.W. (2022). Association of the Timing of Postpartum Intrauterine Device Insertion and Breastfeeding With Risks of Intrauterine Device Expulsion. JAMA Netw. Open.

[97] Getahun D., Fassett M.J., Gatz J., Armstrong M.A., Peipert J.F., Raine-Bennett T., Reed S.D., Zhou X., Schoendorf J., Postlethwaite D. (2022). Association between menorrhagia and risk of intrauterine device-related uterine perforation and device expulsion: Results from the Association of Uterine Perforation and Expulsion of Intrauterine Device study. Am. J. Obstet. Gynecol..

[98] Anthony M.S., Zhou X., Schoendorf J., Reed S.D., Getahun D., Armstrong M.A.M., Gatz J., Peipert J.F., Raine-Bennett T., Fassett M.J. (2022). Demographic, Reproductive, and Medical Risk Factors for Intrauterine Device Expulsion. Obstet. Gynecol..

[99] Siegelmann-Danieli N., Katzir I., Landes J.V., Segal Y., Bachar R., Rabinovich H.R., Bialik M., Azuri J., Porath A., Lomnicky Y. (2018). Does levonorgestrel-releasing intrauterine system increase breast cancer risk in peri-menopausal women? An HMO perspective. Breast Cancer Res. Treat..

[100] Hadizadeh F., Koteci A., Karlsson T., Ek W.E., Johansson Å. (2025). Hormonal Contraceptive Formulations and Breast Cancer Risk in Adolescents and Premenopausal Women. JAMA Oncol..

[101] Bosco-Lévy P., Gouverneur A., Langlade C., Miremont G., Pariente A. (2019). Safety of levonorgestrel 52 mg intrauterine system compared to copper intrauterine device: A population-based cohort study. Contraception.

[102] Roland N., Neumann A., Hoisnard L., Duranteau L., Froelich S., Zureik M., Weill A. (2024). Use of progestogens and the risk of intracranial meningioma: National case-control study. BMJ.

[103] Hopelian N.G., Simmons R.G., Sanders J.N., Ward K., Jenkins S.M., Espey E., Turok D.K. (2021). Comparison of levonorgestrel level and creamatocrit in milk following immediate versus delayed postpartum placement of the levonorgestrel IUD. BMC Womens Health.

[104] Levi E.E., Findley M.K., Avila K., Bryant A.G. (2018). Placement of Levonorgestrel Intrauterine Device at the Time of Cesarean Delivery and the Effect on Breastfeeding Duration. Breastfeed. Med..

[105] Lichtenstein Liljeblad K. (2023). Long-Acting Reversible Contraception (LARC) after Pregnancy and Childbirth. Ph.D. Thesis.

[106] Marangoni M., Laporte M., Surita F., Kraft M.B., Bahamondes L., Juliato C.R.T. (2021). One-year follow up on post-placental IUD insertion: A randomized clinical trial. Acta Obstet. Gynecol. Scand..

[107] Baldwin M.K., Edelman A.B., Lim J.Y., Nichols M.D., Bednarek P.H., Jensen J.T. (2016). Intrauterine device placement at 3 versus 6 weeks postpartum: A randomized trial. Contraception.

[108] Blumenthal P.D., Lerma K., Bhamrah R., Singh S. (2018). Comparative safety and efficacy of a dedicated postpartum IUD inserter versus forceps for immediate postpartum IUD insertion: A randomized trial. Contraception.

[109] Lerma K., Bhamrah R., Singh S., Blumenthal P.D. (2020). Importance of the delivery-to-insertion interval in immediate postpartum intrauterine device insertion: A secondary analysis. Int. J. Gynaecol. Obstet..

[110] Bento Herculano T., Batista A.A.L., Juliato C.R.T., Rehder P.M., Surita F. (2026). Manual versus forceps postplacental IUD insertion after vaginal delivery: A randomized clinical trial. Int. J. Gynaecol. Obstet..

[111] Seleem M., Sedik M.M., Megahed A.M.M., Nabil H. (2023). Conventional manual technique of post placental IUD insertion versus intra-cesarean post placental introducer withdrawal IUD insertion technique: A new standardized technique for IUD insertion during cesarean section: A randomized controlled trial. BMC Pregnancy Childbirth.

[112] Nitesh C., Sharma C., Shekhar S., Singh P., Jhirwal M., Gothwal M., Gupta M.K. (2023). Does change in the shape of intrauterine contraceptive device Cu 375 and Cu T 380A affect the expulsion after post-partum insertion—A prospective randomized control trial. Eur. J. Contracept. Reprod. Health Care.

[113] Kaiser J.E., Turok D.K., Gero A., Gawron L.M., Simmons R.G., Sanders J.N. (2023). One-year pregnancy and continuation rates after placement of levonorgestrel or copper intrauterine devices for emergency contraception: A randomized controlled trial. Am. J. Obstet. Gynecol..

[114] Fay K.E., Clement A.C., Gero A., Kaiser J.E., Sanders J.N., BakenRa A.A., Turok D.K. (2021). Rates of pregnancy among levonorgestrel and copper intrauterine emergency contraception initiators: Implications for backup contraception recommendations. Contraception.

[115] BakenRa A., Gero A., Sanders J., Simmons R., Fay K., Turok D.K. (2021). Pregnancy Risk by Frequency and Timing of Unprotected Intercourse Before Intrauterine Device Placement for Emergency Contraception. Obstet. Gynecol..

[116] Boraas C.M., Sanders J.N., Schwarz E.B., Thompson I., Turok D.K. (2021). Risk of Pregnancy With Levonorgestrel-Releasing Intrauterine System Placement 6-14 Days After Unprotected Sexual Intercourse. Obstet. Gynecol..

[117] Nourse S.E., Woodcock A.L., Brown K.S., Gero A., Gawron L.M., Turok D.K. (2025). Estimating emergency contraception efficacy with levonorgestrel and copper intrauterine devices. Contraception.

[118] Kaplan J., Turok D.K., Gero A., Kaiser J.E., Simmons R.G., Fay K.E. (2023). Switching and discontinuation of participant-masked randomization to a copper or levonorgestrel intrauterine device when presenting for emergency contraception. Contraception.

[119] Chen B.A., Eisenberg D.L., Schreiber C.A., Turok D.K., Olariu A.I., Creinin M.D. (2020). Bleeding changes after levonorgestrel 52-mg intrauterine system insertion for contraception in women with self-reported heavy menstrual bleeding. Am. J. Obstet. Gynecol..

[120] Kiseli M., Kayikcioglu F., Evliyaoglu O., Haberal A. (2016). Comparison of Therapeutic Efficacies of Norethisterone, Tranexamic Acid and Levonorgestrel-Releasing Intrauterine System for the Treatment of Heavy Menstrual Bleeding: A Randomized Controlled Study. Gynecol. Obstet. Invest..

[121] Ilyin A.B., Khasanov A.A., Suturina L.V., Borisova N.I., Reshetov Z.S., Foidart J.-M., Déri J.A., Tóth V. (2021). Comparison of two levonorgestrel-releasing intrauterine systems for the treatment of heavy menstrual bleeding: A randomised, controlled, phase 3 trial. Eur. J. Contracept. Reprod. Health Care.

[122] Kai J., Middleton L., Daniels J., Pattison H., Tryposkiadis K., Gupta J. (2016). Usual medical treatments or levonorgestrel-IUS for women with heavy menstrual bleeding: Long-term randomised pragmatic trial in primary care. Br. J. Gen. Pract..

[123] Kai J., Dutton B., Vinogradova Y., Hilken N., Gupta J., Daniels J. (2023). Rates of medical or surgical treatment for women with heavy menstrual bleeding: The ECLIPSE trial 10-year observational follow-up study. Health Technol. Assess..

[124] Matteson K.A., Valcin J., Raker C.A., Clark M.A. (2023). A randomized trial comparing the 52-mg levonorgestrel system with combination oral contraceptives for treatment of heavy menstrual bleeding. Am. J. Obstet. Gynecol..

[125] Huijs D.P.C., Derickx A.J.M., Beelen P., Leemans J.C., van Kuijk S.M., Bongers M.Y., Geomini P.M. (2024). A 52-mg levonorgestrel-releasing intrauterine system vs bipolar radiofrequency nonresectoscopic endometrial ablation in women with heavy menstrual bleeding: Long-term follow-up of a multicenter randomized controlled trial. Am. J. Obstet. Gynecol..

[126] Beelen P., van der Velde M.G.A.M., Herman M.C., Geomini P.M., Brink M.J.v.D., Duijnhoven R.G., Bongers M.Y. (2021). Treatment of women with heavy menstrual bleeding: Results of a prospective cohort study alongside a randomised controlled trial. Eur. J. Obstet. Gynecol. Reprod. Biol..

[127] Ørbo A., Arnes M., Vereide A.B., Straume B. (2016). Relapse risk of endometrial hyperplasia after treatment with the levonorgestrel-impregnated intrauterine system or oral progestogens. BJOG.

[128] Goh C.S.Y., Loh M.J.M., Lim W.W., Ang J.X., Nadarajah R., Yong T.T., Tong P., Yeo Y.C., Phoon J.W.L. (2024). A multi-centre randomised controlled trial comparing megestrol acetate to levonorgestrel-intrauterine system in fertility sparing treatment of atypical endometrial hyperplasia. J. Assist Reprod. Genet..

[129] Baxter E., Robledo K.P., Janda M., Obermair A. (2026). Long-term oncological and reproductive outcomes following treatment with the levonorgestrel intra-uterine device for endometrial hyperplasia and adenocarcinoma: Results from the feMMe phase 2 randomized clinical trial. Int. J. Gynecol. Cancer.

[130] Xu Z., Yang B., Guan J., Shan W., Liao J., Shao W., Chen X. (2023). Comparison of the effect of oral megestrol acetate with or without levonorgestrel-intrauterine system on fertility-preserving treatment in patients with early-stage endometrial cancer: A prospective, open-label, randomized controlled phase II trial (ClinicalTrials.gov NCT03241914). J. Gynecol. Oncol..

[131] Zhang J., Zhu C., Yan L., Wang Y., Zhu Q., He C., He X., Ji S., Tian Y., Xie L. (2023). Comparing levonorgestrel intrauterine system with hysteroscopic niche resection in women with postmenstrual spotting related to a niche in the uterine cesarean scar: A randomized, open-label, controlled trial. Am. J. Obstet. Gynecol..

[132] Yan L., Zhu C., Xia W., Wang Y., He C., Zhu Q., Huirne J.A., Mol B.W., Zhang J. (2026). Levonorgestrel intrauterine system vs hysteroscopic niche resection for postmenstrual spotting: A 3-year randomized trial follow-up. Am. J. Obstet. Gynecol..

[133] Fels L.M., Costescu D., Vieira C.S., Peipert J.F., Lukkari-Lax E., Hofmann B.M., Reinecke I., Klein S., Wiesinger K., Lindenthal B. (2023). The effect of a combined indomethacin and levonorgestrel-releasing intrauterine system on short-term postplacement bleeding profile: A randomized proof-of-concept trial. Am. J. Obstet. Gynecol..

[134] Hofmann B.M., Ahola M., Fels L.M., Klein S., Lindenthal B., Pihlaja J. (2023). Development of an Intrauterine Device Releasing Both Indomethacin and Levonorgestrel During the First Months of Use: Pharmacokinetic Characterization in Healthy Women. Clin. Pharmacokinet..

[135] World Health Organization (2025). Medical Eligibility Criteria for Contraceptive Use.

[136] Mørch L.S., Skovlund C.W., Hannaford P.C., Iversen L., Fielding S., Lidegaard Ø. (2017). Contemporary hormonal contraception and the risk of breast cancer. N. Engl. J. Med..

[137] Mørch L.S., Meaidi A., Corn G., Hargreave M., Wessel Skovlund C. (2024). Breast cancer in users of levonorgestrel-releasing intrauterine systems. JAMA.

[138] Skovlund C.W., Mørch L.S., Kessing L.V., Lange T., Lidegaard Ø. (2018). Association of hormonal contraception with suicide attempts and suicides. Am. J. Psychiatry.

[139] Salcedo J., Cleland K., Bartz D., Thompson I. (2023). Society of Family Planning Clinical Recommendation: Emergency contraception. Contraception.

[140] National Institute for Health and Care Excellence (2018). Heavy menstrual bleeding: Assessment and management. NICE Guideline NG88.

[141] Peters M.D.J., Godfrey C., McInerney P., Munn Z., Tricco A.C., Khalil H., Aromataris E., Munn Z. (2020). Chapter 11: Scoping reviews. JBI Manual for Evidence Synthesis.

[142] Tufanaru C., Munn Z., Aromataris E., Campbell J., Hopp L., Aromataris E., Munn Z. (2020). Chapter 3: Systematic reviews of effectiveness. JBI Manual for Evidence Synthesis.

[143] Moola S., Munn Z., Tufanaru C., Aromataris E., Sears K., Sfetcu R., Currie M., Lisy K., Qureshi R., Mattis P., Aromataris E., Munn Z. (2020). Chapter 7: Systematic reviews of etiology and risk. JBI Manual for Evidence Synthesis.

[144] Munn Z., Barker T.H., Moola S., Tufanaru C., Stern C., McArthur A., Stephenson M., Aromataris E. (2020). Methodological quality of case series studies: An introduction to the JBI critical appraisal tool. JBI Evid. Synth..

