# Supplementary material for: Advances in Intrauterine Contraception and Therapeutic Intrauterine Devices, 2016–2026: A Targeted Scoping Review
Source: J Clin Med. 2026 Oct 26;15(21):8338. doi: 10.3390/jcm15218338 (PMC13763720; doi:10.3390/jcm15218338)
Supplement: Supplementary file 1 [file jcm-15-08338-s001.zip › jcm-4595375-supplementary_revised_proofread.pdf]

Supplementary Materials

Advances in Intrauterine Contraception and Therapeutic Intrauterine Devices, 2016–2026: A Targeted Scoping Review

Supplementary Table S1. Design-to-Tool Mapping and JBI Critical Appraisal of Included Studies

Table S1 (overview) maps each included report to the design actually appraised, the JBI checklist applied and the reason; the tool used in the original submission is shown for transparency (see also Supplementary Table S7). Reference numbers refer to the reference list of the revised manuscript. For six trials (IDs 1, 19, 21, 38, 77 and 83), the cited reference is a later report, review or thesis covering the same trial; the design, tool and appraisal shown refer to the trial itself. Y = yes; N = no; U = unclear; NA = not applicable; – = item-level appraisal pending. Ten added reports whose full text could not be obtained were appraised from the abstract (and parent-trial report where applicable); items not reported there were coded U; these rows are marked † (see Table S1g).

Table S1 (overview). Design-to-tool mapping of the 114 included reports

ID [ref.]	Study	Design as reported	Tool in original submission	Tool applied in revision	Rationale
1 [76]	WHO 7-yr LNG-IUD vs TCu380A trial, reported in Ti 2020	Open-label 7-year randomized controlled comparative trial	RCT checklist	RCT checklist	Randomized comparison with concurrent control
2 [45]	Liletta LNG release-rate study, 2016	Device-content / release-rate study nested in phase 3 program	Quasi-experimental checklist	None (mechanistic; described)	Laboratory measurement of removed devices; no clinical outcome
3 [13]	IUB SCu380A pilot study, 2016	Prospective pilot study	Quasi-experimental checklist	Case-series checklist	Single-arm prospective pilot; no comparator
4 [18]	EURAS-IUD 5-yr extension, 2017	Prospective non-interventional cohort; 5-year extension	Cohort checklist	Cohort checklist	Comparative cohort of LNG-IUD and copper-IUD users
5 [20]	Immediate postpartum LNG-IUD RCT, 2017	Noninferiority randomized controlled trial	RCT checklist	RCT checklist	Randomized comparison of insertion timing
6 [77]	Mirena extended-use chart review (Brazil), 2018	Retrospective chart review	Quasi-experimental checklist	Case-series checklist	Single group of long-term continuers; no comparator
7 [11]	Gyn-CS vs TCu380A intracesarean RCT, 2018	Randomized controlled trial	RCT checklist	RCT checklist	Randomized comparison with TCu380A
8 [86]	Gyn-CS clinical experience (single-arm), 2018	Prospective nonrandomized clinical study	Quasi-experimental checklist	Case-series checklist	Single-arm prospective series; no comparator
9 [79]	Liletta 5-yr efficacy (ACCESS IUS), 2019	Phase 3 multicenter clinical trial	Quasi-experimental checklist	Case-series checklist	Single-arm phase 3 trial; no comparator
10 [83]	IUB Ballerine MIDI (Switzerland), 2019	Retrospective observational study	Quasi-experimental checklist	Case-series checklist	Single-group retrospective series; no comparator
11 [80]	Liletta 6-yr efficacy (ACCESS IUS), 2020	Ongoing phase 3 multicenter follow-up	Quasi-experimental checklist	Case-series checklist	Single-arm phase 3 follow-up; no comparator
12 [84]	IUB Ballerine MIDI (Israel), 2020	Retrospective observational study	Quasi-experimental checklist	Case-series checklist	Single-group retrospective series; no comparator
13 [21]	Postplacental TCu380A vs LNG-IUD RCT, 2020	Randomized controlled trial	RCT checklist	RCT checklist	Randomized comparison of two devices
14 [119]	Liletta HMB subgroup analysis, 2020	Prospective cohort/subgroup analysis	Quasi-experimental checklist	Case-series checklist	Subgroup of a single-arm trial; no comparator
15 [24]	LNG vs copper IUD for EC (NEJM), 2021	Randomized noninferiority trial	RCT checklist	RCT checklist	Randomized noninferiority comparison
16 [32]	Cu-UPA proof-of-concept RCT, 2021	Single-blind randomized proof-of-concept study	RCT checklist	RCT checklist	Randomized dose comparison
17 [26]	LNG-IUD vs ablation, MIRA trial, 2021	Multicenter randomized controlled trial	RCT checklist	RCT checklist	Randomized comparison with ablation
18 [10]	NTCu380-Mini vs TCu380A RCT, 2022	Participant-blinded randomized multicenter trial	RCT checklist	RCT checklist	Randomized comparison with TCu380A
19 [19]	APEX-IUD perforation cohort, reported in Anthony 2021	Large multisite retrospective cohort	Cohort checklist	Cohort checklist	Comparative cohort with exposure groups

ID [ref.]	Study	Design as reported	Tool in original submission	Tool applied in revision	Rationale
20 [93]	APEX-IUD perforation/expulsion (AJOG), 2022	Retrospective cohort	Cohort checklist	Cohort checklist	Comparative cohort by device type
21 [8]	Liletta 8-yr efficacy (ACCESS IUS), reported in Creinin 2023	Phase 3 multicenter extended follow-up	Quasi-experimental checklist	Case-series checklist	Single-arm phase 3 extension; no comparator
22 [9]	Mirena Extension Trial, 8 yr, 2022	Multicenter single-arm extension study	Quasi-experimental checklist	Case-series checklist	Single-arm extension study; no comparator
23 [52]	Kyleena Satisfaction study, 2023	Prospective multinational observational study	Quasi-experimental checklist	Case-series checklist	Single-group observational study; no comparator
24 [60]	LNG 13.5 mg vs Nova T Cu380 RCT, 2023	Randomized phase 4 clinical trial	RCT checklist	RCT checklist	Randomized comparison with copper IUD
25 [46]	Population PK modeling, 3 LNG-IUD doses, 2023	Population pharmacokinetic modeling using pooled clinical-trial data	Quasi-experimental checklist	None (mechanistic; described)	Population pharmacokinetic modelling; no clinical outcome
26 [81]	Liletta beyond 8-yr research letter, 2023	Extended phase 3 follow-up / research letter	Quasi-experimental checklist	Case-series checklist	Single-arm phase 3 extension (research letter)
27 [113]	LNG vs copper EC, 1-yr follow-up, 2023	Randomized-trial follow-up	RCT checklist	RCT checklist	Follow-up of randomized cohort
28 [25]	LNG-IUD for HMB, treatment trial, 2023	Prospective multicenter treatment trial	Quasi-experimental checklist	Case-series checklist	Single-arm treatment trial; no comparator
29 [124]	LNG-IUD vs COC, pragmatic RCT, 2023	Pragmatic randomized trial	RCT checklist	RCT checklist	Randomized comparison with combined oral contraceptives
30 [12]	EURAS-LCS12 device-shape analysis, 2024	Secondary analysis of prospective non-interventional cohort	Cohort checklist	Cohort checklist	Comparative cohort by device shape
31 [125]	LNG-IUD vs ablation, long-term (MIRA), 2024	Long-term follow-up of multicenter RCT	RCT checklist	RCT checklist	Long-term follow-up of randomized cohort
32 [27]	LNG-IUD vs megestrol acetate, AEH RCT, 2024	Single-center open-label randomized controlled trial	RCT checklist	RCT checklist	Randomized comparison with megestrol acetate
33 [15]	Low-dose Cu IUD, phase 3 (3-yr), 2025	Phase 3 multicenter single-arm trial	Quasi-experimental checklist	Case-series checklist	Single-arm phase 3 trial; no comparator
34 [16]	Low-dose Cu IUD, phase 3 (5-yr), 2026	Phase 3 multicenter extended follow-up	Quasi-experimental checklist	Case-series checklist	Single-arm phase 3 extension; no comparator
35 [89]	NTCu380-Mini vs TCu380A, secondary analysis, 2026	Secondary analysis of randomized multicenter trial	RCT checklist	RCT checklist	Secondary analysis of randomized trial
36 [17]	EURAS-LCS12 alloy (Ag/Au) analysis, 2026	Exploratory cohort analysis	Cohort checklist	Cohort checklist	Comparative cohort by device alloy
37 [82]	Mirena residual-LNG device study, 2026	Prospective device-content study	Quasi-experimental checklist	None (mechanistic; described)	Laboratory measurement of removed devices; no clinical outcome
38 [48]	Kyleena precursor, 5-yr phase III, reported in Nelson 2017	Phase III randomized clinical trial with 5-year extension of the 19.5-mg system	RCT checklist	RCT checklist	Randomized comparison of two LNG doses
39 [23]	3 IUDs in adolescents, RCT, 2024	Randomized clinical trial	RCT checklist	RCT checklist	Randomized three-arm comparison
40 [22]	Early vs interval postpartum IUD RCT, 2023	Randomized noninferiority clinical trial	RCT checklist	RCT checklist	Randomized comparison of placement timing
41 [64]	Danish ectopic-pregnancy cohort by LNG dose, 2023	Nationwide register-based cohort study / research letter	Cohort checklist	Cohort checklist	Register-based cohort comparing LNG doses
42 [28]	LNG-IUD vs dienogest, adenomyosis RCT, 2023	Comparative interventional study described as randomized; assignment by odd/even first-visit date	RCT checklist	Quasi-experimental checklist	Alternating (odd/even date) allocation with concurrent control; not truly randomized
43 [127]	Ørbo, BJOG 2016	Multicentre RCT; 24-month post-treatment follow-up	— (added in revision)	RCT checklist	Follow-up or secondary analysis by randomized arm
44 [107]	Baldwin, Contraception 2016	Randomized controlled trial	— (added in revision)	RCT checklist	Comparison between randomized groups
45 [55]	Gemzell-Danielsson, Contraception 2016	Phase III single-arm trial	— (added in revision)	Case-series checklist	Single-arm trial or single-group series; no comparator

ID [ref.]	Study	Design as reported	Tool in original submission	Tool applied in revision	Rationale
46 [120]	Kiseli, Gynecol Obstet Invest 2016	Randomized controlled trial	— (added in revision)	RCT checklist	Comparison between randomized groups
47 [58]	Apter, Fertil Steril 2016	Randomized open-label phase III trial	— (added in revision)	RCT checklist	Comparison between randomized groups
48 [73]	Turok, Am J Obstet Gynecol 2016	Prospective analysis within single-arm phase 3 trial	— (added in revision)	Case-series checklist	Single-arm trial or single-group series; no comparator
49 [59]	Borgatta, Eur J Contracept Reprod Health Care 2016	Randomized open-label phase III trial	— (added in revision)	RCT checklist	Comparison between randomized groups
50 [122]	Kai, Br J Gen Pract 2016	Pragmatic multicentre RCT; 5-year follow-up	— (added in revision)	RCT checklist	Follow-up or secondary analysis by randomized arm
51 [56]	Fan, Contraception 2017	Phase III single-arm trial (3 years)	— (added in revision)	Case-series checklist	Single-arm trial or single-group series; no comparator
52 [78]	McNicholas, Am J Obstet Gynecol 2017	Prospective cohort of extended use	— (added in revision)	Case-series checklist	Single-arm trial or single-group series; no comparator
53 [94]	Heinemann, Contraception 2017	Secondary analysis of prospective cohort	— (added in revision)	Cohort checklist	Comparison of nonrandomized exposure groups
54 [14]	Reeves, Contraception 2017	Participant-blinded RCT (2:1)	— (added in revision)	RCT checklist	Comparison between randomized groups
55 [57]	Gemzell-Danielsson, Contraception 2017	Pooled analysis of three phase III trials	— (added in revision)	Case-series checklist	Single-arm trial or single-group series; no comparator
56 [99]	Siegelmann-Danieli, Breast Cancer Res Treat 2018	Retrospective matched cohort (HMO)	— (added in revision)	Cohort checklist	Comparison of nonrandomized exposure groups
57 [70]	Darney, Contraception 2018	Analysis within single-arm phase 3 trial	— (added in revision)	Case-series checklist	Single-arm trial or single-group series; no comparator
58 [71]	Schreiber, Eur J Contracept Reprod Health Care 2018	Analysis within single-arm phase 3 trial	— (added in revision)	Case-series checklist	Single-arm trial or single-group series; no comparator
59 [62]	Law, Contraception 2018	Retrospective claims cohort	— (added in revision)	Cohort checklist	Comparison of nonrandomized exposure groups
60 [108]	Blumenthal, Contraception 2018	Randomized controlled trial	— (added in revision)	RCT checklist	Comparison between randomized groups
61 [104]	Levi, Breastfeed Med 2018	Secondary analysis of RCT	— (added in revision)	RCT checklist	Follow-up or secondary analysis by randomized arm
62 [66]	Graner, Acta Obstet Gynecol Scand 2019	Hospital-based historical cohort	— (added in revision)	Cohort checklist	Comparison of nonrandomized exposure groups
63 [101]	Bosco-Lévy, Contraception 2019	Propensity-matched claims cohort	— (added in revision)	Cohort checklist	Comparison of nonrandomized exposure groups
64 [49]	Beckert, Eur J Contracept Reprod Health Care 2019	Pooled analysis of phase II and III trials	— (added in revision)	Case-series checklist	Single-arm trial or single-group series; no comparator
65 [109]	Lerma, Int J Gynaecol Obstet 2020	Secondary analysis of two related studies	— (added in revision)	Cohort checklist	Comparison of nonrandomized exposure groups
66 [92]	Turok, Obstet Gynecol 2020	Phase 2 single-arm trial with 2-year extension	— (added in revision)	Case-series checklist	Single-arm trial or single-group series; no comparator
67 [51]	Beckert, Eur J Contracept Reprod Health Care 2020	Prospective observational study (baseline analysis)	— (added in revision)	Case-series checklist	Single-arm trial or single-group series; no comparator
68 [47]	Hofmann, Eur J Contracept Reprod Health Care 2020	Integrated population pharmacokinetic analysis	— (added in revision)	None (mechanistic; described)	Pharmacokinetic, drug-level or modelling study; no clinical comparison
69 [74]	Carr, Contraception 2021	Analysis within single-arm phase 3 trial	— (added in revision)	Case-series checklist	Single-arm trial or single-group series; no comparator
70 [50]	Frenz, Eur J Contracept Reprod Health Care 2021	Bleeding-prediction modelling study	— (added in revision)	None (mechanistic; described)	Pharmacokinetic, drug-level or modelling study; no clinical comparison
71 [126]	Beelen, Eur J Obstet Gynecol Reprod Biol 2021	Prospective preference cohort alongside RCT	— (added in revision)	Cohort checklist	Comparison of nonrandomized exposure groups
72 [106]	Marangoni, Acta Obstet Gynecol Scand 2021	Randomized controlled trial; 1-year follow-up	— (added in revision)	RCT checklist	Follow-up or secondary analysis by randomized arm
73 [103]	Hopelian, BMC Womens Health 2021	Breast-milk substudy of RCT	— (added in revision)	None (mechanistic; described)	Pharmacokinetic, drug-level or modelling study; no clinical comparison
74 [72]	Gilliam, Contraception 2021	Analysis within single-arm phase 3 trial	— (added in revision)	Cohort checklist	Trial population analysed by nonrandomized exposure
75 [116]	Boraas, Obstet Gynecol 2021	Prospective observational cohort (research letter)	— (added in revision)	Case-series checklist	Single-arm trial or single-group series; no comparator

ID [ref.]	Study	Design as reported	Tool in original submission	Tool applied in revision	Rationale
76 [85]	Akintomide, Eur J Contracept Reprod Health Care 2021	Secondary analysis of prospective cohort	— (added in revision)	Cohort checklist	Comparison of nonrandomized exposure groups
77 [129]	Baxter, Int J Gynecol Cancer 2026 (feMMe trial)	Phase II RCT	— (added in revision)	RCT checklist	Comparison between randomized groups
78 [114]	Fay, Contraception 2021	Secondary analysis of RCT	— (added in revision)	Cohort checklist	Trial population analysed by nonrandomized exposure
79 [121]	Ilyin, Eur J Contracept Reprod Health Care 2021	Randomized non-inferiority phase 3 trial	— (added in revision)	RCT checklist	Comparison between randomized groups
80 [115]	BakenRa, Obstet Gynecol 2021	Secondary analysis of RCT	— (added in revision)	Cohort checklist	Trial population analysed by nonrandomized exposure
81 [53]	Stovall, Eur J Contracept Reprod Health Care 2021	Prospective observational study	— (added in revision)	Case-series checklist	Single-arm trial or single-group series; no comparator
82 [87]	Kutlucan, Life (Basel) 2022	Prospective observational case series	— (added in revision)	Case-series checklist	Single-arm trial or single-group series; no comparator
83 [105]	Lichtenstein Liljeblad, PhD thesis, Karolinska Institutet 2023	Randomized non-inferiority trial (stopped early)	— (added in revision)	RCT checklist	Comparison between randomized groups
84 [96]	Armstrong, JAMA Netw Open 2022	Retrospective cohort	— (added in revision)	Cohort checklist	Comparison of nonrandomized exposure groups
85 [97]	Getahun, Am J Obstet Gynecol 2022	Retrospective cohort	— (added in revision)	Cohort checklist	Comparison of nonrandomized exposure groups
86 [65]	Kopp-Kallner, Obstet Gynecol 2022	Nationwide register cohort	— (added in revision)	Cohort checklist	Comparison of nonrandomized exposure groups
87 [67]	Elgemark, Obstet Gynecol 2022	Retrospective cohort	— (added in revision)	Cohort checklist	Comparison of nonrandomized exposure groups
88 [118]	Kaplan, Contraception 2023	Secondary analysis of RCT	— (added in revision)	RCT checklist	Follow-up or secondary analysis by randomized arm
89 [98]	Anthony, Obstet Gynecol 2022	Retrospective cohort	— (added in revision)	Cohort checklist	Comparison of nonrandomized exposure groups
90 [133]	Fels, Am J Obstet Gynecol 2023	Phase II dose-finding RCT	— (added in revision)	RCT checklist	Comparison between randomized groups
91 [30]	Gezer, Int J Gynaecol Obstet 2023	Randomized controlled trial	— (added in revision)	RCT checklist	Comparison between randomized groups
92 [69]	Roland, JAMA 2023	Nationwide claims cohort (research letter)	— (added in revision)	Cohort checklist	Comparison of nonrandomized exposure groups
93 [130]	Xu, J Gynecol Oncol 2023	Phase II RCT	— (added in revision)	RCT checklist	Comparison between randomized groups
94 [134]	Hofmann, Clin Pharmacokinet 2023	Pharmacokinetic substudy of phase II RCT	— (added in revision)	None (mechanistic; described)	Pharmacokinetic, drug-level or modelling study; no clinical comparison
95 [91]	Bhatla, Int J Gynaecol Obstet 2023	Randomized controlled trial; 5-year follow-up	— (added in revision)	RCT checklist	Follow-up or secondary analysis by randomized arm
96 [63]	Anjos, Contraception 2023	Participant-blinded RCT	— (added in revision)	RCT checklist	Comparison between randomized groups
97 [88]	Che, Contraception 2023	Prospective multicentre cohort	— (added in revision)	Case-series checklist	Single-arm trial or single-group series; no comparator
98 [131]	Zhang, Am J Obstet Gynecol 2023	Randomized open-label trial	— (added in revision)	RCT checklist	Comparison between randomized groups
99 [112]	Nitesh, Eur J Contracept Reprod Health Care 2023	Randomized controlled trial	— (added in revision)	RCT checklist	Comparison between randomized groups
100 [31]	Xu, Gynecol Oncol 2023	Phase II RCT (three arms)	— (added in revision)	RCT checklist	Comparison between randomized groups
101 [111]	Seleem, BMC Pregnancy Childbirth 2023	Randomized controlled trial	— (added in revision)	RCT checklist	Comparison between randomized groups
102 [95]	Fassett, Obstet Gynecol 2023	Summary of retrospective cohort	— (added in revision)	Cohort checklist	Comparison of nonrandomized exposure groups
103 [123]	Kai, Health Technol Assess 2023	10-year observational follow-up of RCT	— (added in revision)	Cohort checklist	Comparison of nonrandomized exposure groups
104 [61]	Perelló-Capó, Contraception 2024	Secondary outcomes of phase 4 RCT	— (added in revision)	RCT checklist	Follow-up or secondary analysis by randomized arm
105 [54]	Römer, Arch Gynecol Obstet 2024	Prospective observational study (national subset)	— (added in revision)	Case-series checklist	Single-arm trial or single-group series; no comparator
106 [90]	Brown, Contraception 2024	Secondary analysis of RCT	— (added in revision)	Cohort checklist	Trial population analysed by nonrandomized exposure
107 [68]	Skovlund, Lancet Reg Health Eur 2024	Nationwide prospective cohort	— (added in revision)	Cohort checklist	Comparison of nonrandomized exposure groups

ID [ref.]	Study	Design as reported	Tool in original submission	Tool applied in revision	Rationale
108 [102]	Roland, BMJ 2024	National case–control study	— (added in revision)	Case–control checklist	Cases and matched controls
109 [75]	Kerns, Am J Obstet Gynecol 2024	Analysis within single-arm phase 3 trial	— (added in revision)	Cohort checklist	Trial population analysed by nonrandomized exposure
110 [128]	Goh, J Assist Reprod Genet 2024	Multicentre phase II RCT	— (added in revision)	RCT checklist	Comparison between randomized groups
111 [117]	Nourse, Contraception 2025	Secondary analysis of RCT	— (added in revision)	Case-series checklist	Single-arm trial or single-group series; no comparator
112 [110]	Bento Herculano, Int J Gynaecol Obstet 2026	Randomized controlled trial	— (added in revision)	RCT checklist	Comparison between randomized groups
113 [132]	Yan, Am J Obstet Gynecol 2026	3-year follow-up of RCT	— (added in revision)	RCT checklist	Follow-up or secondary analysis by randomized arm
114 [100]	Hadizadeh, JAMA Oncol 2025	Nationwide register cohort	— (added in revision)	Cohort checklist	Comparison of nonrandomized exposure groups

1. JBI Critical Appraisal Checklist for Randomized Controlled Trials (13 items)

Item	Criterion
1	Was true randomization used for assignment of participants to treatment groups?
2	Was allocation to treatment groups concealed?
3	Were treatment groups similar at baseline?
4	Were participants blind to treatment assignment?
5	Were those delivering treatment blind to treatment assignment?
6	Were outcome assessors blind to treatment assignment?
7	Were treatment groups treated identically other than the intervention of interest?
8	Was follow-up complete, and if not, were differences between groups in follow-up adequately described and analyzed?
9	Were participants analyzed in the groups to which they were randomized (intention-to-treat)?
10	Were outcomes measured in the same way for all treatment groups?
11	Were outcomes measured in a reliable way?
12	Was appropriate statistical analysis used?
13	Was the trial design appropriate, with any deviation from a standard parallel-group design accounted for in conduct and analysis?

Table S1a. Appraisal results, randomized controlled trials (n = 44; item-level codes for all 44)

ID	Study (first author/trial, journal, year)	Q1	Q2	Q3	Q4	Q5	Q6	Q7	Q8	Q9	Q10	Q11	Q12	Q13	Overall
1 [76]	WHO 7-yr LNG-IUD vs TCu380A trial, reported in Ti 2020	Y	Y	Y	N	N	U	Y	Y	N	Y	Y	Y	Y	Incl.
5 [20]	Immediate postpartum LNG-IUD RCT, 2017	Y	Y	Y	N	N	N	Y	Y	N	Y	Y	Y	Y	Incl.
7 [11]	Gyn-CS vs TCu380A intracesarean RCT, 2018	Y	Y	Y	Y	N	N	Y	Y	N	Y	Y	Y	Y	Incl.

ID	Study (first author/trial, journal, year)	Q1	Q2	Q3	Q4	Q5	Q6	Q7	Q8	Q9	Q10	Q11	Q12	Q13	Overall
13 [21]	Postplacental TCu380A vs LNG-IUD RCT, 2020	Y	Y	N	N	N	N	N	Y	N	Y	Y	Y	Y	Incl.
15 [24]	LNG vs copper IUD for EC (NEJM), 2021	Y	Y	N	Y	N	U	Y	Y	N	Y	Y	Y	Y	Incl.
16 [32]	Cu-UPA proof-of-concept RCT, 2021	Y	U	Y	Y	N	U	Y	Y	N	Y	Y	Y	Y	Incl.
17 [26]	LNG-IUD vs ablation, MIRA trial, 2021	Y	Y	Y	N	N	N	Y	Y	Y	Y	Y	Y	Y	Incl.
18 [10]	NTCu380-Mini vs TCu380A RCT, 2022	Y	Y	Y	Y	N	U	Y	Y	N	Y	Y	Y	Y	Incl.
24 [60]	LNG 13.5 mg vs Nova T Cu380 RCT, 2023	Y	U	Y	Y	N	Y	Y	Y	U	Y	Y	Y	Y	Incl.
27 [113]	LNG vs copper EC, 1-yr follow-up, 2023	Y	Y	N	N	N	U	Y	Y	Y	Y	Y	Y	Y	Incl.
29 [124]	LNG-IUD vs COC, pragmatic RCT, 2023	Y	U	Y	N	N	N	Y	Y	Y	Y	Y	Y	Y	Incl.
31 [125]	LNG-IUD vs ablation, long-term (MIRA), 2024	Y	Y	Y	N	N	N	Y	N	N	Y	Y	Y	Y	Incl.
32 [27]	LNG-IUD vs megestrol acetate, AEH RCT, 2024	Y	Y	Y	N	N	U	Y	Y	Y	Y	Y	Y	Y	Incl.
35 [89]	NTCu380-Mini vs TCu380A, secondary analysis, 2026	Y	Y	Y	Y	N	U	Y	Y	N	Y	Y	Y	Y	Incl.
38 [48]	Kyleena precursor, 5-yr phase III, reported in Nelson 2017	Y	U	U	U	U	U	Y	Y	U	Y	Y	Y	Y	Incl.
39 [23]	3 IUDs in adolescents, RCT, 2024	Y	U	Y	N	N	N	Y	Y	Y	Y	Y	Y	Y	Incl.
40 [22]	Early vs interval postpartum IUD RCT, 2023	Y	Y	Y	N	N	Y	Y	N	N	Y	Y	Y	Y	Incl.
43 [127]	Ørbo, BJOG 2016	U	U	U	N	N	Y	Y	Y	N	Y	Y	Y	Y	Incl.
44 [107]	Baldwin, Contraception 2016	Y	Y	Y	N	N	N	Y	U	Y	Y	Y	Y	U	Incl.
46 [120]	Kiseli, Gynecol Obstet Invest 2016 †	U	U	U	N	N	U	U	N	N	Y	Y	U	U	Incl.
47 [58]	Apter, Fertil Steril 2016	Y	U	Y	N	N	N	Y	Y	Y	Y	Y	Y	Y	Incl.
49 [59]	Borgatta, Eur J Contracept Reprod Health Care 2016 †	U	U	Y	N	N	N	U	U	Y	Y	Y	U	Y	Incl.
50 [122]	Kai, Br J Gen Pract 2016	Y	Y	Y	N	N	N	Y	N	Y	Y	Y	Y	Y	Incl.
54 [14]	Reeves, Contraception 2017	U	Y	Y	Y	N	U	Y	Y	Y	Y	Y	Y	Y	Incl.
60 [108]	Blumenthal, Contraception 2018	Y	Y	Y	Y	N	U	Y	Y	N	Y	Y	Y	Y	Incl.
61 [104]	Levi, Breastfeed Med 2018 †	U	U	U	N	N	U	U	U	U	Y	U	Y	U	Incl.

ID	Study (first author/trial, journal, year)	Q1	Q2	Q3	Q4	Q5	Q6	Q7	Q8	Q9	Q10	Q11	Q12	Q13	Overall
72 [106]	Marangoni, Acta Obstet Gynecol Scand 2021	Y	Y	U	N	N	U	Y	Y	U	Y	Y	Y	Y	Incl.
77 [129]	Baxter, Int J Gynecol Cancer 2026 (feMMe trial)	Y	Y	U	N	N	Y	Y	U	Y	Y	Y	Y	U	Incl.
79 [121]	Ilyin, Eur J Contracept Reprod Health Care 2021 †	U	U	U	U	N	U	Y	Y	N	Y	Y	Y	Y	Incl.
83 [105]	Lichtenstein Liljeblad, PhD thesis, Karolinska Institutet 2023	U	U	Y	N	N	N	Y	Y	Y	Y	U	Y	Y	Incl.
88 [118]	Kaplan, Contraception 2023	Y	Y	N	N	N	N	Y	Y	U	Y	U	Y	Y	Incl.
90 [133]	Fels, Am J Obstet Gynecol 2023	U	U	Y	Y	N	Y	Y	Y	N	Y	Y	Y	Y	Incl.
91 [30]	Gezer, Int J Gynaecol Obstet 2023 †	U	U	U	N	N	U	U	Y	U	Y	Y	U	Y	Incl.
93 [130]	Xu, J Gynecol Oncol 2023	Y	Y	Y	N	N	Y	Y	Y	N	Y	Y	Y	N	Incl.
95 [91]	Bhatla, Int J Gynaecol Obstet 2023 †	U	U	U	U	N	U	Y	U	U	Y	Y	Y	Y	Incl.
96 [63]	Anjos, Contraception 2023	Y	Y	Y	Y	N	Y	Y	Y	Y	Y	Y	Y	Y	Incl.
98 [131]	Zhang, Am J Obstet Gynecol 2023	Y	Y	Y	N	N	U	U	Y	Y	Y	Y	Y	Y	Incl.
99 [112]	Nitesh, Eur J Contracept Reprod Health Care 2023 †	U	U	U	U	N	U	Y	U	N	Y	Y	Y	Y	Incl.
100 [31]	Xu, Gynecol Oncol 2023	Y	Y	U	N	N	Y	Y	Y	N	Y	Y	Y	N	Incl.
101 [111]	Seleem, BMC Pregnancy Childbirth 2023	U	U	Y	Y	N	U	Y	Y	N	Y	Y	Y	U	Incl.
104 [61]	Perelló-Capó, Contraception 2024	U	U	Y	N	N	Y	Y	N	N	Y	Y	Y	Y	Incl.
110 [128]	Goh, J Assist Reprod Genet 2024	U	Y	U	N	N	U	Y	Y	N	Y	Y	N	U	Incl.
112 [110]	Bento Herculano, Int J Gynaecol Obstet 2026	Y	Y	Y	Y	N	U	Y	N	N	Y	U	Y	Y	Incl.
113 [132]	Yan, Am J Obstet Gynecol 2026	Y	Y	Y	N	N	U	U	Y	Y	Y	U	Y	Y	Incl.

2. JBI Critical Appraisal Checklist for Cohort Studies (11 items)

Item	Criterion
1	Were the two groups similar and recruited from the same population?
2	Were the exposures measured similarly to assign people to exposed and unexposed groups?
3	Was the exposure measured in a valid and reliable way?
4	Were confounding factors identified?
5	Were strategies to deal with confounding factors stated?
6	Were the groups/participants free of the outcome at the start of the study (or at the moment of exposure)?
7	Were the outcomes measured in a valid and reliable way?
8	Was the follow-up time reported and sufficient to be long enough for outcomes to occur?
9	Was follow-up complete, and if not, were the reasons for loss to follow-up described and explored?
10	Were strategies to address incomplete follow-up utilized?
11	Was appropriate statistical analysis used?

Table S1b. Appraisal results, cohort studies (n = 29; item-level codes for all 29)

ID	Study (first author/trial, journal, year)	Q1	Q2	Q3	Q4	Q5	Q6	Q7	Q8	Q9	Q10	Q11	Overall
4 [18]	EURAS-IUD 5-yr extension, 2017	Y	Y	Y	Y	Y	Y	Y	Y	N	N	Y	Incl.
19 [19]	APEX-IUD perforation cohort, reported in Anthony 2021	Y	Y	Y	Y	Y	Y	Y	Y	Y	Y	Y	Incl.
20 [93]	APEX-IUD perforation/expulsion (AJOG), 2022	Y	Y	Y	Y	Y	Y	Y	Y	Y	Y	Y	Incl.
30 [12]	EURAS-LCS12 device-shape analysis, 2024	Y	Y	Y	Y	Y	Y	Y	Y	Y	Y	Y	Incl.
36 [17]	EURAS-LCS12 alloy (Ag/Au) analysis, 2026	Y	Y	Y	U	U	Y	Y	Y	U	U	U	Further info
41 [64]	Danish ectopic-pregnancy cohort by LNG dose, 2023	Y	Y	Y	Y	Y	Y	Y	Y	Y	NA	Y	Incl.
53 [94]	Heinemann, Contraception 2017	Y	Y	Y	Y	U	Y	Y	Y	Y	U	Y	Incl.
56 [99]	Siegelmann-Danieli, Breast Cancer Res Treat 2018	Y	Y	Y	N	U	Y	Y	Y	U	U	Y	Incl.
59 [62]	Law, Contraception 2018	U	Y	Y	Y	Y	Y	U	Y	U	Y	Y	Incl.
62 [66]	Graner, Acta Obstet Gynecol Scand 2019	U	U	U	N	N	Y	Y	Y	U	U	U	Incl.
63 [101]	Bosco-Lévy, Contraception 2019	Y	Y	Y	Y	Y	Y	U	Y	Y	Y	Y	Incl.
65 [109]	Lerma, Int J Gynaecol Obstet 2020 †	Y	Y	Y	U	U	Y	Y	Y	U	U	Y	Incl.
71 [126]	Beelen, Eur J Obstet Gynecol Reprod Biol 2021	Y	Y	Y	Y	Y	Y	Y	Y	U	U	Y	Incl.

ID	Study (first author/trial, journal, year)	Q1	Q2	Q3	Q4	Q5	Q6	Q7	Q8	Q9	Q10	Q11	Overall
74 [72]	Gilliam, Contraception 2021	U	Y	Y	Y	Y	Y	Y	Y	U	N	U	Incl.
76 [85]	Akintomide, Eur J Contracept Reprod Health Care 2021	U	Y	Y	Y	N	Y	Y	Y	Y	NA	Y	Incl.
78 [114]	Fay, Contraception 2021	Y	Y	Y	U	U	Y	U	Y	Y	Y	Y	Incl.
80 [115]	BakenRa, Obstet Gynecol 2021	N	Y	U	Y	N	Y	U	Y	Y	Y	Y	Incl.
84 [96]	Armstrong, JAMA Netw Open 2022	Y	Y	Y	Y	Y	Y	Y	Y	Y	Y	Y	Incl.
85 [97]	Getahun, Am J Obstet Gynecol 2022	Y	Y	U	Y	Y	Y	Y	Y	Y	Y	Y	Incl.
86 [65]	Kopp-Kallner, Obstet Gynecol 2022	Y	Y	U	Y	Y	Y	Y	U	Y	Y	Y	Incl.
87 [67]	Elgemark, Obstet Gynecol 2022 †	U	U	U	N	N	Y	Y	Y	U	U	U	Incl.
89 [98]	Anthony, Obstet Gynecol 2022	Y	Y	U	Y	Y	Y	Y	Y	Y	Y	Y	Incl.
92 [69]	Roland, JAMA 2023 †	Y	Y	Y	U	Y	U	U	U	U	U	U	Incl.
102 [95]	Fassett, Obstet Gynecol 2023	Y	Y	Y	Y	Y	Y	Y	Y	Y	Y	Y	Incl.
103 [123]	Kai, Health Technol Assess 2023	Y	Y	Y	U	U	Y	Y	Y	Y	U	Y	Incl.
106 [90]	Brown, Contraception 2024	N	U	U	Y	Y	Y	Y	Y	Y	U	Y	Incl.
107 [68]	Skovlund, Lancet Reg Health Eur 2024	Y	Y	Y	Y	Y	Y	U	Y	Y	Y	Y	Incl.
109 [75]	Kerns, Am J Obstet Gynecol 2024	N	Y	U	Y	Y	U	Y	Y	U	N	Y	Incl.
114 [100]	Hadizadeh, JAMA Oncol 2025	Y	Y	U	Y	Y	Y	Y	Y	Y	Y	Y	Incl.

3. JBI Critical Appraisal Checklist for Case Series (10 items)

Item	Criterion
1	Were there clear criteria for inclusion in the case series?
2	Was the condition measured in a standard, reliable way for all participants included in the case series?
3	Were valid methods used for identification of the condition for all participants included in the case series?
4	Did the case series have consecutive inclusion of participants?
5	Did the case series have complete inclusion of participants?
6	Was there clear reporting of the demographics of the participants in the study?
7	Was there clear reporting of clinical information of the participants?
8	Were the outcomes or follow-up results of cases clearly reported?
9	Was there clear reporting of the presenting site(s)/clinic(s) demographic information?

Item	Criterion
10	Was statistical analysis appropriate?

Table S1c. Appraisal results, single-arm trials and single-group series appraised with the case-series checklist (n = 32; item-level codes for all 32)

ID [ref.]	Study (first author/trial, journal, year)	Q1	Q2	Q3	Q4	Q5	Q6	Q7	Q8	Q9	Q10	Overall
3 [13]	IUB SCu380A pilot study, 2016	Y	Y	Y	U	U	Y	Y	Y	Y	Y	Incl.
6 [77]	Mirena extended-use chart review (Brazil), 2018	Y	Y	Y	U	U	Y	Y	Y	Y	Y	Incl.
8 [86]	Gyn-CS clinical experience (single-arm), 2018	Y	Y	Y	U	U	Y	Y	Y	Y	Y	Incl.
9 [79]	Liletta 5-yr efficacy (ACCESS IUS), 2019	Y	Y	Y	U	Y	Y	Y	Y	Y	Y	Incl.
10 [83]	IUB Ballerine MIDI (Switzerland), 2019	Y	U	Y	U	U	Y	U	Y	Y	Y	Incl.
11 [80]	Liletta 6-yr efficacy (ACCESS IUS), 2020	Y	Y	Y	U	Y	Y	Y	Y	Y	Y	Incl.
12 [84]	IUB Ballerine MIDI (Israel), 2020	Y	U	Y	U	U	Y	U	Y	Y	Y	Incl.
14 [119]	Liletta HMB subgroup analysis, 2020	Y	U	U	U	Y	Y	Y	Y	Y	Y	Incl.
21 [8]	Liletta 8-yr efficacy (ACCESS IUS), reported in Creinin 2023	Y	Y	Y	U	Y	Y	Y	Y	Y	Y	Incl.
22 [9]	Mirena Extension Trial, 8 yr, 2022	Y	Y	Y	U	Y	Y	Y	Y	Y	Y	Incl.
23 [52]	Kyleena Satisfaction study, 2023	Y	Y	Y	U	U	Y	Y	Y	U	Y	Incl.
26 [81]	Liletta beyond 8-yr research letter, 2023	Y	Y	Y	U	U	U	U	Y	Y	Y	Incl.
28 [25]	LNG-IUD for HMB, treatment trial, 2023	Y	Y	Y	U	Y	Y	Y	Y	Y	Y	Incl.
33 [15]	Low-dose Cu IUD, phase 3 (3-yr), 2025	Y	Y	Y	U	Y	Y	Y	Y	Y	Y	Incl.
34 [16]	Low-dose Cu IUD, phase 3 (5-yr), 2026	Y	Y	Y	U	Y	Y	Y	Y	Y	Y	Incl.
45 [55]	Gemzell-Danielsson, Contraception 2016	Y	Y	Y	U	Y	Y	Y	Y	Y	Y	Incl.
48 [73]	Turok, Am J Obstet Gynecol 2016	Y	U	U	U	Y	Y	Y	Y	Y	Y	Incl.
51 [56]	Fan, Contraception 2017	Y	Y	Y	U	Y	Y	Y	Y	Y	Y	Incl.
52 [78]	McNicholas, Am J Obstet Gynecol 2017	Y	U	U	U	U	Y	Y	Y	Y	Y	Incl.
55 [57]	Gemzell-Danielsson, Contraception 2017	Y	U	Y	U	Y	Y	Y	Y	U	Y	Incl.
57 [70]	Darney, Contraception 2018	Y	Y	Y	U	U	Y	Y	Y	Y	Y	Incl.
58 [71]	Schreiber, Eur J Contracept Reprod Health Care 2018	Y	U	Y	U	Y	Y	Y	Y	Y	Y	Incl.
64 [49]	Beckert, Eur J Contracept Reprod Health Care 2019	Y	Y	Y	U	U	Y	Y	Y	U	Y	Incl.
66 [92]	Turok, Obstet Gynecol 2020	Y	Y	Y	U	U	Y	Y	Y	Y	Y	Incl.
67 [51]	Beckert, Eur J Contracept Reprod Health Care 2020	Y	U	Y	U	Y	Y	Y	Y	Y	Y	Incl.
69 [74]	Carr, Contraception 2021	Y	U	N	U	N	Y	Y	Y	Y	Y	Incl.
75 [116]	Boraas, Obstet Gynecol 2021	Y	U	N	U	U	Y	Y	Y	Y	Y	Incl.
81 [53]	Stovall, Eur J Contracept Reprod Health Care 2021	Y	Y	Y	U	U	Y	Y	Y	Y	Y	Incl.
82 [87]	Kutlucan, Life (Basel) 2022	Y	Y	Y	U	U	Y	Y	Y	Y	Y	Incl.
97 [88]	Che, Contraception 2023	Y	U	U	U	Y	Y	Y	Y	Y	Y	Incl.
105 [54]	Römer, Arch Gynecol Obstet 2024	Y	Y	Y	U	U	Y	Y	Y	U	Y	Incl.
111 [117]	Nourse, Contraception 2025	Y	Y	Y	U	Y	Y	Y	Y	Y	Y	Incl.

4. JBI Critical Appraisal Checklist for Quasi-Experimental Studies (9 items)

Item	Criterion
1	Is it clear in the study what is the 'cause' and what is the 'effect' (no ambiguity about the temporal sequence)?
2	Were the participants included in any comparisons similar (or, for one-group pre-post designs, the same participants)?
3	Were the participants included in any comparisons receiving similar treatment/care, other than the exposure or intervention of interest?
4	Was there a control group?
5	Were there multiple measurements of the outcome both pre- and post-intervention/exposure?
6	Was follow-up complete, and if not, were differences between groups in follow-up adequately described and analyzed?
7	Were the outcomes of participants in any comparisons measured in the same way?
8	Were outcomes measured in a reliable way?
9	Was appropriate statistical analysis used?

Table S1d. Appraisal results, quasi-experimental (nonrandomized controlled) study (n = 1)

ID	Study (first author/trial, journal, year)	Q1	Q2	Q3	Q4	Q5	Q6	Q7	Q8	Q9	Overall
42 [28]	LNG-IUD vs dienogest, adenomyosis RCT, 2023	Y	Y	Y	Y	Y	N	Y	Y	N	Incl.

5. Mechanistic, Pharmacokinetic and Modelling Studies (no applicable JBI checklist)

Table S1e. Descriptive characterization of mechanistic and modelling reports (n = 7)

ID [ref.]	Study	Unit of analysis	Method	Main limitation	Use in synthesis
2 [45]	Liletta LNG release-rate study, 2016	100 removed devices (device-level; not an efficacy population)	Residual LNG measured in explanted devices; release rate estimated	Devices removed for any reason; no clinical outcome	Biological plausibility of extended use only
25 [46]	Population PK modeling, 3 LNG-IUD doses, 2023	Pooled pharmacokinetic data from several trials (unique N not stated in the abstract)	Population pharmacokinetic modelling	Model-based estimates dependent on structural assumptions; sponsor-conducted	Comparison of release across LNG doses; not efficacy evidence
37 [82]	Mirena residual-LNG device study, 2026	42 removed devices after >8 years (device-level)	Residual LNG measured in explanted devices	Small convenience sample of removals; no clinical outcome	Plausibility beyond 8 years only
68 [47]	Hofmann, Eur J Contracept Reprod Health Care 2020	Pooled pharmacokinetic data from more than 3,400 participants in 10 studies	Integrated population pharmacokinetic model of six LNG-releasing contraceptives	Sponsor-conducted; model-based estimates	Comparison of systemic LNG exposure across devices and routes
70 [50]	Frenz, Eur J Contracept Reprod Health Care 2021	Bleeding diaries of 1,351 (training) and 1,516 (validation) trial participants	Fixed-cluster and regression-tree prediction models	Trial populations; about 70% correct classification	Counseling context only
73 [103]	Hopelian, BMC Womens Health 2021	Milk samples from 32 women (22 paired) in a randomized trial	Milk LNG concentration and creamatocrit	Self-selected convenience subsample; not powered	Breastfeeding safety context
94 [134]	Hofmann, Clin Pharmacokinet 2023	62 participants with dense sampling within a phase II RCT	Plasma pharmacokinetics and in vivo release of indomethacin and LNG	Proof-of-concept device; short exposure	Feasibility of dual-drug intrauterine release

6. JBI Critical Appraisal Checklist for Case–Control Studies (10 items)

One included report used a case–control design and was appraised with this checklist (Table S1f).

Item	Criterion
1	Were the groups comparable other than the presence of disease in cases or its absence in controls?
2	Were cases and controls matched appropriately?
3	Were the same criteria used for identification of cases and controls?
4	Was exposure measured in a standard, valid and reliable way?
5	Was exposure measured in the same way for cases and controls?
6	Were confounding factors identified?
7	Were strategies to deal with confounding factors stated?
8	Were outcomes assessed in a standard, valid and reliable way for cases and controls?
9	Was the exposure period of interest long enough to be meaningful?
10	Was appropriate statistical analysis used?

Table S1f. Appraisal results, case–control study (n = 1)

ID [ref.]	Study (first author/trial, journal, year)	Q1	Q2	Q3	Q4	Q5	Q6	Q7	Q8	Q9	Q10	Overall
108 [102]	Roland, BMJ 2024	Y	Y	Y	U	Y	Y	U	Y	Y	Y	Incl.

Table S1g. Main appraisal observations for the 68 added reports appraised at item level (58 from the full text and 10 from the abstract only)

ID [ref.]	Study	Tool	Main appraisal observations
43 [127]	Ørbo, BJOG 2016	RCT checklist	Randomization/concealment described only in parent report; follow-up restricted to responders (post-randomization subset); pathologists blinded; no loss to follow-up; underpowered for relapse.
44 [107]	Baldwin, Contraception 2016	RCT checklist	Sealed opaque envelopes from a computer-generated list; open label with phone-reported IUD use; 6-month follow-up 66% in both arms; stopped before the planned sample size.
45 [55]	Gemzell-Danielsson, Contraception 2016	Case-series checklist	Phase 3 single-arm adolescent study at 36 European centers; all 304 with a placement attempt analysed; bleeding-diary data too incomplete to analyse.
46 [120]	Kiseli, Gynecol Obstet Invest 2016	RCT checklist	Abstract only: open three-arm RCT; randomization and concealment not described in the abstract; 62 of 84 randomized evaluated.
47 [58]	Apter, Fertil Steril 2016	RCT checklist	Sponsor computer-generated list distributed in site envelopes (concealment not detailed); open label; Kaplan–Meier discontinuation in the full analysis set (378 of 385 LNG-IUD arm).
48 [73]	Turok, Am J Obstet Gynecol 2016	Case-series checklist	Secondary analysis of ACCESS IUS; pelvic infection diagnosed clinically by study or non-study providers without objective criteria; 1,714 of 1,751 analysed (placement failures excluded).
49 [59]	Borgatta, Eur J Contracept Reprod Health Care 2016	RCT checklist	Abstract only: open-label phase 3 RCT; groups similar in age and parity; satisfaction self-reported; analysis of women with attempted placement or at least one pill.
50 [122]	Kai, Br J Gen Pract 2016	RCT checklist	Central minimized randomization; open label with patient-reported outcome; 74% follow-up at 5 years without comparison of responders; ITT.
51 [56]	Fan, Contraception 2017	Case-series checklist	Phase 3 single-arm Asia-Pacific study at 25 centers; ultrasound-confirmed expulsion; full analysis set of 925; 27.9% discontinued before 3 years with reasons given.
52 [78]	McNicholas, Am J Obstet Gynecol 2017	Case-series checklist	EPIC prospective cohort of extended users; pregnancy largely self-reported with record validation; number of eligible women approached not reported; retention 94.8% at 24 months.

ID [ref.]	Study	Tool	Main appraisal observations
53 [94]	Heinemann, Contraception 2017	Cohort checklist	EURAS-IUD reanalysis restricted to complete perforation; crude and stratified relative risks only; validated follow-up for 60,213 of 61,448.
54 [14]	Reeves, Contraception 2017	RCT checklist	Sequentially numbered opaque envelopes (generation method not stated); participants blinded to device; ultrasound-confirmed outcomes; 2 post-randomization exclusions for failed placement.
55 [57]	Gemzell-Danielsson, Contraception 2017	Case-series checklist	Pooled analysis of three phase 3 studies; pain rated by self-defined categories at an unspecified time; 965 of 971 allocated included; sites described only in parent reports.
56 [99]	Siegelmann-Danieli, Breast Cancer Res Treat 2018	Cohort checklist	HMO pharmacy-purchase exposure; controls age-matched (±2 years) only, no multivariable adjustment; registry-confirmed outcome; loss to follow-up not described.
57 [70]	Darney, Contraception 2018	Case-series checklist	ACCESS IUS secondary analysis; prospective daily diaries (office recall when diaries missing); 1,313 of 1,566 non-prior users analysed at 12 months.
58 [71]	Schreiber, Eur J Contracept Reprod Health Care 2018	Case-series checklist	ACCESS IUS secondary analysis of WHO bleeding patterns; daily diaries for 2 years then 3-month recall; 1,700 of 1,714 with placement analysed; data available for 55% at 3 years.
59 [62]	Law, Contraception 2018	Cohort checklist	US claims cohort; groups differed in prior pregnancy and contraception, Cox-adjusted; discontinuation inferred from claims; only 2,204 of 3,680 users had 12 months of enrollment. Publisher page confirms Contraception 2018;98(2):120-124.
60 [108]	Blumenthal, Contraception 2018	RCT checklist	Computer-generated blocks with sealed opaque envelopes; participants blinded; per-protocol analysis of 480 of 500; no a-priori sample size.
61 [104]	Levi, Breastfeed Med 2018	RCT checklist	Abstract only: secondary analysis of 63 LNG-IUD users from an RCT of intracesarean vs interval placement; breastfeeding self-reported; adjusted proportions.
62 [66]	Graner, Acta Obstet Gynecol Scand 2019	Cohort checklist	Stockholm hospital-based case ascertainment with woman-years estimated from regional sales data; no adjustment for confounders; denominators rest on assumed use duration.
63 [101]	Bosco-Lévy, Contraception 2019	Cohort checklist	French claims sample; new users; propensity-score matching with residual age imbalance adjusted; outcomes from hospital codes and dispensing; as-treated and intent-to-treat analyses.
64 [49]	Beckert, Eur J Contracept Reprod Health Care 2019	Case-series checklist	Sponsor pooled analysis of phase 2 and 3 diary data; predefined handling of missing diary days; selection of the 1,697 analysed users and site details only in parent reports.
65 [109]	Lerma, Int J Gynaecol Obstet 2020	Cohort checklist	Abstract only: secondary analysis of two related Indian trials grouped by delivery-to-insertion interval; confounder handling not reported.
66 [92]	Turok, Obstet Gynecol 2020	Case-series checklist	Sponsor-run phase 2; 286 of 417 screened enrolled; 36% of cycles assumed evaluable after year 1; 37.8% completed 36 months.
67 [51]	Beckert, Eur J Contracept Reprod Health Care 2020	Case-series checklist	KYSS baseline analysis in seven countries (max 20 per site); pain rated by self-defined categories; 1,110 of 1,114 enrolled in the safety set.
69 [74]	Carr, Contraception 2021	Case-series checklist	ACCESS IUS substudy; conception self-reported without confirmation; 53 of 218 women planning pregnancy excluded (15 lost, 2 withdrew, 36 changed plans).
71 [126]	Beelen, Eur J Obstet Gynecol Reprod Biol 2021	Cohort checklist	MIRA preference cohort from the same eligible population; relative risks adjusted for baseline differences; women not receiving the planned treatment excluded; completeness at 24 months not detailed.
72 [106]	Marangoni, Acta Obstet Gynecol Scand 2021	RCT checklist	Computer-generated permuted blocks with sealed opaque envelopes; open label; ultrasound at each visit; 12.8% lost to follow-up at 1 year; Kaplan–Meier analysis.
74 [72]	Gilliam, Contraception 2021	Cohort checklist	ACCESS IUS substudy by parity and delivery route; multivariable logistic model; denominators fall over 6 years without time-to-event analysis or handling of attrition.
75 [116]	Boraas, Obstet Gynecol 2021	Case-series checklist	Pooled from three observational studies and one RCT; 34% reported pregnancy status without a test.
76 [85]	Akintomide, Eur J Contracept Reprod Health Care 2021	Cohort checklist	EURAS-IUD secondary analysis of users <30 y; device-type comparisons bivariate only, no adjustment; 5,762 of 5,796 analysed.
77 [129]	Baxter, Int J Gynecol Cancer 2026 (feMMe trial)	RCT checklist	Central IVRS randomization stratified by diagnosis, BMI, menopause and site; blinded central pathology; pragmatic optional two- and three-arm randomization with several protocol changes; ITT.
78 [114]	Fay, Contraception 2021	Cohort checklist	Post-hoc secondary analysis of randomized arms in a post-randomization subgroup; pregnancy by urine test in most, records/survey in the remainder; sensitivity analysis for missing data.

ID [ref.]	Study	Tool	Main appraisal observations
79 [121]	Ilyin, Eur J Contracept Reprod Health Care 2021	RCT checklist	Abstract only: multicentre noninferiority phase 3 RCT; validated pictogram for blood loss; per-protocol primary analysis (300 of 312).
80 [115]	BakenRa, Obstet Gynecol 2021	Cohort checklist	Arms pooled; single vs multiple intercourse groups differed in age and marital status without adjustment; self-reported intercourse history; 1.8% lost to follow-up.
81 [53]	Stovall, Eur J Contracept Reprod Health Care 2021	Case-series checklist	KYSS 12-month analysis in eight countries; women self-selected the device in routine care; satisfaction available for 968 of 1,126 placements.
82 [87]	Kutlucan, Life (Basel) 2022	Case-series checklist	Single-operator pilot (n = 21); irregular follow-up (three women attended only a late exit visit); no comparator.
83 [105]	Lichtenstein Liljeblad, PhD thesis, Karolinska Institutet 2023	RCT checklist	Sequence generation and concealment not described; open label; expulsion detected clinically without ultrasound; stopped early by prespecified safety rule (101 of 600 planned), primary outcome underpowered.
84 [96]	Armstrong, JAMA Netw Open 2022	Cohort checklist	APEX-IUD EHR cohort; validated exposure/outcome algorithms; propensity-score overlap weighting; censoring described; possible ascertainment bias and residual confounding.
85 [97]	Getahun, Am J Obstet Gynecol 2022	Cohort checklist	APEX-IUD EHR cohort of nonpostpartum insertions; menorrhagia from diagnosis codes; validated outcome algorithms; propensity-score overlap weighting; censoring defined.
86 [65]	Kopp-Kallner, Obstet Gynecol 2022	Cohort checklist	National registers; dispensing used as proxy for use; adjusted for age, endometriosis and prior ectopic only; 13.5-mg device available only from 2014.
87 [67]	Elgemark, Obstet Gynecol 2022	Cohort checklist	Abstract only: Stockholm hospital case ascertainment with woman-years estimated from regional sales data, as in the earlier cohort from this group.
88 [118]	Kaplan, Contraception 2023	RCT checklist	Planned secondary analysis of the EC trial (parent methods as ID 15); participants masked only to 1 month; self-reported switching; 19 expulsions excluded.
89 [98]	Anthony, Obstet Gynecol 2022	Cohort checklist	APEX-IUD; HMB and dysmenorrhea from unvalidated diagnosis codes; parity missing in 18%; mutually adjusted Cox models.
90 [133]	Fels, Am J Obstet Gynecol 2023	RCT checklist	Adaptive phase 2 dose-finding RCT; randomization details only in appendix; participants blinded and bleeding self-recorded in e-diaries; per-protocol analysis (160 of 174).
91 [30]	Gezer, Int J Gynaecol Obstet 2023	RCT checklist	Abstract only: RCT of vaginal progesterone vs LNG-IUD; histological regression; 138 of 144 analysed.
92 [69]	Roland, JAMA 2023	Cohort checklist	Abstract only (research letter): French nationwide matched cohort; dispensing-based exposure and outcomes.
93 [130]	Xu, J Gynecol Oncol 2023	RCT checklist	Random-number tables with concealment; pathologists masked; primary endpoint changed from 3-month to 16-week CR; 54/63 analysed; underpowered phase II.
95 [91]	Bhatla, Int J Gynaecol Obstet 2023	RCT checklist	Abstract only: 600 women randomized equally; 5-year Kaplan–Meier continuation; allocation details and losses not reported in the abstract.
96 [63]	Anjos, Contraception 2023	RCT checklist	Computer-generated blocks stratified by center with opaque sequential envelopes; adolescents rated pain on VAS before learning device type; providers unblinded for ease of insertion.
97 [88]	Che, Contraception 2023	Case-series checklist	14-hospital prospective cohort; ultrasound only at 42 days, later expulsion by telephone report; 463 of 470 followed at least once, 85% at 12 months; life-table analysis.
98 [131]	Zhang, Am J Obstet Gynecol 2023	RCT checklist	Central web-based (REDCap) randomization; open label with self-reported diary outcomes; 2 of 104 LNG-IUS participants excluded from analysis.
99 [112]	Nitesh, Eur J Contracept Reprod Health Care 2023	RCT checklist	Abstract only: RCT of Cu 375 vs CuT 380A after postpartum insertion; ultrasound-confirmed expulsion; modified intention-to-treat analysis.
100 [31]	Xu, Gynecol Oncol 2023	RCT checklist	Random-number-table allocation, concealed until enrolment; hysteroscopists and pathologists blinded; primary endpoint changed from 3-month to 16-week CR; modified ITT of evaluated patients (132 of 180).
101 [111]	Seleem, BMC Pregnancy Childbirth 2023	RCT checklist	Inconsistent description of sequence generation and envelopes; participants blinded; per-protocol analysis (394 per arm); retrospectively registered.
102 [95]	Fassett, Obstet Gynecol 2023	Cohort checklist	Clinical summary of APEX-IUD analyses; methods as in the primary APEX reports.
103 [123]	Kai, Health Technol Assess 2023	Cohort checklist	Randomized ECLIPSE groups followed observationally; 206 of 490 recontactable women responded, responders compared with non-responders; unadjusted analyses; self-report validated against GP records in a sample.
104 [61]	Perelló-Capó, Contraception 2024	RCT checklist	Secondary outcomes of a single-center evaluator-masked phase 4 RCT (randomization in parent report); validated SEC-QoL; analyses restricted to evaluable participants (58 of 106 at 36 months).

ID [ref.]	Study	Tool	Main appraisal observations
105 [54]	Römer, Arch Gynecol Obstet 2024	Case-series checklist	National subset of KYSS; sponsor-funded; satisfaction missing for 12.5%.
106 [90]	Brown, Contraception 2024	Cohort checklist	Cup users differed in ethnicity, BMI and parity; product use not recorded at baseline or for 19.3% of early visits; adjusted logistic model.
107 [68]	Skovlund, Lancet Reg Health Eur 2024	Cohort checklist	New-user nationwide register design; antidepressant redemption as proxy for depression; standardised and multiple sensitivity analyses; residual confounding by indication possible.
108 [102]	Roland, BMJ 2024	Case–control checklist	Risk-set matched on birth year and area; exposure from dispensing within 3-5 years; confounding addressed through matching and high-dose progestogen exposure modes; copper IUD negative control.
109 [75]	Kerns, Am J Obstet Gynecol 2024	Cohort checklist	ACCESS IUS new users grouped by prior method (combined hormonal vs none); groups differed at baseline, multivariable adjustment; no handling of incomplete follow-up.
110 [128]	Goh, J Assist Reprod Genet 2024	RCT checklist	Sealed opaque envelopes, generation method not described; open label; 40 of 60 planned randomized; non-inferiority claimed although underpowered; retrospectively registered on ClinicalTrials.gov.
111 [117]	Nourse, Contraception 2025	Case-series checklist	Secondary analysis of the EC trial; expected pregnancies modelled from cycle-day probabilities derived from a conception-seeking cohort; 95-97% of randomized included.
112 [110]	Bento Herculano, Int J Gynaecol Obstet 2026	RCT checklist	Computer block randomization and sealed envelopes; participants blinded; differential loss to follow-up (2% vs 13.4%) not analysed; patient-reported complete expulsion without ultrasound.
113 [132]	Yan, Am J Obstet Gynecol 2026	RCT checklist	36-month extension of the niche RCT; randomization as in parent trial; co-treatments allowed and not reported by arm; 11 and 15 missing at 36 months; ITT with GEE.
114 [100]	Hadizadeh, JAMA Oncol 2025	Cohort checklist	Nationwide registers; redeemed prescriptions as exposure; DAG-guided adjustment and quantitative bias analysis for unmeasured confounders.

References

1. Peters MDJ, Godfrey C, McInerney P, Munn Z, Tricco AC, Khalil H. Chapter 11: Scoping reviews. In: Aromataris E, Munn Z, eds. JBI Manual for Evidence Synthesis. JBI; 2020. doi:10.46658/JBIMES-20-12. Critical appraisal checklists adapted from: Tufanaru C, Munn Z, Aromataris E, Campbell J, Hopp L. Chapter 3: Systematic reviews of effectiveness. In: Aromataris E, Munn Z, eds. JBI Manual for Evidence Synthesis. JBI; 2020; and Moola S, Munn Z, Tufanaru C, et al. Chapter 7: Systematic reviews of etiology and risk. In: Aromataris E, Munn Z, eds. JBI Manual for Evidence Synthesis. JBI; 2020. © JBI. Checklists used for research purposes under JBI's terms of use.

2. Munn Z, Barker TH, Moola S, et al. Methodological quality of case series studies: an introduction to the JBI critical appraisal tool. JBI Evid Synth. 2020;18(10):2127-2133. doi:10.11124/JBISRIR-D-19-00099.

Table S2. Characteristics and evidence map of the 114 included publications

IDs are internal identifiers used in Supplementary Tables S1 and S2; the manuscript cites each report by the reference number given in brackets after its ID. DOI = digital object identifier; PMID = PubMed identifier. All included studies were published in journals indexed in the Science Citation Index Expanded (SCIE). For six trials (IDs 1, 19, 21, 38, 77 and 83), the cited reference is a later report, review or thesis covering the same trial; the design, tool and appraisal shown refer to the trial itself. The 'Why relevant' column for these reports gives the scope criterion met (Methods 2.4). PubMed and DOI hyperlink columns are likewise omitted for width;

ID	Theme	Article	Journal	Study type	N / analysis population	Device(s)	Year	DOI	PMID	Underlying cohort / overlap	Why relevant to review	Principal quantitative / clinical finding
1 [76]	Extended duration / comparative efficacy	Effectiveness and safety of extending intrauterine device duration: a systematic review	American Journal of Obstetrics & Gynecology	Open-label 7-year randomized controlled comparative trial	3,755 analyzed (1,884 LNG-IUD; 1,871 TCu380A)	52-mg LNG-IUD vs TCu380A	2020	10.1016/j.ajog.2020.01.014	31954154	WHO multicenter randomized cohort	Key long-duration head-to-head evidence for hormonal vs copper IUD	7-y cumulative pregnancy 0.53/100 with 52-mg LNG-IUD vs 2.45/100 with TCu380A.
2 [45]	Hormonal device pharmacology	Levonorgestrel release rates over 5 years with the Liletta® 52-mg intrauterine system	Contraception	Device-content / release-rate study nested in phase 3 program	100 removed devices analyzed (device-level; not an efficacy N)	Liletta 52-mg LNG-IUD	2016	10.1016/j.contraception.2016.04.010	27125892	ACCESS IUS / Liletta phase III program	Mechanistic evidence supporting prolonged LNG release and later duration extensions	Estimated LNG release declined from 19.5 µg/day initially to 9.8 µg/day at 5 y.
3 [13]	Novel copper geometry	Discontinuation rates and acceptability during 1 year of using the intrauterine ball (the SCu380A)	Contraception	Prospective pilot study	51	SCu380A intrauterine ball (IUB)	2016	10.1016/j.contraception.2015.12.002	26680758	Independent pilot cohort	Important negative early evidence for spherical copper-IUD design	At 12 mo, 27% expelled, 16% removed for symptoms, and 1 pregnancy occurred.
4 [18]	Long-term safety	Perforation risk and intra-uterine devices: results of the EURAS-IUD 5-year extension study	European Journal of Contraception & Reproductive Health Care	Prospective non-interventional cohort; 5-year extension	61,448 original cohort; 39,009 with 60-month follow-up	LNG-IUD and copper IUDs	2017	10.1080/13625187.2017.1412427	29322856	EURAS-IUD	Large real-world long-term perforation evidence	5-y perforation ≈2.1/1000 LNG-IUD vs 1.6/1000 copper; adjusted estimate ≈1.7.
5 [20]	Postpartum use	Immediate postpartum levonorgestrel intrauterine device insertion and breast-feeding outcomes: a noninferiority randomized controlled trial	American Journal of Obstetrics & Gynecology	Noninferiority randomized controlled trial	319 enrolled; 259 ITT analyzed (132 immediate; 127 delayed)	LNG-IUD: immediate postpartum vs delayed insertion	2017	10.1016/j.ajog.2017.08.003	28842126	Postpartum LNG-IUD RCT	Supports expansion of timing of insertion into the immediate postpartum period	Breastfeeding outcome noninferior; expulsion 19% immediate vs 2% delayed postpartum.
6 [77]	Extended duration	Pregnancy outcomes associated with extended use of the 52-mg 20 µg/day levonorgestrel-releasing intrauterine system beyond 60 months: A chart review of 776 women in Brazil	Contraception	Retrospective chart review	766 assessed (title reports 776 identified)	Mirena 52-mg LNG-IUD	2018	10.1016/j.contraception.2017.10.007	29055780	University of Campinas extended-use cohort	Early real-world evidence supporting use beyond 5 years	No pregnancies among 776 women continuing the same 52-mg LNG-IUD beyond 5 y; 1,485 woman-years through 15 y, but highly selected long-term cohort.
7 [11]	Frameless / postpartum copper	Comparison of expulsions following intracesarean placement of an innovative frameless copper-releasing IUD (Gyn-CS®) versus the TCu380A: A randomized trial	Contraception	Randomized controlled trial	140 (70/70)	Gyn-CS frameless copper vs TCu380A	2018	10.1016/j.contraception.2018.03.034	29678365	Gyn-CS intracesarean trial	Tests frameless anchoring as a design solution to postpartum expulsion	Intracesarean expulsion 1.4% Gyn-CS vs 11.4% TCu380A at 3 mo (P=.039).
8 [86]	Frameless / postpartum copper	Clinical experience with a novel anchored, frameless copper-releasing contraceptive device for intra-caesarean insertion	European Journal of Contraception & Reproductive Health Care	Prospective nonrandomized clinical study	100	Gyn-CS frameless copper IUD	2018	10.1080/13625187.2018.1491027	30273057	Gyn-CS clinical cohort	Additional clinical evidence for anchored frameless postpartum design	One expulsion among 100 Gyn-CS insertions; no serious adverse events at ≈90 d.
9 [79]	Extended duration	Five-Year Contraceptive Efficacy and Safety of a Levonorgestrel 52-mg Intrauterine System	Obstetrics & Gynecology	Phase 3 multicenter clinical trial	1,751 enrolled; 1,714 successful insertion	Liletta 52-mg LNG-IUD	2019	10.1097/AOG.0000000000003034	30531565	ACCESS IUS / NCT00995150	Five-year milestone in the cohort later followed to 6, 8, 9 and 10 years	5-y life-table pregnancy 0.92% for Liletta/ACCESS IUS.
10 [83]	Novel copper geometry	Real-world experience with the IUB Ballerine MIDI copper IUD: an observational study in the French-speaking region of Switzerland	European Journal of Contraception & Reproductive Health Care	Retrospective observational study	207	IUB Ballerine MIDI copper IUD	2019	10.1080/13625187.2019.1618447	31169412	IUB real-world Swiss cohort	Real-world evidence on spherical/3D copper-IUD design	Swiss Ballerine cohort: expulsion 5.3%, pregnancy 1.4%; observational.
11 [80]	Extended duration	Six-year contraceptive efficacy and continued safety of a levonorgestrel 52 mg intrauterine system	Contraception	Ongoing phase 3 multicenter follow-up	1,751	Liletta 52-mg LNG-IUD	2020	10.1016/j.contraception.2019.10.010	31786203	ACCESS IUS / NCT00995150; overlaps Teal 2019 and Creinin 2023	Six-year efficacy/safety milestone	Six-year ACCESS IUS: 9 pregnancies over 6 y; life-table pregnancy 0.87%; none in y6.
12 [84]	Novel copper geometry	Real-world experience with the IUB Ballerine MIDI copper IUD: an observational, single-centre study in Israel	European Journal of Contraception & Reproductive Health Care	Retrospective observational study	175	IUB Ballerine MIDI copper IUD	2020	10.1080/13625187.2019.1699048	31852282	IUB real-world Israeli cohort	Independent real-world experience with a non-T-shaped copper system	Israeli Ballerine cohort: expulsion 3.4%, pregnancy 0.57%; observational.

ID	Theme	Article	Journal	Study type	N / analysis population	Device(s)	Year	DOI	PMID	Underlying cohort / overlap	Why relevant to review	Principal quantitative / clinical finding
13 [21]	Postpartum use	Postplacental placement of intrauterine devices: A randomized clinical trial	Contraception	Randomized controlled trial	140 (70 TCu380A; 70 LNG-IUD)	TCu380A vs LNG 52-mg IUS, postplacental	2020	10.1016/j.contraception.2019.12.006	31927027	Campinas postpartum trial	Direct comparison of copper and LNG systems when inserted postplacentally	90-d expulsion 36.7% TCu380A vs 20.0% LNG-IUD; vaginal delivery was a stronger predictor than device type.
14 [119]	Heavy menstrual bleeding	Bleeding changes after levonorgestrel 52-mg intrauterine system insertion for contraception in women with self-reported heavy menstrual bleeding	American Journal of Obstetrics & Gynecology	Prospective cohort/subgroup analysis	1,513 evaluable overall; 150 self-reported HMB subgroup	Liletta 52-mg LNG-IUD	2020	10.1016/j.ajog.2019.11.1288	31866516	ACCESS IUS / NCT00995150; overlaps Liletta duration studies	Demonstrates therapeutic bleeding effects in real-world HMB phenotype	Self-reported HMB resolved in 74.7% after cycle 1 and 97.1% by cycle 26.
15 [24]	Emergency contraception	Levonorgestrel vs. Copper Intrauterine Devices for Emergency Contraception	New England Journal of Medicine	Randomized noninferiority trial	711 randomized; primary outcome data in 317 LNG and 321 copper users	LNG 52-mg IUD vs copper T380A	2021	10.1056/NEJMoa2022141	33503342	NCT02175030; same cohort as Kaiser 2023	Practice-changing evidence expanding emergency-contraception options beyond copper	Emergency contraception: 1-mo pregnancy 0.3% LNG 52 mg vs 0% copper; noninferior.
16 [32]	Next-generation drug delivery	Pharmacodynamics and pharmacokinetics of a copper intrauterine contraceptive system releasing ulipristal acetate: A randomized proof-of-concept study	Contraception	Single-blind randomized proof-of-concept study	29 randomized; 27 completed	Copper IUS releasing ulipristal acetate (5/20/40 µg/day)	2021	10.1016/j.contraception.2021.06.010	34157312	Novel Cu-UPA proof-of-concept cohort	Demonstrates IUD as a platform for combined local drug delivery and copper contraception	Bleeding-only days decreased dose-dependently with Cu-UPA; −77% at 40 µg/day; proof-of-concept.
17 [26]	Heavy menstrual bleeding	Levonorgestrel-releasing intrauterine system versus endometrial ablation for heavy menstrual bleeding	American Journal of Obstetrics & Gynecology	Multicenter randomized controlled trial	270 randomized (132 LNG-IUD; 138 ablation)	52-mg LNG-IUD vs radiofrequency endometrial ablation	2021	10.1016/j.ajog.2020.08.016	32795428	MIRA trial; same cohort as Huijs 2024 long-term follow-up	Defines therapeutic role of LNG-IUD versus procedural treatment	At 24 mo, surgical reintervention 27% LNG-IUD-first vs 10% ablation; both reduced bleeding.
18 [10]	Smaller / uterine-fit copper devices	Continuation rates of two different-sized copper intrauterine devices among nulliparous women: Interim 12-month results of a single-blind, randomised, multicentre trial	EClinicalMedicine	Participant-blinded randomized multicenter trial	927 randomized (744 NTCu380-Mini; 183 TCu380A)	NTCu380-Mini vs TCu380A	2022	10.1016/j.eclinm.2022.101554	35865736	Mini-copper randomized cohort; overlaps Hubacher 2026 and excluded Schreiber 2025 primary report	Key evidence for cavity/device-size matching in nulliparous users	12-mo continuation 78.7% mini vs 70.2% TCu380A; expulsion 4.8% vs 8.9%.
19 [19]	Large-scale safety	Design of the Association of Uterine Perforation and Expulsion of Intrauterine Device study: a multisite retrospective cohort study	American Journal of Obstetrics & Gynecology	Large multisite retrospective cohort	326,658	LNG-IUDs and copper IUDs	2021	10.1016/j.ajog.2021.01.003	33460585	APEX-IUD; overlaps Gatz 2022	Landmark contemporary estimate of perforation incidence and risk factors	APEX-IUD: 5-y perforation 0.61% overall, 0.29% nonpostpartum; highest relative risk 4 d–≤6 wk postpartum.
20 [93]	Large-scale safety	Association between intrauterine device type and risk of perforation and device expulsion: results from the Association of Perforation and Expulsion of Intrauterine Device study	American Journal of Obstetrics & Gynecology	Retrospective cohort	322,898	LNG-IUD vs copper IUD	2022	10.1016/j.ajog.2022.03.062	35395215	APEX-IUD; substantial overlap with Anthony 2021	Direct modern comparison of device type for perforation and expulsion	LNG vs copper: 5-y perforation 0.63% vs 0.55%; expulsion 4.52% vs 4.82%.
21 [8]	Extended duration	Extending use of levonorgestrel 52 mg intrauterine device to 8 years	American Journal of Obstetrics & Gynecology	Phase 3 multicenter extended follow-up	1,751 enrolled; 569 began year 7; 343 completed year 8	Liletta 52-mg LNG-IUD	2023	10.1016/j.ajog.2023.03.011	36933690	ACCESS IUS / NCT00995150	Central evidence supporting extension of 52-mg LNG-IUD contraception to 8 years	ACCESS IUS through 8 y: cumulative life-table pregnancy 1.32%; no pregnancy in y8.
22 [9]	Extended duration	Contraceptive efficacy and safety of the 52-mg levonorgestrel intrauterine system for up to 8 years: findings from the Mirena Extension Trial	American Journal of Obstetrics & Gynecology	Multicenter single-arm extension study	362 starting year 6; 223 completed 8 years	Mirena 52-mg LNG-IUD	2022	10.1016/j.ajog.2022.09.007	36096186	Mirena Extension Trial / NCT02985541	Independent 8-year Mirena efficacy and safety evidence	Mirena years 6–8: Pearl Index 0.28; cumulative failure 0.68%; high satisfaction among completers.
23 [52]	Lower-dose hormonal IUD	Bleeding profile satisfaction and pain and ease of placement with levonorgestrel 19.5 mg IUD: findings from the Kyleena® Satisfaction study	European Journal of Contraception & Reproductive Health Care	Prospective multinational observational study	1,129	Kyleena 19.5-mg LNG-IUD	2023	10.1080/13625187.2022.2136939	36342694	Kyleena Satisfaction study	Real-world evidence for a smaller/lower-dose hormonal IUD, including nulliparous users	Kyleena real-world study: 77.8% no/mild placement pain; 91.1% of placements rated easy.

ID	Theme	Article	Journal	Study type	N / analysis population	Device(s)	Year	DOI	PMID	Underlying cohort / overlap	Why relevant to review	Principal quantitative / clinical finding
24 [60]	Lower-dose hormonal IUD	Bleeding profile and safety of a levonorgestrel 13.5 mg intrauterine device versus Nova T copper 380 mm2 intrauterine device: Results of a 3-year, single-center, randomized phase 4 study	Contraception	Randomized phase 4 clinical trial	106 (55 LNG 13.5 mg; 51 copper)	LNG 13.5-mg IUD vs Nova T Cu380	2023	10.1016/j.contraception.2023.110127	37487868	Single-center phase 4 cohort	Provides comparative bleeding/safety data for the lowest-dose LNG-IUD	At 36 mo, LNG 13.5 mg had 4 vs 15 bleeding days/90 d, PBAC 7.9 vs 126, and amenorrhea 40% vs 0% vs copper.
25 [46]	Hormonal device pharmacology	Extended use of levonorgestrel-releasing intrauterine system (LNG-IUS) 52 mg: A population pharmacokinetic approach to estimate in vivo levonorgestrel release rates and systemic exposure including comparison with two other LNG-IUSs	Contraception	Population pharmacokinetic modeling using pooled clinical-trial data	Pooled MET + earlier clinical trials; exact unique N not stated in abstract	Mirena 52 mg vs Kyleena 19.5 mg vs Jaydess/Skyla 13.5 mg	2023	10.1016/j.contraception.2023.109954	36634730	Uses Mirena Extension Trial plus prior PK datasets	Explains dose/exposure differences and biological plausibility of extended use	Modeled Mirena LNG release ≈7.0 µg/day at 8 y, comparable to 19.5-mg system at 5 y.
26 [81]	Extended duration beyond 8 years	Levonorgestrel 52-mg Intrauterine Device Efficacy and Safety After More Than 8 Years of Use	Obstetrics & Gynecology	Extended phase 3 follow-up / research letter	339 users >8 years; 83 reached 9 years; 77 reached 10 years	Liletta 52-mg LNG-IUD	2023	10.1097/AOG.0000000000005147	37023451	ACCESS IUS / NCT00995150	Exploratory evidence for efficacy and safety during years 9–10	Among 339 users >8 y, no pregnancies in years 9–10; only 83 reached y9 and 77 y10.
27 [113]	Emergency contraception	One-year pregnancy and continuation rates after placement of levonorgestrel or copper intrauterine devices for emergency contraception: a randomized controlled trial	American Journal of Obstetrics & Gynecology	Randomized-trial follow-up	655 analyzed (327 LNG; 328 copper)	LNG 52-mg IUD vs copper T380A	2023	10.1016/j.ajog.2022.11.1296	36427600	NCT02175030; follow-up of Turok 2021 NEJM trial	Shows one-year continuation and pregnancy outcomes after emergency-contraception placement	At 1 y after EC placement: pregnancy 2.8% LNG vs 3.0% copper; continuation 62.4% vs 55.8%.
28 [25]	Heavy menstrual bleeding	Heavy Menstrual Bleeding Treatment With a Levonorgestrel 52-mg Intrauterine Device	Obstetrics & Gynecology	Prospective multicenter treatment trial	105 enrolled; 89 with evaluable follow-up	52-mg LNG-IUD	2023	10.1097/AOG.0000000000005137	37023455	Dedicated HMB treatment cohort	Contemporary dedicated efficacy evidence for therapeutic LNG-IUD use	Objective HMB trial: median blood-loss reduction 97.6% by cycle 6; conservative success 81.8%.
29 [124]	Heavy menstrual bleeding	A randomized trial comparing the 52-mg levonorgestrel system with combination oral contraceptives for treatment of heavy menstrual bleeding	American Journal of Obstetrics & Gynecology	Pragmatic randomized trial	62 randomized (29 LNG-IUD; 33 COC)	52-mg LNG-IUD vs combined oral contraceptives	2023	10.1016/j.ajog.2023.07.049	37536486	Independent pragmatic HMB trial	Comparative-effectiveness evidence versus another common hormonal treatment	LNG-IUD vs COC HMB trial: no significant ITT difference at 6 or 12 mo; per-protocol benefit at 6 mo.
30 [12]	Copper device geometry	Does the shape of the copper intrauterine device play a role in expulsion? Results from the ongoing European Active Surveillance Study on LCS12	Contraception	Secondary analysis of prospective non-interventional cohort	26,381 copper-IUD users	Nova-T, Tatum-T, Multiload, frameless IUDs, intrauterine balls	2024	10.1016/j.contraception.2024.110444	38552821	EURAS-LCS12; overlaps Boehnke 2026 alloy analysis	Large real-world evidence that device geometry is clinically relevant to expulsion risk	EURAS-LCS12: geometry associated with expulsion; IUB aHR 3.6 vs Tatum-T reference.
31 [125]	Heavy menstrual bleeding	A 52-mg levonorgestrel-releasing intrauterine system vs bipolar radiofrequency nonresectoscopic endometrial ablation in women with heavy menstrual bleeding: long-term follow-up of a multicenter randomized controlled trial	American Journal of Obstetrics & Gynecology	Long-term follow-up of multicenter RCT	196 long-term participants from 270 originally randomized	52-mg LNG-IUD vs radiofrequency endometrial ablation	2024	10.1016/j.ajog.2024.01.016	38280433	MIRA trial; same cohort as Beelen 2021	7.4-year mean follow-up clarifies durability of therapeutic IUD strategy	Mean 7.4-y MIRA follow-up: surgical reintervention 35.3% LNG-IUD-first vs 19.1% ablation; hysterectomy not significantly different.
32 [27]	Endometrial disease / therapeutic expansion	The efficacy of the levonorgestrel intrauterine system versus oral megestrol acetate in treating atypical endometrial hyperplasia: a superior randomized controlled trial	Journal of Gynecologic Oncology	Single-center open-label randomized controlled trial	148	LNG-IUD vs oral megestrol acetate	2024	10.3802/jgo.2024.35.e62	38425141	Independent AEH trial	Shows expansion of intrauterine progestin delivery into conservative treatment of premalignant endometrial disease	AEH: 12-mo complete regression 91.9% LNG-IUD vs 77.0% megestrol; faster regression and less weight gain.
33 [15]	Low-dose flexible copper	Three-year efficacy, safety, and tolerability outcomes from a phase 3 study of a low-dose copper intrauterine device	Contraception	Phase 3 multicenter single-arm trial	1,620 enrolled; 1,601 successful placement	Cu 175 mm² flexible nitinol-frame IUD (Miudella/VeraCept development)	2025	10.1016/j.contraception.2024.110771	39581486	NCT03633799; same cohort as Gawron 2026	Major next-generation copper-IUD development: lower copper load + flexible nitinol frame	Cu 175 mm²: placement 98.8%; 3-y pregnancy 2.47%, expulsion 3.9%; single-arm phase III.
34 [16]	Low-dose flexible copper	Five-Year Phase 3 Efficacy and Safety Outcomes With a Low-Dose Copper Intrauterine Device	Obstetrics & Gynecology	Phase 3 multicenter extended follow-up	1,620 enrolled; 1,601 successful placement; 589 completed 5 years	Cu 175 mm² flexible nitinol-frame IUD	2026	10.1097/AOG.0000000000006227	41747268	NCT03633799; same cohort as Creinin 2025	Extends clinical evidence for the new low-dose flexible copper device to 5 years	Cu 175 mm² at 5 y: cumulative Pearl Index 1.02; life-table pregnancy 4.1%; expulsion 4.7%.

ID	Theme	Article	Journal	Study type	N / analysis population	Device(s)	Year	DOI	PMID	Underlying cohort / overlap	Why relevant to review	Principal quantitative / clinical finding
35 [89]	Smaller / uterine-fit copper devices	Comparing the TCu380A and a small Nova-T copper IUD: secondary analysis of discontinuation probabilities, bleeding outcomes and satisfaction in nulliparous participants from a 3-year randomized trial	Contraception	Secondary analysis of randomized multicenter trial	927 (744 NTCu380-Mini; 183 TCu380A)	NTCu380-Mini vs TCu380A	2026	10.1016/j.contraception.2026.111571	42537769	Same mini-copper cohort as Hubacher 2022 and excluded Schreiber 2025	Three-year evidence on discontinuation, bleeding, pain, expulsion and satisfaction by device size	At 3 y, mini copper had less bleeding/pain discontinuation (14.5% vs 26.8%) and expulsion (10.0% vs 15.1%) than TCu380A.
36 [17]	Copper materials	Polished but pointless? Assessing the potential of gold and silver-alloy IUDs versus copper-only devices in a large multinational cohort (EURAS-LCS12)	European Journal of Contraception & Reproductive Health Care	Exploratory cohort analysis	28,280 copper-IUD users	Copper-only vs copper-silver vs copper-gold IUDs	2026	10.1080/13625187.2026.2616357	41587081	EURAS-LCS12; overlaps 2024 geometry analysis	Directly evaluates whether alloy additions provide measurable clinical benefit	Pregnancy and PID similar across copper-only, copper-silver and copper-gold devices; technical complaints highest with gold alloy.
37 [82]	Extended duration beyond 8 years	Quantification of residual levonorgestrel following extended use of the levonorgestrel 52 mg intrauterine device beyond 8 years	Contraception	Prospective device-content study	42 removed IUDs analyzed (device-level)	Mirena 52-mg LNG-IUD	2026	10.1016/j.contraception.2026.111546	42365916	University of Campinas extended-use device cohort	Mechanistic evidence on residual LNG after ≥8 years; useful for future-duration discussion	Residual Mirena LNG persisted beyond 8 y; median residual content ≈11.2 mg at 10 y; mechanistic only.
38 [48]	Lower-dose hormonal IUD	Levonorgestrel-releasing intrauterine system (LNG-IUS 12) for prevention of pregnancy for up to five years	Expert Review of Clinical Pharmacology	Phase III randomized clinical trial with 5-year extension of the 19.5-mg system	2,885 randomized overall; 1,453 assigned to the 19.5-mg system; 550 completed 5 years	LNG-IUD 19.5 mg (Kyleena precursor / LNG-IUS 12)	2017	10.1080/17512433.2017.1341308	28617060	Bayer phase III low-dose LNG-IUD program	Established 5-year efficacy, bleeding profile, amenorrhea, expulsion, perforation and PID estimates for the 19.5-mg LNG-IUD	19.5-mg LNG-IUD: cumulative 5-y Pearl Index 0.29; failure 1.4%; expulsion 3.7%; amenorrhea 22.6% at y5.
39 [23]	Adolescents / special populations	Clinical Assessment of 3 Intrauterine Devices in Adolescent Girls: A Randomized Clinical Trial	Journal of Pediatric and Adolescent Gynecology	Randomized clinical trial	318 adolescents randomized; 317 in discontinuation analysis	TCu380A vs LNG-IUD 52 mg vs LNG-IUD 19.5 mg	2024	10.1016/j.jpag.2023.12.002	38113970	Independent two-center Brazilian adolescent RCT	Direct adolescent comparison of continuation, expulsion, bleeding, dysmenorrhea and satisfaction across copper and two LNG-IUD doses	Adolescents: 1-y continuation 75.4 TCu380A vs 88.6 LNG52 vs 86.8 LNG19.5 per 100 WY; copper had more expulsion/bleeding-pain removal.
40 [22]	Postpartum use	Early vs Interval Postpartum Intrauterine Device Placement: A Randomized Clinical Trial	JAMA	Randomized noninferiority clinical trial	404 randomized; 294 received an IUD and completed 6-month assessment	Early postpartum IUD placement (2–4 weeks) vs interval placement (6–8 weeks)	2023	10.1001/jama.2023.1936	36943214	NCT03462758	Tested whether early postpartum insertion preserves retention while improving access relative to standard interval placement	Complete expulsion 2.0% at 2–4 wk vs 0% at 6–8 wk postpartum; early placement met noninferiority margin.
41 [64]	Lower-dose hormonal IUD / large-scale safety	Ectopic Pregnancy Risk in Users of Levonorgestrel-Releasing Intrauterine Systems With 52, 19.5, and 13.5 mg of Hormone	JAMA	Nationwide register-based cohort study / research letter	963,964 women; 7.8 million person-years	LNG-IUD 52 mg vs 19.5 mg vs 13.5 mg; other hormonal contraception reference	2023	10.1001/jama.2023.0380	36943224	Danish nationwide register cohort	Large-scale dose-specific ectopic-pregnancy analysis; the 13.5-mg system had the highest adjusted absolute and relative rate	Ectopic pregnancy 7.7, 7.1 and 15.7/10,000 person-years for 52-, 19.5- and 13.5-mg LNG-IUD; 13.5 mg highest.
42 [28]	Adenomyosis / therapeutic expansion	Compare the Efficacy of Dienogest and the Levonorgestrel Intrauterine System in Women with Adenomyosis	Clinical Therapeutics	Comparative interventional study described as randomized; assignment by odd/even first-visit date	117 enrolled	LNG-IUD vs dienogest	2023	10.1016/j.clinthera.2023.07.019	37599165	Single-center adenomyosis cohort	Compared pain and uterine-volume outcomes; interpretation limited by predictable allocation and missing-data handling	Both LNG-IUD and dienogest improved adenomyosis symptoms; dienogest showed lower pain/better uterine-volume control; 8/48 LNG-IUD expulsions; pseudo-random allocation.
43 [127]	Therapeutic / local drug delivery	Relapse risk of endometrial hyperplasia after treatment with the levonorgestrel-impregnated intrauterine system or oral progestogens.	BJOG	Multicentre RCT; 24-month post-treatment follow-up	153 treated; 135 complete responders followed	52-mg LNG-IUD vs two oral MPA regimens	2016	10.1111/1471-0528.13763	26630538	Norwegian EH trial	Scope (b): randomized trial in a mapped domain	Relapse of non-atypical endometrial hyperplasia within 24 months in 41% of complete responders, similar across LNG-IUD and MPA arms (P=0.66).
44 [107]	Postpartum timing / technique	Intrauterine device placement at 3 versus 6 weeks postpartum: a randomized trial.	Contraception	Randomized controlled trial	201 (101 early; 100 standard)	IUD (any) at 3 vs 6 weeks postpartum	2016	10.1016/j.contraception.2015.12.006	26686914	Oregon early-postpartum trial	Scope (b): randomized trial in a mapped domain	IUD use at 3 months 73.0% (3 weeks) vs 75.3% (6 weeks); no difference in insertion pain or satisfaction.

ID	Theme	Article	Journal	Study type	N / analysis population	Device(s)	Year	DOI	PMID	Underlying cohort / overlap	Why relevant to review	Principal quantitative / clinical finding
45 [55]	Adolescents	A Phase III, single-arm study of LNG-IUS 8, a low-dose levonorgestrel intrauterine contraceptive system (total content 13.5mg) in postmenarcheal adolescents.	Contraception	Phase III single-arm trial	304 adolescents (12–17 y)	13.5-mg LNG-IUD (LNG-IUS 8)	2016	10.1016/j.contraception.2016.02.004	26872720	LNG-IUS 8 phase III programme (adolescent study)	Scope (a): report from a named device or trial program	Placement 99.7%; no pregnancies, PID, ectopic pregnancy or perforation in 12 months; satisfaction 83.9%; 16.8% discontinued.
46 [120]	Therapeutic / local drug delivery	Comparison of Therapeutic Efficacies of Norethisterone, Tranexamic Acid and Levonorgestrel-Releasing Intrauterine System for the Treatment of Heavy Menstrual Bleeding: A Randomized Controlled Study.	Gynecol Obstet Invest	Randomized controlled trial	84 randomized; 62 evaluated	52-mg LNG-IUD vs norethisterone vs tranexamic acid	2016	10.1159/000443393	26950475	Independent HMB trial	Scope (b): randomized trial in a mapped domain	6-month reduction in menstrual blood loss 85.8% with LNG-IUD vs 53.1% (norethisterone) and 60.8% (tranexamic acid).
47 [58]	Hormonal IUD: dose, duration, pharmacology	A 12-month multicenter, randomized study comparing the levonorgestrel intrauterine system with the etonogestrel subdermal implant.	Fertil Steril	Randomized open-label phase III trial	766	13.5-mg LNG-IUD vs etonogestrel implant	2016	10.1016/j.fertnstert.2016.02.036	27016644	LNG-IUS 8 phase III programme (implant comparison)	Scope (b): randomized trial in a mapped domain	12-month discontinuation 19.6% vs 26.8% (difference −7.2%); fewer bleeding-related discontinuations with the LNG-IUD.
48 [73]	Population-level safety	A prospective assessment of pelvic infection risk following same-day sexually transmitted infection testing and levonorgestrel intrauterine system placement.	Am J Obstet Gynecol	Prospective analysis within single-arm phase 3 trial	1,751	52-mg LNG-IUD (Liletta)	2016	10.1016/j.ajog.2016.05.017	27180886	ACCESS IUS / NCT00995150	Scope (a): report from a named device or trial program	Same-day STI testing and placement: pelvic infection in 0.5% over 2 years; none among 23 with positive results unknown at placement.
49 [59]	Hormonal IUD: dose, duration, pharmacology	A multicentre, open-label, randomised phase III study comparing a new levonorgestrel intrauterine contraceptive system (LNG-IUS 8) with combined oral contraception in young women of reproductive age.	Eur J Contracept Reprod Health Care	Randomized open-label phase III trial	560 (279 LNG-IUD; 281 COC)	13.5-mg LNG-IUD vs EE/drospirenone COC	2016	10.1080/13625187.2016.1212987	27494570	LNG-IUS 8 phase III programme (COC comparison)	Scope (b): randomized trial in a mapped domain	Satisfaction at 18 months 82.1% vs 81.9%; preference to continue 66.2% vs 48.8% (P=0.0001).
50 [122]	Therapeutic / local drug delivery	Usual medical treatments or levonorgestrel-IUS for women with heavy menstrual bleeding: long-term randomised pragmatic trial in primary care.	Br J Gen Pract	Pragmatic multicentre RCT; 5-year follow-up	571 randomized; 424 at 5 years	52-mg LNG-IUD vs usual medical treatment	2016	10.3399/bjgp16X687577	27884916	ECLIPSE trial	Scope (b): randomized trial in a mapped domain	MMAS difference 3.9 points (NS) at 5 years; surgery-free survival 80% vs 77%.
51 [56]	Hormonal IUD: dose, duration, pharmacology	A single-arm phase III study exploring the efficacy and safety of LNG-IUS 8, a low-dose levonorgestrel intrauterine contraceptive system (total content 13.5 mg), in an Asia-Pacific population.	Contraception	Phase III single-arm trial (3 years)	1,114 enrolled; 925 full analysis set	13.5-mg LNG-IUD	2017	10.1016/j.contraception.2016.11.005	27913229	LNG-IUS 8 phase III programme (Asia-Pacific)	Scope (a): report from a named device or trial program	Pearl Index 0.35; 3-year cumulative failure 0.9%; placement pain none/mild in 91.9%.
52 [78]	Hormonal IUD: dose, duration, pharmacology	Prolonged use of the etonogestrel implant and levonorgestrel intrauterine device: 2 years beyond Food and Drug Administration-approved duration.	Am J Obstet Gynecol	Prospective cohort of extended use	496 LNG-IUD users (696.9 woman-years)	52-mg LNG-IUD (Mirena) beyond 5 years	2017	10.1016/j.ajog.2017.01.036	28147241	Washington University extended-use cohort	Scope (a): report from a named device or trial program	Failure 0.25 per 100 woman-years in year 6 and 0.43 in year 7.
53 [94]	Population-level safety	IUD use among parous women and risk of uterine perforation: a secondary analysis.	Contraception	Secondary analysis of prospective cohort	61,448	LNG-IUD and copper IUDs	2017	10.1016/j.contraception.2017.03.007	28322770	EURAS-IUD	Scope (a): report from a named device or trial program	Complete perforation 4.5 vs 0.6 per 1,000 insertions in lactating vs non-lactating women.
54 [14]	Copper-device design	A randomized comparison of a novel nitinol-frame low-dose-copper intrauterine contraceptive and a copper T380S intrauterine contraceptive.	Contraception	Participant-blinded RCT (2:1)	300 (199 VC175; 101 T380S)	Cu 175 mm² nitinol frame (VeraCept) vs copper T380S	2017	10.1016/j.contraception.2017.04.002	28495299	Cu 175 mm² programme (phase 2 RCT)	Scope (b): randomized trial in a mapped domain	12-month continuation 84% vs 68%; expulsion 5.0% vs 12.0%; removal for pain/bleeding 3.5% vs 17.0%.

ID	Theme	Article	Journal	Study type	N / analysis population	Device(s)	Year	DOI	PMID	Underlying cohort / overlap	Why relevant to review	Principal quantitative / clinical finding
55 [57]	Hormonal IUD: dose, duration, pharmacology	Overcoming barriers to levonorgestrel-releasing intrauterine system placement: an evaluation of placement of LNG-IUS 8 using the modified EvoInserter® in a majority nulliparous population.	Contraception	Pooled analysis of three phase III trials	965 (83.3% nulliparous)	13.5-mg LNG-IUD with modified EvoInserter	2017	10.1016/j.contraception.2017.08.004	28844878	LNG-IUS 8 phase III programme (pooled)	Scope (a): report from a named device or trial program	Placement success 99.5%; rated easy in 91.6%; severe pain 10.0%.
56 [99]	Population-level safety	Does levonorgestrel-releasing intrauterine system increase breast cancer risk in peri-menopausal women? An HMO perspective.	Breast Cancer Res Treat	Retrospective matched cohort (HMO)	13,354 users; 27,324 controls	52-mg LNG-IUD, women aged 40–50 y	2018	10.1007/s10549-017-4491-2	28913650	Maccabi Healthcare Services cohort	Scope (c): large registry, claims or surveillance cohort	5-year invasive breast cancer 1.06% vs 0.93% (P=0.051); higher in women aged 40–45.
57 [70]	Hormonal IUD: dose, duration, pharmacology	Amenorrhea rates and predictors during 1 year of levonorgestrel 52 mg intrauterine system use.	Contraception	Analysis within single-arm phase 3 trial	1,714	52-mg LNG-IUD (Liletta)	2018	10.1016/j.contraception.2017.10.005	29038072	ACCESS IUS / NCT00995150	Scope (a): report from a named device or trial program	Amenorrhea 16.9% at 12 months in non-prior users; 1.7% discontinued for bleeding.
58 [71]	Hormonal IUD: dose, duration, pharmacology	Bleeding patterns for the Lilettalevonorgestrel 52 mg intrauterine system.	Eur J Contracept Reprod Health Care	Analysis within single-arm phase 3 trial	1,700	52-mg LNG-IUD (Liletta)	2018	10.1080/13625187.2018.1449825	29560743	ACCESS IUS / NCT00995150	Scope (a): report from a named device or trial program	Amenorrhea 19% at 1 year and 37% at 3 years; bleeding-related discontinuation 2.1% over 36 months.
59 [62]	Hormonal IUD: dose, duration, pharmacology	Twelve-month discontinuation rates of levonorgestrel intrauterine system 13.5 mg and subdermal etonogestrel implant in women aged 18-44: A retrospective claims database analysis.	Contraception	Retrospective claims cohort	3,680 LNG-IUD; 23,770 implant	13.5-mg LNG-IUD vs etonogestrel implant	2018	10.1016/j.contraception.2018.04.006	29684326	US MarketScan claims	Scope (c): large registry, claims or surveillance cohort	12-month discontinuation 24.9% vs 24.0% (HR 0.97).
60 [108]	Postpartum timing / technique	Comparative safety and efficacy of a dedicated postpartum IUD inserter versus forceps for immediate postpartum IUD insertion: a randomized trial.	Contraception	Randomized controlled trial	500 randomized; 480 completed	TCu380A immediate postpartum: dedicated inserter vs forceps	2018	10.1016/j.contraception.2018.04.019	29750926	Indian PPIUD inserter trial	Scope (b): randomized trial in a mapped domain	Complete expulsion 7.9% vs 5.4% (P=0.28); more partial expulsions with inserter; no perforations.
61 [104]	Postpartum timing / technique	Placement of Levonorgestrel Intrauterine Device at the Time of Cesarean Delivery and the Effect on Breastfeeding Duration.	Breastfeed Med	Secondary analysis of RCT	63 LNG-IUD users	52-mg LNG-IUD intracesarean vs interval	2018	10.1089/bfm.2018.0060	30376369	UNC intracesarean IUD trial	Scope (b): randomized trial in a mapped domain	No difference in breastfeeding at 6, 12 or 24 weeks.
62 [66]	Population-level safety	Levonorgestrel intrauterine contraceptive systems (13.5 mg and 52 mg) and risk of ectopic pregnancy.	Acta Obstet Gynecol Scand	Hospital-based historical cohort	48 ectopic cases; 68,734 exposed woman-years	13.5-mg vs 52-mg LNG-IUD	2019	10.1111/aogs.13564	30737766	Stockholm ectopic-pregnancy cohort (2013–2017)	Scope (c): large registry, claims or surveillance cohort	Ectopic Pearl Index 0.19 (13.5 mg) vs 0.006 (52 mg).
63 [101]	Population-level safety	Safety of levonorgestrel 52 mg intrauterine system compared to copper intrauterine device: a population-based cohort study.	Contraception	Propensity-matched claims cohort	9,318 LNG-IUD; 10,185 copper	52-mg LNG-IUD vs copper IUD	2019	10.1016/j.contraception.2019.02.011	30871933	French SNDS	Scope (c): large registry, claims or surveillance cohort	Anxiolytic initiation HR 1.08 and removal HR 1.05; no difference in ectopic pregnancy, PID or perforation.
64 [49]	Hormonal IUD: dose, duration, pharmacology	Bleeding patterns with the 19.5 mg LNG-IUS, with special focus on the first year of use: implications for counselling.	Eur J Contracept Reprod Health Care	Pooled analysis of phase II and III trials	1,697	19.5-mg LNG-IUD	2019	10.1080/13625187.2019.1630817	31223042	LNG-IUS 12 phase II/III programme	Scope (a): report from a named device or trial program	≤4 bleeding/spotting days per 30 days in 6.2% at month 1 and 54.1% at month 12.
65 [109]	Postpartum timing / technique	Importance of the delivery-to-insertion interval in immediate postpartum intrauterine device insertion: A secondary analysis.	Int J Gynaecol Obstet	Secondary analysis of two related studies	560	TCu380A within 48 h of vaginal delivery	2020	10.1002/ijgo.13115	32037531	Indian PPIUD inserter trial	Scope (a): report from a named device or trial program	Complete expulsion 3.2% postplacental vs 7.5% immediate (>10 min; P=0.176).
66 [92]	Copper-device design	Efficacy, Safety, and Tolerability of a New Low-Dose Copper and Nitinol Intrauterine Device: Phase 2 Data to 36 Months.	Obstet Gynecol	Phase 2 single-arm trial with 2-year extension	286	Cu 175 mm² nitinol-frame IUD	2020	10.1097/AOG.0000000000003756	32168217	Cu 175 mm² programme (phase 2)	Scope (a): report from a named device or trial program	Pearl Index 0.46 over 36 months; placement 99.0%; expulsion 1.8%.
67 [51]	Hormonal IUD: dose, duration, pharmacology	Insertion experience of women and health care professionals in the KyleenaSatisfaction Study.	Eur J Contracept Reprod Health Care	Prospective observational study (baseline analysis)	1,110	19.5-mg LNG-IUD (Kyleena)	2020	10.1080/13625187.2020.1736547	32223466	KYSS / NCT03182140	Scope (a): report from a named device or trial program	Insertion easy in 92.2% parous and 89.9%

ID	Theme	Article	Journal	Study type	N / analysis population	Device(s)	Year	DOI	PMID	Underlying cohort / overlap	Why relevant to review	Principal quantitative / clinical finding
												nulliparous; no/mild pain 88.6% vs 67.4%.
68 [47]	Hormonal IUD: dose, duration, pharmacology	Comparative pharmacokinetic analysis of levonorgestrel-releasing intrauterine systems and levonorgestrel-containing contraceptives with oral or subdermal administration route.	Eur J Contracept Reprod Health Care	Integrated population pharmacokinetic analysis	> 3,400 from 10 studies	13.5-, 19.5- and 52-mg LNG-IUDs vs implant and oral LNG	2020	10.1080/13625187.2020.1815008	33006493	Bayer pooled PK dataset	Scope (a): report from a named device or trial program	Systemic LNG lowest with LNG-IUDs; COC peak 8.6-fold higher than 52-mg LNG-IUD.
69 [74]	Hormonal IUD: dose, duration, pharmacology	Conception rates in women desiring pregnancy after levonorgestrel 52 mg intrauterine system (Liletta®) discontinuation.	Contraception	Analysis within single-arm phase 3 trial	165 attempting conception	52-mg LNG-IUD (Liletta)	2021	10.1016/j.contraception.2020.09.005	33038303	ACCESS IUS / NCT00995150	Scope (a): report from a named device or trial program	86.1% conceived within 12 months of removal; no difference by gravidity or parity.
70 [50]	Hormonal IUD: dose, duration, pharmacology	Predicting menstrual bleeding patterns with levonorgestrel-releasing intrauterine systems.	Eur J Contracept Reprod Health Care	Bleeding-prediction modelling study	1,351 + 216 + 1,300	13.5-, 19.5- and 52-mg LNG-IUDs	2021	10.1080/13625187.2020.1843015	33269954	LNG-IUS phase III programme (pooled)	Scope (a): report from a named device or trial program	Fixed-cluster model classified 69–72% of women correctly.
71 [126]	Therapeutic / local drug delivery	Treatment of women with heavy menstrual bleeding: Results of a prospective cohort study alongside a randomised controlled trial.	Eur J Obstet Gynecol Reprod Biol	Prospective preference cohort alongside RCT	276 (87 LNG-IUD; 189 ablation)	52-mg LNG-IUD vs endometrial ablation	2021	10.1016/j.ejogrb.2020.11.071	33309849	MIRA programme (preference cohort)	Scope (a): report from a named device or trial program	24-month reintervention 35% vs 14% (aRR 2.42).
72 [106]	Postpartum timing / technique	One-year follow up on post-placental IUD insertion: A randomized clinical trial.	Acta Obstet Gynecol Scand	Randomized controlled trial; 1-year follow-up	140 (70/70)	TCu380A vs 52-mg LNG-IUD, postplacental	2021	10.1111/aogs.14081	33421091	Campinas postplacental trial	Scope (b): randomized trial in a mapped domain	1-year expulsion 39.4% vs 22.2%; continuation 54.4% vs 73.1%.
73 [103]	Postpartum timing / technique	Comparison of levonorgestrel level and creamatocrit in milk following immediate versus delayed postpartum placement of the levonorgestrel IUD.	BMC Womens Health	Breast-milk substudy of RCT	32 (22 with paired samples)	52-mg LNG-IUD immediate vs delayed postpartum	2021	10.1186/s12905-021-01179-7	33478494	Utah postpartum breastfeeding trial / NCT01990703	Scope (a): report from a named device or trial program	Milk LNG low in both groups and similar after 4 weeks; creamatocrit unchanged.
74 [72]	Hormonal IUD: dose, duration, pharmacology	Relationship of parity and prior cesarean delivery to levonorgestrel 52 mg intrauterine system expulsion over 6 years.	Contraception	Analysis within single-arm phase 3 trial	1,710	52-mg LNG-IUD (Liletta)	2021	10.1016/j.contraception.2021.02.013	33651995	ACCESS IUS / NCT00995150	Scope (a): report from a named device or trial program	Expulsion 3.8% over 72 months; 7.2% in parous women with vaginal deliveries only; obesity aOR 2.2.
75 [116]	Emergency contraception	Risk of Pregnancy With Levonorgestrel-Releasing Intrauterine System Placement 6-14 Days After Unprotected Sexual Intercourse.	Obstet Gynecol	Prospective observational cohort (research letter)	187 (pooled from three observational studies and one RCT)	52-mg LNG-IUD placed 6–14 days after intercourse	2021	10.1097/AOG.0000000000004118	33706343	Utah EC programme (observational arm)	Scope (a): report from a named device or trial program	One pregnancy among 187 women given a 52-mg LNG-IUD 6–14 days after unprotected intercourse (0.5%; 95% CI 0.01–2.9).
76 [85]	Copper-device design	Copper IUD continuation, unwanted effects and cost consequences at 1 year in users aged under 30 - a secondary analysis of the EURAS-IUD study.	Eur J Contracept Reprod Health Care	Secondary analysis of prospective cohort	5,762 users aged < 30 y	Copper IUDs by surface area, frame, width	2021	10.1080/13625187.2021.1879783	33715567	EURAS-IUD	Scope (a): report from a named device or trial program	Higher continuation and fewer unwanted effects with < 300 mm² copper, horseshoe frames and flexible arms.
77 [129]	Therapeutic / local drug delivery	Long-term oncological and reproductive outcomes following treatment with the levonorgestrel intra-uterine device for endometrial hyperplasia and adenocarcinoma: results from the feMMe phase 2 randomized clinical trial.	Int J Gynecol Cancer	Phase II RCT	165 enrolled; 154 at 6 months	52-mg LNG-IUD ± metformin or weight loss	2026	10.1016/j.ijgc.2026.104725	42167104	feMME trial / NCT01686126	Scope (b): randomized trial in a mapped domain	Pathological complete response at 6 months 82% for AEH and 43% for endometrial adenocarcinoma.
78 [114]	Emergency contraception	Rates of pregnancy among levonorgestrel and copper intrauterine emergency contraception initiators: Implications for backup contraception recommendations.	Contraception	Secondary analysis of RCT	518	52-mg LNG-IUD vs copper T380A for EC	2021	10.1016/j.contraception.2021.06.011	34166648	Utah EC trial / NCT02175030	Scope (a): report from a named device or trial program	No pregnancies among those with intercourse ≤7 days after placement (0/138 LNG; 0/148 copper).

ID	Theme	Article	Journal	Study type	N / analysis population	Device(s)	Year	DOI	PMID	Underlying cohort / overlap	Why relevant to review	Principal quantitative / clinical finding
79 [121]	Therapeutic / local drug delivery	Comparison of two levonorgestrel-releasing intrauterine systems for the treatment of heavy menstrual bleeding: a randomised, controlled, phase 3 trial.	Eur J Contracept Reprod Health Care	Randomized non-inferiority phase 3 trial	312	52-mg LNG-IUD (Levosert/Donasert) vs Mirena for HMB	2021	10.1080/13625187.2021.1942447	34180315	Russian Levosert/Donasert registration trial	Scope (b): randomized trial in a mapped domain	6-month MBL change −130 vs −127 mL; non-inferior.
80 [115]	Emergency contraception	Pregnancy Risk by Frequency and Timing of Unprotected Intercourse Before Intrauterine Device Placement for Emergency Contraception.	Obstet Gynecol	Secondary analysis of RCT	655	52-mg LNG-IUD vs copper T380A for EC	2021	10.1097/AOG.0000000000004433	34259467	Utah EC trial / NCT02175030	Scope (a): report from a named device or trial program	No pregnancies with multiple or 6–14-day-prior intercourse episodes.
81 [53]	Hormonal IUD: dose, duration, pharmacology	Satisfaction and continuation with LNG-IUS 12: findings from the real-world kyleenasatisfaction study.	Eur J Contracept Reprod Health Care	Prospective observational study	1,129	19.5-mg LNG-IUD (Kyleena)	2021	10.1080/13625187.2021.1975268	34528857	KYSS / NCT03182140	Scope (a): report from a named device or trial program	12-month satisfaction 86.1%; continuation 81.4%.
82 [87]	Copper-device design	Assessment of a Novel Fixation Method of a Frameless Intrauterine Contraceptive Device Inserted during Cesarean Delivery as a Means of Preventing Displacements and Expulsions: A Prospective Observational Study.	Life (Basel)	Prospective observational case series	21	Gyn-CS anchored frameless copper IUD with serosal suture fixation, intracesarean	2022	10.3390/life12010083	35054475	Gyn-CS programme (Gazi University series)	Scope (a): report from a named device or trial program	No displacement or expulsion among 21 women followed to ≥3 months.
83 [105]	Postpartum timing / technique	Long-acting reversible contraception (LARC) after pregnancy and childbirth	PhD thesis, Karolinska Institutet	Randomized non-inferiority trial (stopped early)	101 (52/49)	52-mg LNG-IUD ≤48 h vs 6–8 weeks after vaginal birth	2023	ISBN 978-91-8016-885-4	—	Swedish early postpartum trial	Scope (b): randomized trial in a mapped domain	Stopped for expulsion: 44.2% early vs 0% standard; method use at 1 year 71% vs 83.7%.
84 [96]	Population-level safety	Association of the Timing of Postpartum Intrauterine Device Insertion and Breastfeeding With Risks of Intrauterine Device Expulsion.	JAMA Netw Open	Retrospective cohort	326,658 (94,817 subcohort)	LNG-IUDs and copper IUDs	2022	10.1001/jamanetworkopen.2021.48474	35226086	APEX-IUD	Scope (c): large registry, claims or surveillance cohort	5-year expulsion 10.73% at 0–3 days postpartum; aHR 5.34; breastfeeding aHR 0.71.
85 [97]	Population-level safety	Association between menorrhagia and risk of intrauterine device-related uterine perforation and device expulsion: results from the Association of Uterine Perforation and Expulsion of Intrauterine Device study.	Am J Obstet Gynecol	Retrospective cohort	228,834 non-postpartum	LNG-IUDs and copper IUDs	2022	10.1016/j.ajog.2022.03.025	35292234	APEX-IUD	Scope (c): large registry, claims or surveillance cohort	Menorrhagia diagnosis: expulsion aHR 2.84; perforation aHR 1.53.
86 [65]	Population-level safety	Method of Hormonal Contraception and Protective Effects Against Ectopic Pregnancy.	Obstet Gynecol	Nationwide register cohort	1,663,242	Hormonal methods incl. 13.5- and 52-mg LNG-IUD	2022	10.1097/AOG.0000000000004726	35576335	Swedish Prescribed Drug Register	Scope (c): large registry, claims or surveillance cohort	Ectopic pregnancy 2.76 vs 0.30 per 1,000 woman-years for 13.5- vs 52-mg LNG-IUD.
87 [67]	Population-level safety	The 13.5-mg, 19.5-mg, and 52-mg Levonorgestrel-Releasing Intrauterine Systems and Risk of Ectopic Pregnancy.	Obstet Gynecol	Retrospective cohort	2,252 ectopic cases (94 with LNG-IUD)	13.5-, 19.5- and 52-mg LNG-IUDs	2022	10.1097/AOG.0000000000004846	35852273	Stockholm ectopic-pregnancy cohort (2014–2019)	Scope (c): large registry, claims or surveillance cohort	Ectopic Pearl Index 0.136, 0.037 and 0.009 for 13.5, 19.5 and 52 mg.
88 [118]	Emergency contraception	Switching and discontinuation of participant-masked randomization to a copper or levonorgestrel intrauterine device when presenting for emergency contraception.	Contraception	Secondary analysis of RCT	655	52-mg LNG-IUD vs copper T380A for EC	2023	10.1016/j.contraception.2022.09.131	36240903	Utah EC trial / NCT02175030	Scope (b): randomized trial in a mapped domain	6-month switching 2.1% vs 5.5%; discontinuation 10.4% vs 10.7%.
89 [98]	Population-level safety	Demographic, Reproductive, and Medical Risk Factors for Intrauterine Device Expulsion.	Obstet Gynecol	Retrospective cohort	228,834 non-postpartum	LNG-IUDs and copper IUDs	2022	10.1097/AOG.0000000000005000	36357958	APEX-IUD	Scope (c): large registry, claims or surveillance cohort	Heavy menstrual bleeding strongest expulsion risk factor; higher risk with obesity, age ≤24 y, parity ≥4.
90 [133]	Therapeutic / local drug delivery	The effect of a combined indomethacin and levonorgestrel-releasing intrauterine system on short-term postplacement	Am J Obstet Gynecol	Phase II dose-finding RCT	174 randomized; 160 per protocol	Indomethacin/13.5-mg LNG-IUD vs LNG-IUD alone	2023	10.1016/j.ajog.2022.10.025	36424684	Indomethacin–LNG-IUD programme	Scope (b): randomized trial in a mapped domain	Low-dose indomethacin reduced bleeding/spotting days in first 90 days by 32%.

ID	Theme	Article	Journal	Study type	N / analysis population	Device(s)	Year	DOI	PMID	Underlying cohort / overlap	Why relevant to review	Principal quantitative / clinical finding
		bleeding profile: a randomized proof-of-concept trial.										
91 [30]	Therapeutic / local drug delivery	Vaginal micronized progesterone versus the levonorgestrel-releasing intrauterine system for treatment of non-atypical endometrial hyperplasia: A randomized controlled trial.	Int J Gynaecol Obstet	Randomized controlled trial	144 randomized; 138 analysed	52-mg LNG-IUD vs vaginal micronized progesterone	2023	10.1002/ijgo.14632	36527262	Independent EH trial	Scope (b): randomized trial in a mapped domain	3-month regression of non-atypical hyperplasia 95.8% vs 90.8% (P=0.194).
92 [69]	Population-level safety	Association Between Doses of Levonorgestrel Intrauterine Systems and Subsequent Use of Psychotropic Drugs in France.	JAMA	Nationwide claims cohort (research letter)	45,736 per group (matched)	13.5-, 19.5- and 52-mg LNG-IUDs	2023	10.1001/jama.2022.21471	36548007	French SNDS	Scope (c): large registry, claims or surveillance cohort	Antidepressant use slightly higher with the 52-mg than with the 19.5-mg LNG-IUD (aOR 1.13, 95% CI 1.05–1.21, as cited by ID 107); anxiolytic and hypnotic use not significantly different.
93 [130]	Therapeutic / local drug delivery	Comparison of the effect of oral megestrol acetate with or without levonorgestrel-intrauterine system on fertility-preserving treatment in patients with early-stage endometrial cancer: a prospective, open-label, randomized controlled phase II trial (ClinicalTrials.gov NCT03241914).	J Gynecol Oncol	Phase II RCT	63 randomized; 54 analysed	Megestrol acetate ± 52-mg LNG-IUD for early endometrial cancer	2023	10.3802/jgo.2023.34.e32	36562136	Fudan EEC trial / NCT03241914	Scope (b): randomized trial in a mapped domain	16-week complete response 25.0% vs 19.2% (NS); more vaginal bleeding with LNG-IUD.
94 [134]	Therapeutic / local drug delivery	Development of an Intrauterine Device Releasing Both Indomethacin and Levonorgestrel During the First Months of Use: Pharmacokinetic Characterization in Healthy Women.	Clin Pharmacokinet	Pharmacokinetic substudy of phase II RCT	174 (62 dense sampling)	Indomethacin/13.5-mg LNG-IUD	2023	10.1007/s40262-022-01201-7	36648744	Indomethacin–LNG-IUD programme	Scope (a): report from a named device or trial program	Two drugs released at independent rates; indomethacin did not alter LNG PK.
95 [91]	Copper-device design	A 5-year comparative study of efficacy and acceptability of three different sizes of TCu380Ag and TCu380A intrauterine devices.	Int J Gynaecol Obstet	Randomized controlled trial; 5-year follow-up	600	TCu380Ag (three sizes) vs TCu380A	2023	10.1002/ijgo.14651	36652393	Indian TCu380Ag trial	Scope (b): randomized trial in a mapped domain	5-year continuation 45% vs 35%; expulsion 4.4% vs 8.7%; no pregnancies.
96 [63]	Adolescents	Pain and ease of insertion of three different intrauterine devices in Brazilian adolescents: A participant-blinded randomized trial.	Contraception	Participant-blinded RCT	318 adolescents	TCu380A vs 52-mg vs 19.5-mg LNG-IUD	2023	10.1016/j.contraception.2023.109997	36841463	Brazilian adolescent IUD trial	Scope (b): randomized trial in a mapped domain	Median VAS pain 8.0 with 52-mg LNG-IUD vs 7.0 with others; placement easier with copper and 19.5 mg.
97 [88]	Copper-device design	Effectiveness, safety, and acceptability of postplacental insertion of GyneFix postpartum intrauterine device among women undergoing cesarean section: A multicenter prospective cohort study in China.	Contraception	Prospective multicentre cohort	470 (400 at 12 months)	GyneFix postpartum frameless copper IUD at cesarean	2023	10.1016/j.contraception.2023.109999	36849032	Chinese GyneFix PPIUD cohort	Scope (a): report from a named device or trial program	12-month expulsion 7.6%; continuation 86.6%; Pearl Index 2.3 overall.
98 [131]	Therapeutic / local drug delivery	Comparing levonorgestrel intrauterine system with hysteroscopic niche resection in women with postmenstrual spotting related to a niche in the uterine cesarean scar: a randomized, open-label, controlled trial.	Am J Obstet Gynecol	Randomized open-label trial	208 (104/104)	52-mg LNG-IUD vs hysteroscopic niche resection	2023	10.1016/j.ajog.2023.03.020	36935068	Shanghai niche trial	Scope (b): randomized trial in a mapped domain	≥50% spotting reduction at 6 months 78.4% vs 73.1% (NS); superior from 9 months.
99 [112]	Copper-device design	Does change in the shape of intrauterine contraceptive device Cu 375 and Cu T 380A affect the expulsion after post-partum insertion - a prospective randomized control trial.	Eur J Contracept Reprod Health Care	Randomized controlled trial	396	Cu 375 vs CuT380A postpartum	2023	10.1080/13625187.2023.2188104	36995695	Indian PPIUCD shape trial	Scope (b): randomized trial in a mapped domain	Complete expulsion at 6 weeks 5.3% vs 6.7% (NS); lower after postplacental insertion.

ID	Theme	Article	Journal	Study type	N / analysis population	Device(s)	Year	DOI	PMID	Underlying cohort / overlap	Why relevant to review	Principal quantitative / clinical finding
100 [31]	Therapeutic / local drug delivery	Comparison of the effect of levonorgestrel-intrauterine system with or without oral megestrol acetate on fertility-preserving treatment in patients with atypical endometrial hyperplasia: A prospective, open-label, randomized controlled phase II study.	Gynecol Oncol	Phase II RCT (three arms)	180	52-mg LNG-IUD ± megestrol vs megestrol for AEH	2023	10.1016/j.ygyno.2023.05.001	37182434	Fudan AEH trial / NCT03241888	Scope (b): randomized trial in a mapped domain	16-week complete response 35.0% (LNG-IUD), 29.4% (combination), 19.2% (megestrol); NS.
101 [111]	Postpartum timing / technique	Conventional manual technique of post placental IUD insertion versus intra-cesarean post placental introducer withdrawal IUD insertion technique: a new standardized technique for IUD insertion during cesarean section: a randomized controlled trial.	BMC Pregnancy Childbirth	Randomized controlled trial	840 (420/420)	TCu380A intracesarean: introducer-withdrawal vs manual	2023	10.1186/s12884-023-05777-1	37365489	Cairo intracesarean technique trial	Scope (b): randomized trial in a mapped domain	Displacement at 6 months 11.9% vs 20.1%; non-visible strings 3.6% vs 9.6%; continuation 88.1% vs 79.9% with the introducer-withdrawal technique.
102 [95]	Population-level safety	Risks of Uterine Perforation and Expulsion Associated With Intrauterine Devices.	Obstet Gynecol	Summary of retrospective cohort	326,658	LNG-IUDs and copper IUDs	2023	10.1097/AOG.000000000000005299	37535968	APEX-IUD	Scope (c): large registry, claims or surveillance cohort	5-year perforation 0.61%; highest 4 days–6 weeks postpartum (aHR 6.71).
103 [123]	Therapeutic / local drug delivery	Rates of medical or surgical treatment for women with heavy menstrual bleeding: the ECLIPSE trial 10-year observational follow-up study.	Health Technol Assess	10-year observational follow-up of RCT	206	52-mg LNG-IUD vs usual medical treatment	2023	10.3310/JHSW0174	37924269	ECLIPSE trial	Scope (a): report from a named device or trial program	Surgery in 29% over 10 years; 28% using LNG-IUD at 10 years; no QoL difference.
104 [61]	Hormonal IUD: dose, duration, pharmacology	Health-related quality of life and satisfaction associated with 3-year use of a levonorgestrel 13.5 mg intrauterine device vs Nova T copper 380 mmintrauterine device: Results of a phase 4 randomized controlled trial.	Contraception	Secondary outcomes of phase 4 RCT	106 (55/51)	13.5-mg LNG-IUD vs Nova T Cu380	2024	10.1016/j.contraception.2024.110367	38232939	Perelló-Capó phase 4 trial	Scope (b): randomized trial in a mapped domain	3-year SEC-QoL 69.2 vs 52.5 (P=0.002); very satisfied 82% vs 32%.
105 [54]	Hormonal IUD: dose, duration, pharmacology	The use of LNG-IUS-19.5 mg in daily gynecological routine practice in Germany: data from the Kyleena™ Satisfaction Study (KYSS).	Arch Gynecol Obstet	Prospective observational study (national subset)	508	19.5-mg LNG-IUD (Kyleena)	2024	10.1007/s00404-024-07421-5	38421421	KYSS / NCT03182140	Scope (a): report from a named device or trial program	12-month satisfaction 87.6%; continuation 84.1%.
106 [90]	Copper-device design	Menstrual cup use and intrauterine device expulsion in a copper intrauterine device randomized trial.	Contraception	Secondary analysis of RCT	1,046	NTCu380-Mini vs TCu380A	2024	10.1016/j.contraception.2024.110415	38431257	NTCu380-Mini trial	Scope (a): report from a named device or trial program	36-month expulsion 23.2% vs 9.4% in menstrual-cup users vs non-users; aOR 3.13 with NTCu380-Mini.
107 [68]	Population-level safety	Depression risk in users of different doses of levonorgestrel intrauterine systems: a nationwide prospective cohort study.	Lancet Reg Health Eur	Nationwide prospective cohort	46,565	13.5-, 19.5- and 52-mg LNG-IUDs	2024	10.1016/j.lanepe.2023.100813	38476740	Danish national registers	Scope (c): large registry, claims or surveillance cohort	Depression rate ratio 0.77 (19.5 mg) and 0.85 (13.5 mg) vs 52 mg.
108 [102]	Population-level safety	Use of progestogens and the risk of intracranial meningioma: national case-control study.	BMJ	National case–control study	18,061 cases; 90,305 controls	Progestogens incl. 13.5- and 52-mg LNG-IUDs	2024	10.1136/bmj-2023-078078	38537944	French SNDS	Scope (c): large registry, claims or surveillance cohort	No excess meningioma risk with the 52-mg (OR 0.94, 95% CI 0.86–1.04) or 13.5-mg LNG-IUD (OR 1.39, 0.70–2.77).
109 [75]	Hormonal IUD: dose, duration, pharmacology	Hormone-related side effects in new users of a levonorgestrel 52-mg intrauterine device.	Am J Obstet Gynecol	Analysis within single-arm phase 3 trial	1,499	52-mg LNG-IUD (Liletta)	2024	10.1016/j.ajog.2024.06.049	38969201	ACCESS IUS / NCT00995150	Scope (a): report from a named device or trial program	Hormone-related side effects rare; acne more frequent after prior combined hormonal use (aOR 1.40); discontinuation 5.5%.
110 [128]	Therapeutic / local drug delivery	A multi-centre randomised controlled trial comparing megestrol acetate to levonorgestrel-intrauterine system	J Assist Reprod Genet	Multicentre phase II RCT	40 randomized (21/19); 36 completed	52-mg LNG-IUD vs megestrol acetate for AEH	2024	10.1007/s10815-024-03172-z	39215793	Singapore AEH trial	Scope (b): randomized trial in a mapped domain	9-month regression 88.9% overall; 3-month regression 61.1% (LNG-IUD) vs 38.9% (megestrol), not significant;

ID	Theme	Article	Journal	Study type	N / analysis population	Device(s)	Year	DOI	PMID	Underlying cohort / overlap	Why relevant to review	Principal quantitative / clinical finding
		in fertility sparing treatment of atypical endometrial hyperplasia.										underpowered (40 of 60 planned).
111 [117]	Emergency contraception	Estimating emergency contraception efficacy with levonorgestrel and copper intrauterine devices.	Contraception	Secondary analysis of RCT	630	52-mg LNG-IUD vs copper T380A for EC	2025	10.1016/j.contraception.2025.110946	40368317	Utah EC trial / NCT02175030	Scope (a): report from a named device or trial program	LNG-IUD prevented 93.2–95.7% and copper IUD 100% of expected pregnancies.
112 [110]	Postpartum timing / technique	Manual versus forceps postplacental IUD insertion after vaginal delivery: A randomized clinical trial.	Int J Gynaecol Obstet	Randomized controlled trial	210 (105/105)	Copper IUD postplacental: manual vs ring forceps	2026	10.1002/ijgo.70355	40621995	Campinas insertion-technique trial	Scope (b): randomized trial in a mapped domain	Complete expulsion 16.7% manual vs 25% forceps (P=0.168); less discomfort with forceps.
113 [132]	Therapeutic / local drug delivery	Levonorgestrel intrauterine system vs hysteroscopic niche resection for postmenstrual spotting: a 3-year randomized trial follow-up.	Am J Obstet Gynecol	3-year follow-up of RCT	208	52-mg LNG-IUD vs hysteroscopic niche resection	2026	10.1016/j.ajog.2025.09.011	40945810	Shanghai niche trial	Scope (b): randomized trial in a mapped domain	≥50% spotting reduction at 36 months 98.9% vs 51.7% (RR 1.91).
114 [100]	Population-level safety	Hormonal Contraceptive Formulations and Breast Cancer Risk in Adolescents and Premenopausal Women.	JAMA Oncol	Nationwide register cohort	2,095,130	Hormonal contraceptives incl. 52-mg LNG-IUD	2025	10.1001/jamaoncol.2025.4480	41165687	Swedish national registers	Scope (c): large registry, claims or surveillance cohort	52-mg LNG-IUD HR 1.13 (95% CI 1.09–1.18; about 8 additional cases per 100,000 person-years); any hormonal contraception HR 1.24.

Table S3. Electronic search strategies, search dates and records retrieved per source

Part A gives the targeted search run during revision: nine program- and domain-specific blocks, each shown with its PubMed/MEDLINE syntax and the corresponding Scopus and Web of Science Core Collection syntax, followed by the ClinicalTrials.gov search. Part B reproduces the original search (last run on 5 September 2026), which was superseded by the reviewed part.

Part A. Targeted search (PubMed/MEDLINE run on 12 October 2026)

Block 1. LNG-IUD brand and product names

PubMed/MEDLINE: Liletta[tiab] OR "ACCESS IUS"[tiab] OR Mirena[tiab] OR Kyleena[tiab] OR Jaydess[tiab] OR Skyla[tiab] OR Levosert[tiab] OR Benilexa[tiab] OR "LNG-IUS 12"[tiab] OR "LNG-IUS 8"[tiab] OR "Mirena Extension"[tiab]

Run 12 October 2026; publication date 2016/01/01 to 2026/09/05; records retrieved: 212.

Scopus: TITLE-ABS-KEY(Liletta OR "ACCESS IUS" OR Mirena OR Kyleena OR Jaydess OR Skyla OR Levosert OR Benilexa OR "LNG-IUS 12" OR "LNG-IUS 8" OR "Mirena Extension") AND PUBYEAR > 2015 AND PUBYEAR < 2027 AND (DOCTYPE(ar) OR DOCTYPE(le))

Run 12 October 2026; records retrieved: 212; no unique records beyond PubMed/MEDLINE.

Web of Science Core Collection: TS=(Liletta OR "ACCESS IUS" OR Mirena OR Kyleena OR Jaydess OR Skyla OR Levosert OR Benilexa OR "LNG-IUS 12" OR "LNG-IUS 8" OR "Mirena Extension") AND PY=(2016-2026) AND DT=(Article OR Letter)

Run 12 October 2026; records retrieved: 212; no unique records beyond PubMed/MEDLINE.

Block 2. LNG-IUD hormone doses

PubMed/MEDLINE: ("19.5 mg"[tiab] OR "13.5 mg"[tiab] OR "52 mg"[tiab] OR "52-mg"[tiab]) AND (intrauterine[tiab] OR IUD[tiab] OR IUS[tiab])

Run 12 October 2026; publication date 2016/01/01 to 2026/09/05; records retrieved: 240.

Scopus: TITLE-ABS-KEY(("19.5 mg" OR "13.5 mg" OR "52 mg" OR "52-mg") AND (intrauterine OR IUD OR IUS)) AND PUBYEAR > 2015 AND PUBYEAR < 2027 AND (DOCTYPE(ar) OR DOCTYPE(le))

Run 12 October 2026; records retrieved: 240; no unique records beyond PubMed/MEDLINE.

Web of Science Core Collection: TS=(("19.5 mg" OR "13.5 mg" OR "52 mg" OR "52-mg") AND (intrauterine OR IUD OR IUS)) AND PY=(2016-2026) AND DT=(Article OR Letter)

Run 12 October 2026; records retrieved: 240; no unique records beyond PubMed/MEDLINE.

Block 3. Named and novel copper devices

PubMed/MEDLINE: ("NTCu380-Mini"[tiab] OR "Mona Lisa"[tiab] OR VeraCept[tiab] OR Miudella[tiab] OR "Cu 175"[tiab] OR "low-dose copper"[tiab] OR Ballerine[tiab] OR "intrauterine ball"[tiab] OR SCu380A[tiab] OR "Gyn-CS"[tiab] OR GyneFix[tiab] OR frameless[tiab]) AND (intrauterine[tiab] OR IUD[tiab] OR contraception[tiab] OR contraceptive[tiab])

Run 12 October 2026; publication date 2016/01/01 to 2026/09/05; records retrieved: 42.

Scopus: TITLE-ABS-KEY(("NTCu380-Mini" OR "Mona Lisa" OR VeraCept OR Miudella OR "Cu 175" OR "low-dose copper" OR Ballerine OR "intrauterine ball" OR SCu380A OR "Gyn-CS" OR GyneFix OR frameless) AND (intrauterine OR IUD OR contracepti)) AND PUBYEAR > 2015 AND PUBYEAR < 2027 AND (DOCTYPE(ar) OR DOCTYPE(le))

Run 12 October 2026; records retrieved: 42; no unique records beyond PubMed/MEDLINE.

Web of Science Core Collection: TS=(("NTCu380-Mini" OR "Mona Lisa" OR VeraCept OR Miudella OR "Cu 175" OR "low-dose copper" OR Ballerine OR "intrauterine ball" OR SCu380A OR "Gyn-CS" OR GyneFix OR frameless) AND (intrauterine OR IUD OR contracepti)) AND PY=(2016-2026) AND DT=(Article OR Letter)

Run 12 October 2026; records retrieved: 42; no unique records beyond PubMed/MEDLINE.

Block 4. Copper alloys, nitinol and device shape

PubMed/MEDLINE: (silver OR gold OR nitinol OR "shape memory" OR alloy OR "Tatum-T" OR "device shape")[tiab] AND (IUD OR "intrauterine device" OR "intrauterine devices" OR "copper intrauterine")[tiab]

Run 12 October 2026; publication date 2016/01/01 to 2026/09/05; records retrieved: 43.

Scopus: TITLE-ABS-KEY((silver OR gold OR nitinol OR "shape memory" OR alloy OR "Tatum-T" OR "device shape") AND (IUD OR "intrauterine device" OR "copper intrauterine")) AND PUBYEAR > 2015 AND PUBYEAR < 2027 AND (DOCTYPE(ar) OR DOCTYPE(le))

Run 12 October 2026; records retrieved: 43; no unique records beyond PubMed/MEDLINE.

Web of Science Core Collection: TS=((silver OR gold OR nitinol OR "shape memory" OR alloy OR "Tatum-T" OR "device shape") AND (IUD OR "intrauterine device" OR "copper intrauterine")) AND PY=(2016-2026) AND DT=(Article OR Letter)

Run 12 October 2026; records retrieved: 43; no unique records beyond PubMed/MEDLINE.

Block 5. Surveillance programs

PubMed/MEDLINE: EURAS[tiab] OR "European Active Surveillance"[tiab] OR "APEX-IUD"[tiab] OR "APEX IUD"[tiab] OR "Association of Perforation and Expulsion"[tiab] OR LCS12[tiab]

Run 12 October 2026; publication date 2016/01/01 to 2026/09/05; records retrieved: 19.

Scopus: TITLE-ABS-KEY(EURAS OR "European Active Surveillance" OR "APEX-IUD" OR "APEX IUD" OR "Association of Perforation and Expulsion" OR LCS12) AND PUBYEAR > 2015 AND PUBYEAR < 2027 AND (DOCTYPE(ar) OR DOCTYPE(le))

Run 12 October 2026; records retrieved: 19; no unique records beyond PubMed/MEDLINE.

Web of Science Core Collection: TS=(EURAS OR "European Active Surveillance" OR "APEX-IUD" OR "APEX IUD" OR "Association of Perforation and Expulsion" OR LCS12) AND PY=(2016-2026) AND DT=(Article OR Letter)

Run 12 October 2026; records retrieved: 19; no unique records beyond PubMed/MEDLINE.

Block 6. Extended use and pharmacokinetics

PubMed/MEDLINE: ("extended use" OR "extended duration" OR "beyond 5 years" OR "8 years" OR "residual levonorgestrel" OR "release rate" OR "release rates" OR pharmacokinetic)[tiab] AND ("levonorgestrel intrauterine" OR "levonorgestrel-releasing intrauterine" OR "LNG-IUS" OR "LNG-IUD")[tiab]

Run 12 October 2026; publication date 2016/01/01 to 2026/09/05; records retrieved: 45.

Scopus: TITLE-ABS-KEY(("extended use" OR "extended duration" OR "beyond 5 years" OR "8 years" OR "residual levonorgestrel" OR "release rate" OR pharmacokinetic) AND ("levonorgestrel intrauterine" OR "levonorgestrel-releasing intrauterine" OR "LNG-IUS" OR "LNG-IUD")) AND PUBYEAR > 2015 AND PUBYEAR < 2027 AND (DOCTYPE(ar) OR DOCTYPE(le))

Run 12 October 2026; records retrieved: 45; no unique records beyond PubMed/MEDLINE.

Web of Science Core Collection: TS=(("extended use" OR "extended duration" OR "beyond 5 years" OR "8 years" OR "residual levonorgestrel" OR "release rate" OR pharmacokinetic) AND ("levonorgestrel intrauterine" OR "levonorgestrel-releasing intrauterine" OR "LNG-IUS" OR "LNG-IUD")) AND PY=(2016-2026) AND DT=(Article OR Letter)

Run 12 October 2026; records retrieved: 45; no unique records beyond PubMed/MEDLINE.

Block 7. Randomized trials: emergency contraception and therapeutic indications

PubMed/MEDLINE: ("emergency contraception"[tiab] OR "heavy menstrual bleeding"[tiab] OR menorrhagia[tiab] OR "endometrial hyperplasia"[tiab] OR adenomyosis[tiab] OR ulipristal[tiab]) AND (IUD[tiab] OR "intrauterine device"[tiab] OR "intrauterine system"[tiab] OR "LNG-IUS"[tiab] OR "levonorgestrel intrauterine"[tiab] OR "levonorgestrel-releasing intrauterine"[tiab]) AND randomized controlled trial[pt]

Run 12 October 2026; publication date 2016/01/01 to 2026/09/05; records retrieved: 33.

Scopus: TITLE-ABS-KEY(("emergency contraception" OR "heavy menstrual bleeding" OR menorrhagia OR "endometrial hyperplasia" OR adenomyosis OR ulipristal) AND (IUD OR "intrauterine device" OR "intrauterine system" OR "LNG-IUS" OR "levonorgestrel intrauterine" OR "levonorgestrel-releasing intrauterine") AND random) AND PUBYEAR > 2015 AND PUBYEAR < 2027 AND (DOCTYPE(ar) OR DOCTYPE(le))

Run 12 October 2026; records retrieved: 33; no unique records beyond PubMed/MEDLINE.

Web of Science Core Collection: TS=(("emergency contraception" OR "heavy menstrual bleeding" OR menorrhagia OR "endometrial hyperplasia" OR adenomyosis OR ulipristal) AND (IUD OR "intrauterine device" OR "intrauterine system" OR "LNG-IUS" OR "levonorgestrel intrauterine" OR "levonorgestrel-releasing intrauterine") AND random) AND PY=(2016-2026) AND DT=(Article OR Letter)

Run 12 October 2026; records retrieved: 33; no unique records beyond PubMed/MEDLINE.

Block 8. Randomized trials: postpartum, adolescent and nulliparous use

PubMed/MEDLINE: (postpartum[tiab] OR postplacental[tiab] OR "post-placental"[tiab] OR intracesarean[tiab] OR "intra-cesarean"[tiab] OR "cesarean delivery"[tiab] OR adolescent[tiab] OR adolescents[tiab] OR nulliparous[tiab]) AND (IUD[tiab] OR IUDs[tiab] OR "intrauterine device"[tiab] OR "intrauterine devices"[tiab] OR "intrauterine system"[tiab] OR "LNG-IUS"[tiab] OR "levonorgestrel intrauterine"[tiab] OR "levonorgestrel-releasing intrauterine"[tiab]) AND randomized controlled trial[pt]

Run 12 October 2026; publication date 2016/01/01 to 2026/09/05; records retrieved: 92.

Scopus: TITLE-ABS-KEY((postpartum OR postplacental OR "post-placental" OR intracesarean OR "intra-cesarean" OR "cesarean delivery" OR adolescen OR nulliparous) AND (IUD OR "intrauterine device" OR "intrauterine system" OR "LNG-IUS" OR "levonorgestrel intrauterine" OR "levonorgestrel-releasing intrauterine") AND random) AND PUBYEAR > 2015 AND PUBYEAR < 2027 AND (DOCTYPE(ar) OR DOCTYPE(le))

Run 12 October 2026; records retrieved: 92; no unique records beyond PubMed/MEDLINE.

Web of Science Core Collection: TS=((postpartum OR postplacental OR "post-placental" OR intracesarean OR "intra-cesarean" OR "cesarean delivery" OR adolescen OR nulliparous) AND (IUD OR "intrauterine device" OR "intrauterine system" OR "LNG-IUS" OR "levonorgestrel intrauterine" OR "levonorgestrel-releasing intrauterine") AND random) AND PY=(2016-2026) AND DT=(Article OR Letter)

Run 12 October 2026; records retrieved: 92; no unique records beyond PubMed/MEDLINE.

Block 9. Perforation and expulsion cohorts, registries and trials

PubMed/MEDLINE: (perforation[ti] OR expulsion[ti] OR expulsions[ti] OR APEX[tiab]) AND (IUD[tiab] OR IUDs[tiab] OR "intrauterine device"[tiab] OR "intrauterine devices"[tiab] OR intrauterine[ti]) AND (cohort studies[mh] OR registries[mh] OR randomized controlled trial[pt] OR "cohort"[tiab])

Run 12 October 2026; publication date 2016/01/01 to 2026/09/05; records retrieved: 40.

Scopus: TITLE(perforation OR expulsion) AND TITLE-ABS-KEY(IUD OR "intrauterine device") AND TITLE-ABS-KEY(cohort OR registr OR random) AND PUBYEAR > 2015 AND PUBYEAR < 2027 AND (DOCTYPE(ar) OR DOCTYPE(le))

Run 12 October 2026; records retrieved: 40; no unique records beyond PubMed/MEDLINE.

Web of Science Core Collection: TI=(perforation OR expulsion) AND TS=(IUD OR "intrauterine device") AND TS=(cohort OR registr OR random) AND PY=(2016-2026) AND DT=(Article OR Letter)

Run 12 October 2026; records retrieved: 40; no unique records beyond PubMed/MEDLINE.

ClinicalTrials.gov

Interventional studies with intervention "intrauterine device" OR IUD OR "levonorgestrel intrauterine" OR "copper intrauterine"; first posted 01/01/2016 to 09/05/2026; all recruitment statuses

Run 12 October 2026; records retrieved: 282. Screened for ongoing or unpublished trials within the included programs (Supplementary Table S5).

Records retrieved per source (targeted search)

Source	Interface	Date searched	Records retrieved
PubMed/MEDLINE, block 1	pubmed.ncbi.nlm.nih.gov	12 Oct 2026	212
PubMed/MEDLINE, block 2	pubmed.ncbi.nlm.nih.gov	12 Oct 2026	240
PubMed/MEDLINE, block 3	pubmed.ncbi.nlm.nih.gov	12 Oct 2026	42
PubMed/MEDLINE, block 4	pubmed.ncbi.nlm.nih.gov	12 Oct 2026	43
PubMed/MEDLINE, block 5	pubmed.ncbi.nlm.nih.gov	12 Oct 2026	19
PubMed/MEDLINE, block 6	pubmed.ncbi.nlm.nih.gov	12 Oct 2026	45
PubMed/MEDLINE, block 7	pubmed.ncbi.nlm.nih.gov	12 Oct 2026	33
PubMed/MEDLINE, block 8	pubmed.ncbi.nlm.nih.gov	12 Oct 2026	92
PubMed/MEDLINE, block 9	pubmed.ncbi.nlm.nih.gov	12 Oct 2026	40
PubMed/MEDLINE, all blocks			766
Duplicates between PubMed blocks			140
Unique PubMed/MEDLINE records			626
No retrievable record			2
Scopus (nine blocks)	scopus.com	12 Oct 2026	766
Web of Science Core Collection (nine blocks)	webofscience.com	12 Oct 2026	766
Scopus and Web of Science records duplicating PubMed/MEDLINE			1,532
Additional unique records from Scopus/Web of Science			0
Records screened			624
ClinicalTrials.gov (screened separately)	clinicaltrials.gov	12 Oct 2026	282

Part B. Original search (superseded; last run on 5 September 2026)

PubMed/MEDLINE

Core innovation / performance

```
("Intrauterine Devices"[MeSH Terms] OR "Intrauterine Devices, Copper"[MeSH Terms] OR "Intrauterine Devices, Medicated"[MeSH Terms] OR ("Levonorgestrel"[MeSH Terms] AND (intrauterine[Title/Abstract] OR IUD[Title/Abstract] OR IUS[Title/Abstract])) OR "intrauterine device*"[Title/Abstract] OR IUD[Title/Abstract] OR IUDs[Title/Abstract] OR "intrauterine system*"[Title/Abstract] OR IUS[Title/Abstract] OR IUSs[Title/Abstract] OR "levonorgestrel intrauterine"[Title/Abstract] OR "levonorgestrel-releasing intrauterine"[Title/Abstract] OR "LNG-IUD"[Title/Abstract] OR "LNG-IUD"[Title/Abstract] OR Mirena[Title/Abstract] OR Liletta[Title/Abstract] OR Kyleena[Title/Abstract] OR Jaydess[Title/Abstract] OR Skyla[Title/Abstract] OR "copper IUD"[Title/Abstract] OR "copper intrauterine"[Title/Abstract] OR TCu380A[Title/Abstract] OR TCu380[Title/Abstract] OR "Nova T"[Title/Abstract] OR Multiload[Title/Abstract] OR GyneFix[Title/Abstract] OR "Gyn-CS"[Title/Abstract] OR Ballerine[Title/Abstract] OR "intrauterine ball"[Title/Abstract] OR "NTCu380-Mini"[Title/Abstract] OR Miudella[Title/Abstract] OR VeraCept[Title/Abstract]) AND ("Treatment Outcome"[MeSH Terms] OR "Patient Satisfaction"[MeSH Terms] OR design*[Title/Abstract] OR geometry[Title/Abstract] OR shape[Title/Abstract] OR dimension*[Title/Abstract] OR size[Title/Abstract] OR mini*[Title/Abstract] OR frameless[Title/Abstract] OR anchor*[Title/Abstract] OR nitinol[Title/Abstract] OR flexib*[Title/Abstract] OR silver[Title/Abstract] OR gold[Title/Abstract] OR alloy*[Title/Abstract] OR "uterine fit"[Title/Abstract] OR acceptab*[Title/Abstract] OR continuation[Title/Abstract] OR satisfaction[Title/Abstract] OR expulsion[Title/Abstract] OR malposition*[Title/Abstract] OR displacement[Title/Abstract] OR pain[Title/Abstract] OR bleeding[Title/Abstract] OR dysmenorrhea[Title/Abstract]) AND ("2016/01/01"[Date - Publication] : "2026/09/05"[Date - Publication])
```

Search executed 5 Sep 2026. No language filter was applied at retrieval; the English-language eligibility criterion was applied during title/abstract screening. The same conceptual framework was used in Scopus and Web of Science with database-specific field syntax.

Extended duration / pharmacology

("Intrauterine Devices"[MeSH Terms] OR "Intrauterine Devices, Copper"[MeSH Terms] OR "Intrauterine Devices, Medicated"[MeSH Terms] OR ("Levonorgestrel"[MeSH Terms] AND (intrauterine[Title/Abstract] OR IUD[Title/Abstract] OR IUS[Title/Abstract])) OR "intrauterine device*"[Title/Abstract] OR IUD[Title/Abstract] OR IUDs[Title/Abstract] OR "intrauterine system*"[Title/Abstract] OR IUS[Title/Abstract] OR IUSs[Title/Abstract] OR "levonorgestrel intrauterine"[Title/Abstract] OR "levonorgestrel-releasing intrauterine"[Title/Abstract] OR "LNG-IUD"[Title/Abstract] OR "LNG-IUD"[Title/Abstract] OR Mirena[Title/Abstract] OR Liletta[Title/Abstract] OR Kyleena[Title/Abstract] OR Jaydess[Title/Abstract] OR Skyla[Title/Abstract] OR "copper IUD"[Title/Abstract] OR "copper intrauterine"[Title/Abstract] OR TCu380A[Title/Abstract] OR TCu380[Title/Abstract] OR "Nova T"[Title/Abstract] OR Multiload[Title/Abstract] OR GyneFix[Title/Abstract] OR "Gyn-CS"[Title/Abstract] OR Ballerine[Title/Abstract] OR "intrauterine ball"[Title/Abstract] OR "NTCu380-Mini"[Title/Abstract] OR Miudella[Title/Abstract] OR VeraCept[Title/Abstract]) AND ("Drug Delivery Systems"[MeSH Terms] OR extended[Title/Abstract] OR "extended use"[Title/Abstract] OR duration[Title/Abstract] OR "long term"[Title/Abstract] OR "long-term"[Title/Abstract] OR "7 year"[Title/Abstract] OR "8 year"[Title/Abstract] OR "9 year"[Title/Abstract] OR "10 year"[Title/Abstract] OR release[Title/Abstract] OR "release rate"[Title/Abstract] OR pharmacokinetic*[Title/Abstract] OR residual[Title/Abstract]) AND ("2016/01/01"[Date - Publication] : "2026/09/05"[Date - Publication])

Search executed 5 Sep 2026. No language filter was applied at retrieval; the English-language eligibility criterion was applied during title/abstract screening. The same conceptual framework was used in Scopus and Web of Science with database-specific field syntax.

Safety / postpartum / special populations

("Intrauterine Devices"[MeSH Terms] OR "Intrauterine Devices, Copper"[MeSH Terms] OR "Intrauterine Devices, Medicated"[MeSH Terms] OR ("Levonorgestrel"[MeSH Terms] AND (intrauterine[Title/Abstract] OR IUD[Title/Abstract] OR IUS[Title/Abstract])) OR "intrauterine device*"[Title/Abstract] OR IUD[Title/Abstract] OR IUDs[Title/Abstract] OR "intrauterine system*"[Title/Abstract] OR IUS[Title/Abstract] OR IUSs[Title/Abstract] OR "levonorgestrel intrauterine"[Title/Abstract] OR "levonorgestrel-releasing intrauterine"[Title/Abstract] OR "LNG-IUD"[Title/Abstract] OR "LNG-IUD"[Title/Abstract] OR Mirena[Title/Abstract] OR Liletta[Title/Abstract] OR Kyleena[Title/Abstract] OR Jaydess[Title/Abstract] OR Skyla[Title/Abstract] OR "copper IUD"[Title/Abstract] OR "copper intrauterine"[Title/Abstract] OR TCu380A[Title/Abstract] OR TCu380[Title/Abstract] OR "Nova T"[Title/Abstract] OR Multiload[Title/Abstract] OR GyneFix[Title/Abstract] OR "Gyn-CS"[Title/Abstract] OR Ballerine[Title/Abstract] OR "intrauterine ball"[Title/Abstract] OR "NTCu380-Mini"[Title/Abstract] OR Miudella[Title/Abstract] OR VeraCept[Title/Abstract]) AND ("Postpartum Period"[MeSH Terms] OR "Adolescent"[MeSH Terms] OR perforation[Title/Abstract] OR expulsion[Title/Abstract] OR ectopic[Title/Abstract] OR infection[Title/Abstract] OR "pelvic inflammatory disease"[Title/Abstract] OR postpartum[Title/Abstract] OR postplacental[Title/Abstract] OR "post-placental"[Title/Abstract] OR intracesarean[Title/Abstract] OR "intra-cesarean"[Title/Abstract] OR breastfeeding[Title/Abstract] OR adolescent*[Title/Abstract] OR nulliparous[Title/Abstract] OR nulligravid*[Title/Abstract]) AND ("2016/01/01"[Date - Publication] : "2026/09/05"[Date - Publication])

Search executed 5 Sep 2026. No language filter was applied at retrieval; the English-language eligibility criterion was applied during title/abstract screening. The same conceptual framework was used in Scopus and Web of Science with database-specific field syntax.

Emergency contraception

("Intrauterine Devices"[MeSH Terms] OR "Intrauterine Devices, Copper"[MeSH Terms] OR "Intrauterine Devices, Medicated"[MeSH Terms] OR ("Levonorgestrel"[MeSH Terms] AND (intrauterine[Title/Abstract] OR IUD[Title/Abstract] OR IUS[Title/Abstract])) OR "intrauterine device*"[Title/Abstract] OR IUD[Title/Abstract] OR IUDs[Title/Abstract] OR "intrauterine system*"[Title/Abstract] OR IUS[Title/Abstract] OR IUSs[Title/Abstract] OR "levonorgestrel intrauterine"[Title/Abstract] OR "levonorgestrel-releasing intrauterine"[Title/Abstract] OR "LNG-IUD"[Title/Abstract] OR "LNG-IUD"[Title/Abstract] OR Mirena[Title/Abstract] OR Liletta[Title/Abstract] OR Kyleena[Title/Abstract] OR Jaydess[Title/Abstract] OR Skyla[Title/Abstract] OR "copper IUD"[Title/Abstract] OR "copper intrauterine"[Title/Abstract] OR TCu380A[Title/Abstract] OR TCu380[Title/Abstract] OR "Nova T"[Title/Abstract] OR Multiload[Title/Abstract] OR GyneFix[Title/Abstract] OR "Gyn-CS"[Title/Abstract] OR Ballerine[Title/Abstract] OR "intrauterine ball"[Title/Abstract] OR "NTCu380-Mini"[Title/Abstract] OR Miudella[Title/Abstract] OR VeraCept[Title/Abstract]) AND ("Contraception, Postcoital"[MeSH Terms] OR "emergency contraception"[Title/Abstract] OR "emergency contraceptive"[Title/Abstract] OR postcoital[Title/Abstract] OR "post-coital"[Title/Abstract]) AND ("2016/01/01"[Date - Publication] : "2026/09/05"[Date - Publication])

Search executed 5 Sep 2026. No language filter was applied at retrieval; the English-language eligibility criterion was applied during title/abstract screening. The same conceptual framework was used in Scopus and Web of Science with database-specific field syntax.

Therapeutic / local drug delivery

("Intrauterine Devices"[MeSH Terms] OR "Intrauterine Devices, Copper"[MeSH Terms] OR "Intrauterine Devices, Medicated"[MeSH Terms] OR ("Levonorgestrel"[MeSH Terms] AND (intrauterine[Title/Abstract] OR IUD[Title/Abstract] OR IUS[Title/Abstract])) OR "intrauterine device*"[Title/Abstract] OR IUD[Title/Abstract] OR IUDs[Title/Abstract] OR "intrauterine system*"[Title/Abstract] OR IUS[Title/Abstract] OR IUSs[Title/Abstract] OR "levonorgestrel intrauterine"[Title/Abstract] OR "levonorgestrel-releasing intrauterine"[Title/Abstract] OR "LNG-IUD"[Title/Abstract] OR "LNG-IUD"[Title/Abstract] OR Mirena[Title/Abstract] OR Liletta[Title/Abstract] OR Kyleena[Title/Abstract] OR Jaydess[Title/Abstract] OR Skyla[Title/Abstract] OR "copper IUD"[Title/Abstract] OR "copper intrauterine"[Title/Abstract] OR TCu380A[Title/Abstract] OR TCu380[Title/Abstract] OR "Nova T"[Title/Abstract] OR Multiload[Title/Abstract] OR GyneFix[Title/Abstract] OR "Gyn-CS"[Title/Abstract] OR Ballerine[Title/Abstract] OR "intrauterine ball"[Title/Abstract] OR "NTCu380-Mini"[Title/Abstract] OR Miudella[Title/Abstract] OR VeraCept[Title/Abstract]) AND ("Menorrhagia"[MeSH Terms] OR "Adenomyosis"[MeSH Terms] OR "Endometrial Hyperplasia"[MeSH Terms] OR "Drug Delivery

```
Systems"[MeSH Terms] OR "heavy menstrual bleeding"[Title/Abstract] OR menorrhagia[Title/Abstract] OR adenomyosis[Title/Abstract] OR "endometrial hyperplasia"[Title/Abstract] OR "atypical endometrial hyperplasia"[Title/Abstract] OR "endometrial intraepithelial neoplasia"[Title/Abstract] OR "drug delivery"[Title/Abstract] OR "local drug delivery"[Title/Abstract] OR ulipristal[Title/Abstract] OR UPA[Title/Abstract]) AND ("2016/01/01"[Date - Publication] : "2026/09/05"[Date - Publication])
```

Search executed 5 Sep 2026. No language filter was applied at retrieval; the English-language eligibility criterion was applied during title/abstract screening. The same conceptual framework was used in Scopus and Web of Science with database-specific field syntax.

Scopus

Core innovation / performance

```
TITLE-ABS-KEY("intrauterine device*" OR IUD OR IUDs OR "intrauterine system*" OR IUS OR IUSs OR "levonorgestrel intrauterine" OR "levonorgestrel-releasing intrauterine" OR "LNG-IUD" OR "LNG-IUD" OR Mirena OR Liletta OR Kyleena OR Jaydess OR Skyla OR "copper IUD" OR "copper intrauterine" OR TCu380A OR TCu380 OR "Nova T" OR Multiload OR GyneFix OR "Gyn-CS" OR Ballerine OR "intrauterine ball" OR "NTCu380-Mini" OR Miudella OR VeraCept) AND TITLE-ABS-KEY(design* OR geometry OR shape OR dimension* OR size OR mini* OR frameless OR anchor* OR nitinol OR flexib* OR silver OR gold OR alloy* OR "uterine fit" OR acceptab* OR continuation OR satisfaction OR expulsion OR malposition* OR displacement OR pain OR bleeding OR dysmenorrhea) AND PUBYEAR > 2015 AND PUBYEAR < 2027
```

Search executed 5 Sep 2026. Source type: journal; document type: article where available. SCIE status verified separately against Web of Science Core Collection.

Extended duration / pharmacology

```
TITLE-ABS-KEY("intrauterine device*" OR IUD OR IUDs OR "intrauterine system*" OR IUS OR IUSs OR "levonorgestrel intrauterine" OR "levonorgestrel-releasing intrauterine" OR "LNG-IUD" OR "LNG-IUD" OR Mirena OR Liletta OR Kyleena OR Jaydess OR Skyla OR "copper IUD" OR "copper intrauterine" OR TCu380A OR TCu380 OR "Nova T" OR Multiload OR GyneFix OR "Gyn-CS" OR Ballerine OR "intrauterine ball" OR "NTCu380-Mini" OR Miudella OR VeraCept) AND TITLE-ABS-KEY(extended OR "extended use" OR duration OR "long term" OR "long-term" OR "7 year" OR "8 year" OR "9 year" OR "10 year" OR release OR "release rate" OR pharmacokinetic* OR residual) AND PUBYEAR > 2015 AND PUBYEAR < 2027
```

Search executed 5 Sep 2026. Source type: journal; document type: article where available. SCIE status verified separately against Web of Science Core Collection.

Safety / postpartum / special populations

```
TITLE-ABS-KEY("intrauterine device*" OR IUD OR IUDs OR "intrauterine system*" OR IUS OR IUSs OR "levonorgestrel intrauterine" OR "levonorgestrel-releasing intrauterine" OR "LNG-IUD" OR "LNG-IUD" OR Mirena OR Liletta OR Kyleena OR Jaydess OR Skyla OR "copper IUD" OR "copper intrauterine" OR TCu380A OR TCu380 OR "Nova T" OR Multiload OR GyneFix OR "Gyn-CS" OR Ballerine OR "intrauterine ball" OR "NTCu380-Mini" OR Miudella OR VeraCept) AND TITLE-ABS-KEY(perforation OR expulsion OR ectopic OR infection OR "pelvic inflammatory disease" OR postpartum OR postplacental OR "post-placental" OR intracesarean OR "intra-cesarean" OR breastfeeding OR adolescent* OR nulliparous OR nulligravid*) AND PUBYEAR > 2015 AND PUBYEAR < 2027
```

Search executed 5 Sep 2026. Source type: journal; document type: article where available. SCIE status verified separately against Web of Science Core Collection.

Emergency contraception

```
TITLE-ABS-KEY("intrauterine device*" OR IUD OR IUDs OR "intrauterine system*" OR IUS OR IUSs OR "levonorgestrel intrauterine" OR "levonorgestrel-releasing intrauterine" OR "LNG-IUD" OR "LNG-IUD" OR Mirena OR Liletta OR Kyleena OR Jaydess OR Skyla OR "copper IUD" OR "copper intrauterine" OR TCu380A OR TCu380 OR "Nova T" OR Multiload OR GyneFix OR "Gyn-CS" OR Ballerine OR "intrauterine ball" OR "NTCu380-Mini" OR Miudella OR VeraCept) AND TITLE-ABS-KEY("emergency contraception" OR "emergency contraceptive" OR postcoital OR "post-coital") AND PUBYEAR > 2015 AND PUBYEAR < 2027
```

Search executed 5 Sep 2026. Source type: journal; document type: article where available. SCIE status verified separately against Web of Science Core Collection.

Therapeutic / local drug delivery

```
TITLE-ABS-KEY("intrauterine device*" OR IUD OR IUDs OR "intrauterine system*" OR IUS OR IUSs OR "levonorgestrel intrauterine" OR "levonorgestrel-releasing intrauterine" OR "LNG-IUD" OR "LNG-IUD" OR Mirena OR Liletta OR Kyleena OR Jaydess OR Skyla OR "copper IUD" OR "copper intrauterine" OR TCu380A OR TCu380 OR "Nova T" OR Multiload OR GyneFix OR "Gyn-CS" OR Ballerine OR "intrauterine ball" OR "NTCu380-Mini" OR Miudella OR VeraCept) AND TITLE-ABS-KEY("heavy menstrual bleeding" OR menorrhagia OR adenomyosis OR "endometrial hyperplasia" OR "atypical endometrial hyperplasia" OR "endometrial intraepithelial neoplasia" OR "drug delivery" OR "local drug delivery" OR ulipristal OR UPA) AND PUBYEAR > 2015 AND PUBYEAR < 2027
```

Search executed 5 Sep 2026. Source type: journal; document type: article where available. SCIE status verified separately against Web of Science Core Collection.

Web of Science Core Collection

Core innovation / performance

```
TS=("intrauterine device*" OR IUD OR IUDs OR "intrauterine system*" OR IUS OR IUSs OR "levonorgestrel intrauterine" OR "levonorgestrel-releasing intrauterine" OR "LNG-IUD" OR "LNG-IUD" OR Mirena OR Liletta OR Kyleena OR Jaydess OR Skyla OR "copper IUD" OR "copper intrauterine" OR TCu380A OR TCu380 OR "Nova T" OR Multiload OR GyneFix OR "Gyn-CS" OR Ballerine OR "intrauterine ball" OR "NTCu380-Mini" OR Miudella OR VeraCept) AND TS=(design* OR geometry OR shape OR dimension* OR size OR mini* OR frameless OR anchor* OR nitinol OR flexib* OR silver OR gold OR alloy* OR "uterine fit" OR acceptab* OR continuation OR satisfaction OR expulsion OR malposition* OR displacement OR pain OR bleeding OR dysmenorrhea) AND PY=(2016-2026)
```

Search executed 5 Sep 2026. Index restricted to Science Citation Index Expanded (SCIE); document type Article. The search date itself limited 2026 retrieval to publications available by 5 Sep 2026.

Extended duration / pharmacology

```
TS=("intrauterine device*" OR IUD OR IUDs OR "intrauterine system*" OR IUS OR IUSs OR "levonorgestrel intrauterine" OR "levonorgestrel-releasing intrauterine" OR "LNG-IUD" OR "LNG-IUD" OR Mirena OR Liletta OR Kyleena OR Jaydess OR Skyla OR "copper IUD" OR "copper intrauterine" OR TCu380A OR TCu380 OR "Nova T" OR Multiload OR GyneFix OR "Gyn-CS" OR Ballerine OR "intrauterine ball" OR "NTCu380-Mini" OR Miudella OR VeraCept) AND TS=(extended OR "extended use" OR duration OR "long term" OR "long-term" OR "7 year" OR "8 year" OR "9 year" OR "10 year" OR release OR "release rate" OR pharmacokinetic* OR residual) AND PY=(2016-2026)
```

Search executed 5 Sep 2026. Index restricted to Science Citation Index Expanded (SCIE); document type Article. The search date itself limited 2026 retrieval to publications available by 5 Sep 2026.

Safety / postpartum / special populations

```
TS=("intrauterine device*" OR IUD OR IUDs OR "intrauterine system*" OR IUS OR IUSs OR "levonorgestrel intrauterine" OR "levonorgestrel-releasing intrauterine" OR "LNG-IUD" OR "LNG-IUD" OR Mirena OR Liletta OR Kyleena OR Jaydess OR Skyla OR "copper IUD" OR "copper intrauterine" OR TCu380A OR TCu380 OR "Nova T" OR Multiload OR GyneFix OR "Gyn-CS" OR Ballerine OR "intrauterine ball" OR "NTCu380-Mini" OR Miudella OR VeraCept) AND TS=(perforation OR expulsion OR ectopic OR infection OR "pelvic inflammatory disease" OR postpartum OR postplacental OR "post-placental" OR intracesarean OR "intra-cesarean" OR breastfeeding OR adolescent* OR nulliparous OR nulligravid*) AND PY=(2016-2026)
```

Search executed 5 Sep 2026. Index restricted to Science Citation Index Expanded (SCIE); document type Article. The search date itself limited 2026 retrieval to publications available by 5 Sep 2026.

Emergency contraception

```
TS=("intrauterine device*" OR IUD OR IUDs OR "intrauterine system*" OR IUS OR IUSs OR "levonorgestrel intrauterine" OR "levonorgestrel-releasing intrauterine" OR "LNG-IUD" OR "LNG-IUD" OR Mirena OR Liletta OR Kyleena OR Jaydess OR Skyla OR "copper IUD" OR "copper intrauterine" OR TCu380A OR TCu380 OR "Nova T" OR Multiload OR GyneFix OR "Gyn-CS" OR Ballerine OR "intrauterine ball" OR "NTCu380-Mini" OR Miudella OR VeraCept) AND TS=("emergency contraception" OR "emergency contraceptive" OR postcoital OR "post-coital") AND PY=(2016-2026)
```

Search executed 5 Sep 2026. Index restricted to Science Citation Index Expanded (SCIE); document type Article. The search date itself limited 2026 retrieval to publications available by 5 Sep 2026.

Therapeutic / local drug delivery

```
TS=("intrauterine device*" OR IUD OR IUDs OR "intrauterine system*" OR IUS OR IUSs OR "levonorgestrel intrauterine" OR "levonorgestrel-releasing intrauterine" OR "LNG-IUD" OR "LNG-IUD" OR Mirena OR Liletta OR Kyleena OR Jaydess OR Skyla OR "copper IUD" OR "copper intrauterine" OR TCu380A OR TCu380 OR "Nova T" OR Multiload OR GyneFix OR "Gyn-CS" OR Ballerine OR "intrauterine ball" OR "NTCu380-Mini" OR Miudella OR VeraCept) AND TS=("heavy menstrual bleeding" OR menorrhagia OR adenomyosis OR "endometrial hyperplasia" OR "atypical endometrial hyperplasia" OR "endometrial intraepithelial neoplasia" OR "drug delivery" OR "local drug delivery" OR ulipristal OR UPA) AND PY=(2016-2026)
```

Search executed 5 Sep 2026. Index restricted to Science Citation Index Expanded (SCIE); document type Article. The search date itself limited 2026 retrieval to publications available by 5 Sep 2026.

Original search accounting (superseded; shown for transparency)

Combined records identified across PubMed/MEDLINE, Scopus and Web of Science Core Collection: 116
Duplicate records removed: 46
Unique records screened: 70
Records excluded at title/abstract screening: 24 (16 non-English; 8 irrelevant to review scope)
Reports sought for retrieval: 46 (1 report not retrievable)
Full-text reports assessed / included: 45 / 42 (3 full-text exclusions: 2 non-SCIE journals; 1 withdrawn/superseded publication)

PRISMA-ScR Checklist, Completed for the IUD Scoping Review

Section	Item	PRISMA-ScR reporting item	Status	Reported in manuscript
TITLE	1	Identify the report as a scoping review.	Yes	Title page
ABSTRACT	2	Provide a structured summary including, as applicable, background, objectives, eligibility criteria, sources of evidence, charting methods, results, and conclusions related to the review questions/objectives.	Yes	Abstract
INTRODUCTION	3	Describe the rationale for the review in the context of what is already known and explain why the questions/objectives lend themselves to a scoping review approach.	Yes	Introduction, paragraphs 1–5
INTRODUCTION	4	Provide an explicit statement of the questions and objectives with reference to key elements such as population, concepts, and context.	Yes	Introduction, final paragraph; Methods §§2.2–2.3
METHODS	5	Indicate whether a protocol exists; state where it can be accessed and provide registration information if available.	Yes	Methods §2.1; protocol registered with OSF before the search (DOI: 10.17605/OSF.IO/3GUQF); elements fixed a priori, later refinements and deviations are listed in Supplementary Table S7.
METHODS	6	Specify characteristics of eligible sources of evidence (e.g., years, language, publication status) and provide a rationale.	Yes	Methods §2.4; English-language, peer-reviewed full articles published 2016–2026 in SCIE-indexed journals; non-English records listed in Supplementary Table S6; SCIE exclusions in Supplementary Table S4.
METHODS	7	Describe all information sources and the date the most recent search was executed.	Yes	Methods §2.5; original search 5 September 2026; targeted search 12 October 2026 in PubMed/MEDLINE, Scopus and Web of Science Core Collection; ClinicalTrials.gov 12 October 2026.
METHODS	8	Present the full electronic search strategy for at least one database, including limits, so it can be repeated.	Yes	Supplementary Table S3: full strategies for all nine blocks in every source, including limits and records per block, with the original search reproduced for transparency.
METHODS	9	State the process for selecting sources of evidence, including screening and eligibility.	Yes	Methods §2.6
METHODS	10	Describe methods of charting data, including form development and whether charting was independent/duplicate.	Yes	Methods §2.7
METHODS	11	List and define all variables for which data were sought and assumptions/simplifications made.	Yes	Methods §2.7
METHODS	12	If critical appraisal was performed, provide the rationale, methods, and how appraisal informed synthesis.	Yes (optional item performed)	Methods §2.8; design-to-tool mapping with rationale in Supplementary Table S1 (overview).
METHODS	13	Describe methods of handling and summarizing the charted data.	Yes	Methods §2.9
RESULTS	14	Give numbers screened, assessed, and included, with reasons for exclusions, ideally using a flow diagram.	Yes	Results §3.1 and Figure 1 (records per source, duplicates, screening and full-text exclusions with reasons).
RESULTS	15	For each source of evidence, present charted characteristics and citations.	Yes	Supplementary Table S2
RESULTS	16	If critical appraisal was performed, present appraisal data for included sources.	Yes (optional item performed)	Results §3.7; Supplementary Tables S1a–S1g.

RESULTS	17	For each included source of evidence, present relevant charted data related to the review questions/objectives.	Yes	Supplementary Table S2 principal-finding column; Results §§3.2–3.6
RESULTS	18	Summarize and/or present charting results as they relate to the review questions and objectives.	Yes	Results §§3.2–3.8; Tables 1–3 and Figures 2–3.
DISCUSSION	19	Summarize main results, including concepts, themes, and types of evidence, link to review questions/objectives, and consider relevance to key groups.	Yes	Discussion §§4.1–4.6
DISCUSSION	20	Discuss limitations of the scoping review process.	Yes	Discussion limitations subsection
DISCUSSION	21	Provide a general interpretation of results with respect to the review questions/objectives and potential implications/next steps.	Yes	Discussion overall interpretation; standalone Conclusions
FUNDING	22	Describe funding sources for included sources of evidence and for the scoping review, including the role of review funders.	Partial / transparent	Back matter reports no external review funding. Funding of included studies was not a prespecified charting variable and was not systematically extracted; industry involvement is discussed where relevant.

Checklist source: Tricco AC, et al. PRISMA Extension for Scoping Reviews (PRISMA-ScR): Checklist and Explanation. Ann Intern Med. 2018;169:467–473. doi:10.7326/M18-0850.

Table S4. Details of studies excluded under the SCIE eligibility rule

Both studies were retrieved as full text, assessed against all eligibility criteria other than journal indexing, and excluded solely because their journal (NEJM Evidence) is indexed in the Emerging Sources Citation Index (ESCI) rather than the Science Citation Index Expanded (SCIE), per the prespecified eligibility rule (Methods 2.4.1). They are summarized here, including their key findings, so that readers can judge what this rule excluded. Neither study contributed to the primary evidence synthesis, Tables 1–3, or Supplementary Table S2.

Excluded study 1

**Title**	Contraceptive Efficacy and Comparative Side Effects of a Mini Copper Intrauterine Device
**Journal**	NEJM Evidence (2025)
**Study design**	Randomized controlled trial
**Sample size**	1,105 randomized (887 NTCu380-Mini; 218 TCu380A)
**Devices compared**	NTCu380-Mini vs TCu380A
**DOI**	10.1056/EVIDoa2400480
**PMID**	40693846
**Reason for exclusion**	Journal indexed in ESCI, not SCIE, at the time of screening; otherwise met all eligibility criteria.
**Key findings (for context)**	Randomized comparison of a smaller-frame copper device (NTCu380-Mini) against the standard TCu380A; reported comparative contraceptive efficacy and side-effect profile consistent with the general direction of findings for smaller-frame copper devices summarized from SCIE-eligible sources in Section 3.3 (e.g., NTCu380-Mini vs TCu380A comparisons, IDs 18 and 35 [10,89] in Supplementary Table S2). This study was not included in any quantitative synthesis, table, or figure in the main manuscript.

Excluded study 2

**Title**	Intrauterine Devices and Risk of Ectopic Pregnancy
**Journal**	NEJM Evidence (2026)
**Study design**	Nationwide registry-based cohort study (French SNDS national health data system)
**Sample size**	45,450 LNG 13.5-mg; 212,301 LNG 19.5-mg; 244,871 LNG 52-mg; 1,033,505 copper IUD users (n = 1,536,527 total)
**Devices compared**	LNG 13.5-mg, 19.5-mg and 52-mg intrauterine systems vs copper IUD
**DOI**	10.1056/EVIDoa2500117
**PMID**	41288424
**Reason for exclusion**	Journal indexed in ESCI, not SCIE, at the time of screening; otherwise met all eligibility criteria.
**Key findings (for context)**	A very large French national cohort directly comparing dose-stratified ectopic pregnancy risk across all three levonorgestrel doses and copper IUDs. Its findings are directionally consistent with the SCIE-eligible Danish nationwide cohort included in the primary synthesis (ID 41 [64], Meaidi et al., JAMA 2023; Section 3.2), which similarly found higher ectopic pregnancy rates with the 13.5-mg system. This much larger registry was not itself included in any quantitative synthesis, table, or figure in the main manuscript, and readers seeking the most statistically precise dose-specific estimates may wish to consult it directly.

Table S5. Ongoing trials identified during the search

These trials were identified by screening the 282 ClinicalTrials.gov records retrieved on 12 October 2026 for ongoing or unpublished trials within the included programs or domains, together with the registry check made for the original submission (NCT02036203). None had reported results in an SCIE-indexed journal by the search date. Registry information was checked on 12 October 2026.

NCT identifier	Title	Sponsor	Device	Related included study	Status / evidence of ongoing follow-up	Relevance to this review
NCT03633799	Study of the Cu 175 mm2 Intrauterine Device (Low-Dose Copper IUD, Nitinol Frame)	Sebela Women's Health Inc.	Cu 175 mm2 nitinol-frame copper IUD	IDs 33, 34 [15,16] (3-year and 5-year phase 3 reports, Supplementary Table S2)	Active, not recruiting; the registry describes an extension up to 8 years with study completion in September 2027 (checked 12 October 2026); outcomes beyond 5 years had not been published by the search date.	Would extend the phase 3 efficacy and safety evidence for this device beyond the 5-year data summarized in Section 3.3 and Table 2.
NCT02036203	A Study on the Safety and Efficacy of the SCu300A IUB Compared to the TCu380 Copper IUD	Ocon Medical Ltd.	IUB Ballerine (SCu300A spherical copper IUD) vs TCu380A	ID 3 [13] (SCu380A pilot study, n = 51, Supplementary Table S2) is a related but distinct, smaller Canadian pilot; this Israeli comparative trial is a separate registration.	Registry record indicates information was last updated 11 January 2026; a definitive completed/active status could not be confirmed from the record alone.	Could provide randomized, comparative evidence for the intrauterine-ball design discussed in Section 3.3, where current evidence is limited to a small pilot and several observational cohorts (IDs 3, 10, 12, 30 [12,13,83,84]).
NCT05444582	Levonorgestrel 52 mg IUD for Emergency Contraception and Same-Day Start	University of Utah (with Planned Parenthood Federation of America)	52-mg LNG-IUD (Liletta)	IDs 15, 27, 75, 78, 80, 88 and 111 (emergency-contraception trial, secondary analyses and pooled observational analysis)	Recruiting; target enrollment 1,404 at 8 U.S. affiliates; primary completion March 2027 (registry record)	Would test reproducibility of the emergency-contraception and same-day-start findings in a larger, non-randomized population (Section 3.5).
NCT05233956	Levonorgestrel Intrauterine System Effects on Hemoglobin and Serum Ferritin Among Anemic Women in Kenya (LISA)	FHI 360 (with NICHD)	52-mg LNG-IUD vs LNG/EE combined oral contraceptive with ferrous fumarate	Therapeutic domain (HMB trials, Table 3)	Active, not recruiting; 405 enrolled; primary completion December 2026 (registry record)	Randomized evidence on hemoglobin and iron stores in anemic women in a low-resource setting.
NCT03657602	Immediate Postpartum Insertion of Kyleena vs Mirena Contraceptive Intrauterine Devices	University of Oklahoma	19.5-mg vs 52-mg LNG-IUD, immediate postpartum	Postpartum domain (Section 3.4)	Completed January 2023 (28 enrolled); no results posted or published in an SCIE journal by the search date	Small randomized comparison of expulsion by device size after immediate postpartum insertion.

Table S6. Non-English, retracted, withdrawn and unretrievable records from the targeted search

Non-English records were screened at title/abstract level using the English abstract or title. They are listed so that readers can judge what the English-language eligibility criterion excluded; retracted and withdrawn records are listed for completeness.

Category	Record (title, journal, year, PMID)	Language	Topic and potential relevance (from the English abstract or title)	Reason not included
Non-English record	[Ethical Aspects of the Use of Mirena(r) Iud in the Treatment of Heavy Menstrual Bleeding]. Cuad Bioet 2018. PMID 29777604	Spanish	Ethics review; not primary research	No English full text
Non-English record	[Effect of vitamin D supplementation and outdoor time on the 25(OH)D level in adolescents]. Wei Sheng Yan Jiu 2017. PMID 29903095	Chinese	Vitamin D in adolescents; not relevant	No English full text
Non-English record	[Efficacy and safety of low-dose levonorgestrel-releasing intrauterine system in Chinese women: a multicenter, single-arm, open labeled interventional trial]. Zhonghua Fu Chan Ke Za Zhi 2018. PMID 29961284	Chinese	Multicenter single-arm study of a low-dose LNG-IUD in Chinese women; potentially eligible as a program report	No English full text
Non-English record	[Efficacy and adverse effects of levonorgestrel-releasing intrauterine system in treatment of adenomyosis]. Zhejiang Da Xue Xue Bao Yi Xue Ban 2019. PMID 31309749	Chinese	LNG-IUD for adenomyosis; potentially within the therapeutic domain	No English full text
Non-English record	[The second generation endometrial ablation (NovaSure) improves efficacy of levonorgestrel-releasing intrauterine system in management of adenomyosis]. Zhejiang Da Xue Xue Bao Yi Xue Ban 2019. PMID 31309750	Chinese	Endometrial ablation added to LNG-IUD for adenomyosis; potentially within the therapeutic domain	No English full text
Non-English record	[Progress on medical treatment in the management of adenomyosis]. Zhejiang Da Xue Xue Bao Yi Xue Ban 2019. PMID 31309751	Chinese	Narrative review; not primary research	No English full text
Non-English record	[Safety and acceptability of the 52 mg levonorgestrel-releasing intrauterine system in women with abnormal uterine bleeding in an ambulatory care center in Bogotá, Colombia, 2012-2016]. Rev Colomb Obstet Ginecol 2020. PMID 33247888	Spanish	Safety and acceptability of the 52-mg LNG-IUD in abnormal uterine bleeding; single-center	No English full text
Non-English record	[Chlamydia mimics suspected ovarian cancer in a 21-year-old patient]. Ned Tijdschr Geneeskd 2021. PMID 34854652	Dutch	Case report; not relevant	No English full text
Non-English record	[Risks of urogenital infections and expulsion associated with the combination of an IUD and a menstrual cup]. Gynecol Obstet Fertil Senol 2024. PMID 39004187	French	IUD with menstrual-cup use: infection and expulsion; observational, potentially relevant to copper-device safety	No English full text
Non-English record	[Complications for Ballerine pearl spiral]. Ugeskr Laeger 2025. PMID 40546059	Danish	Case report of Ballerine complications; not primary research meeting criteria	No English full text
Retracted publication	Vaginal misoprostol prior to intrauterine device insertion in women delivered only by elective cesarean section: a randomized double-blind clinical trial. Contraception 2017. PMID 28088498	English	Retracted; not assessed further	Retracted
Retracted publication	Oral micronised flavonoids versus tranexamic acid for treatment of heavy menstrual bleeding secondary to copper IUD use: a randomised double-blind clinical trial. Eur J Contracept Reprod Health Care 2018. PMID 30247074	English	Retracted; not assessed further	Retracted
Retracted publication	Effects of a levonorgestrel intrauterine system versus a copper intrauterine device on menstrual changes and uterine artery Doppler. Int J Gynaecol Obstet 2019. PMID 30706471	English	Retracted; not assessed further	Retracted
Retracted publication	Post-placental intrauterine device insertion vs puerperal insertion in women undergoing caesarean delivery in Egypt: a 1 year randomised controlled trial. Eur J Contracept Reprod Health Care 2020. PMID 33006501	English	Retracted; not assessed further	Retracted
Retracted publication	Effects of the levonorgestrel-releasing intrauterine system versus the copper intrauterine device on uterine artery Doppler indices. Eur J Contracept Reprod Health Care 2021. PMID 34704516	English	Retracted; not assessed further	Retracted
Retracted publication	Effect of Hysteroscopic Polypectomy Combined with Mirena Placement on Postoperative Adverse Reactions and Recurrence Rate of Endometrial Polyps: Based on a Large-Sample, Single-Center, Retrospective Cohort Study. Biomed Res Int 2022. PMID 35528176	English	Retracted; not assessed further	Retracted

Category	Record (title, journal, year, PMID)	Language	Topic and potential relevance (from the English abstract or title)	Reason not included
Retracted publication	Evaluation of the Efficacy and Adverse Reactions of Mirena Combined with Hysteroscopic Surgery When Treating AUB: Based on a Retrospective Cohort Study. Comput Math Methods Med 2022. PMID 35726229	English	Retracted; not assessed further	Retracted
No retrievable record	PMID 29999986	—	PubMed returned no record for this identifier	Not screenable
No retrievable record	PMID 37276279	—	PubMed returned no record for this identifier	Not screenable
Withdrawn publication	TEMPORARILY WITHDRAWN: Does the shape of the copper intrauterine device play a role in expulsion? Results from the ongoing European Active Surveillance Study on LCS12. Contraception 2023. PMID 37414331	English	Temporarily withdrawn duplicate of the EURAS-LCS12 shape analysis (ID 30)	Withdrawn; the final version is included

Table S7. Protocol elements fixed before the search, post hoc refinements and deviations

OSF registration: DOI 10.17605/OSF.IO/3GUQF.

Element	Registered protocol (fixed before the search)	Refinement or deviation	When / why
Review question and PCC	Fixed	Unchanged; acronym defined in text	—
Framing	Registered as a scoping review	Deviation: reported as a targeted map of the major device programs, randomized trials and large surveillance cohorts rather than as a comprehensive map	Peer review (Reviewer 1, option 2)
Information sources	PubMed/MEDLINE, Scopus, Web of Science Core Collection	Targeted search in PubMed/MEDLINE, Scopus and Web of Science Core Collection (12 October 2026; Scopus and Web of Science added no unique records); ClinicalTrials.gov screened for ongoing trials	Peer review; original yield implausibly low
Search strategy	As registered (Table S3, Part B)	Deviation: original objective-specific blocks replaced by nine program- and domain-specific blocks (Table S3, Part A); original search retained for transparency (Part B)	Peer review
Scope criterion	Not specified	Deviation: for records from the targeted search, eligibility additionally required (a) a named device or trial program report, (b) a randomized trial in a mapped domain or (c) a large registry, claims or surveillance cohort; defined before those records were screened; the 42 original reports retained, two of which (IDs 6 and 37) would not meet it	Peer review; to define the targeted map
Eligibility criteria (i)–(vi)	Fixed	Clarified only: criterion (iii) refers to the evidence domains; eligible designs list names single-arm trials, single-group series and mechanistic studies; withdrawn or retracted records excluded	Peer review
Screening and charting	Two independent reviewers	For the targeted search, first-pass screening, full-text eligibility and charting performed with large-language-model assistance and verified by two reviewers; eligibility confirmed against the full texts	Revision
Specific objectives	As registered	Refined into nine evidence domains before data charting; objective 8 wording extended to endometrial cancer, niche-related bleeding and indomethacin-releasing devices after the targeted search	Organization of the synthesis
Language	English full text required	Non-English records screened using English abstracts and listed (Table S6)	Peer review; transparency
Scope: insertion-pain interventions and established noncontraceptive uses	Not explicitly stated	Explicitly stated as out of scope; discussed as context	Peer review; clarification
Critical appraisal	Design-specific JBI checklists	Deviation: single-arm and single-group reports re-appraised with the case-series checklist; adenomyosis study moved to the quasi-experimental checklist; case–control checklist applied to one added report; mechanistic and modelling reports described without scoring	Peer review; design fit

Element	Registered protocol (fixed before the search)	Refinement or deviation	When / why
Synthesis	Descriptive and thematic; no meta-analysis	Unchanged; confidence intervals around appraisal proportions removed	Peer review
